# Diazazethrene bisimide: a strongly electron-accepting π-system synthesized *via* the incorporation of both imide substituents and imine-type nitrogen atoms into zethrene†

**DOI:** 10.1039/d2sc05992d

**Published:** 2022-12-06

**Authors:** Keita Tajima, Kyohei Matsuo, Hiroko Yamada, Norihito Fukui, Hiroshi Shinokubo

**Affiliations:** a Department of Molecular and Macromolecular Chemistry, Graduate School of Engineering, Integrated Research Consortium on Chemical Science (IRCCS), Nagoya University Furo-cho, Chikusa-ku Nagoya Aichi 464-8603 Japan fukui@chembio.nagoya-u.ac.jp hshino@chembio.nagoya-u.ac.jp; b Division of Material Science, Graduate of School of Science and Technology, Nara Institute of Science and Technology 8916-5 Takayama-cho, Ikoma Nara 630-0912 Japan hyamada@ms.naist.jp; c PRESTO, Japan Science and Technology Agency (JST) Kawaguchi Saitama 332-0012 Japan

## Abstract

The development of highly electron-accepting π-systems is a fundamentally challenging issue despite their potential applications as high-performance n-type organic semiconductors, organic rechargeable batteries, and stable redox-active organocatalysts. Herein, we demonstrate that the incorporation of both imide substituents and imine-type nitrogen atoms into zethrene affords the strongly electron-accepting π-system diazazethrene bisimide (DAZBI). DAZBI has a low-lying LUMO (−4.3 eV *vs.* vacuum) and is readily reduced by the weak reductant l-ascorbic acid to afford the corresponding dihydro species. The injection of two electrons into DAZBI provides the corresponding dianion. These reduced species display remarkable stability, even under ambient conditions, and an intense red fluorescence. A DAZBI dimer, which was also synthesized, effectively accommodated four electrons upon electron injection.

## Abstract 2

The development of highly electron-accepting π-systems is a fundamentally challenging issue despite their potential applications as various functional materials.

## Graphical abstract

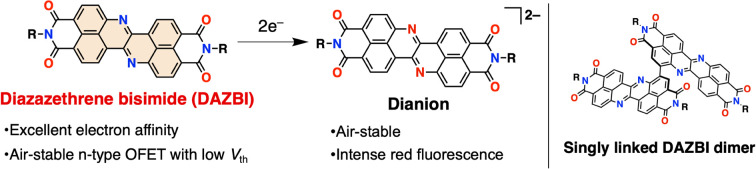

## Introduction

Organic π-conjugated molecules are inherently electron-donating because their π-electrons are weakly bound to the nuclei. Hence, increasing the electron-accepting ability of π-conjugated molecules is an essential challenging issue. Electron-accepting π-systems are highly useful molecules in organic chemistry and materials science. These molecules are predisposed to the injection of electrons, thus enabling their applications in organic electronic devices and photosensitizers. In organic electronic devices, they are used in n-type organic field-effect transistors,^1^ organic photovoltaic devices,^2^ and as active materials in battery electrodes.^3,4^ As photosensitizers, they are key components in photoredox reactions^5,6^ as well as in photodynamic and photothermal therapy.^7^ To create effective electron-accepting π-systems, the stabilization of the lowest unoccupied molecular orbital (LUMO) is crucial. Lowering the LUMO energy is also beneficial for improving the air stability of the reduced species, which often appear as intermediates in the aforementioned materials. Furthermore, decreasing the π-electron density is advantageous for relieving intermolecular exchange repulsion to allow closer molecular contact, thus enhancing electron mobility *via* effective orbital overlap in the solid state.

**Fig. 1:**
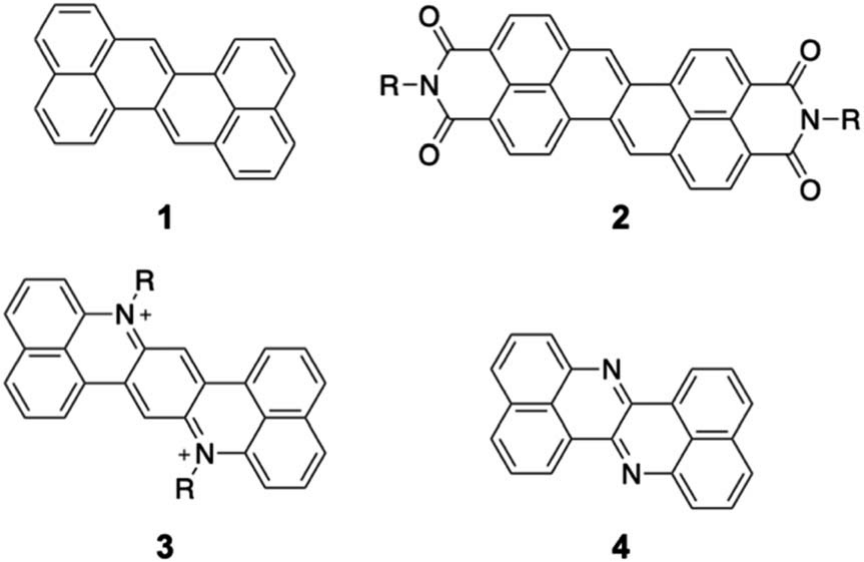
Selected examples of zethrene derivatives.

Zethrene 1 (Fig. 1) is a polycyclic aromatic hydrocarbon with inherent electron-accepting properties that undergoes electron injection at −1.76 V (*vs.* Fc/Fc^+^).^8,9^ Its reduction potential is significantly higher than that of anthanthrene (−2.11 V) despite having a similar composition (zethrene: C_24_H_14_; anthanthrene: C_22_H_12_).^10^ Wu and co-workers have reported zethrene bisimide 2, which undergoes its first reduction at −0.84 V (*vs.* Fc/Fc^+^).^11^ The same group has also synthesized the nitrogen-doped zethrenium dication 3.^12^ Recently, Fujimoto and co-workers have reported nitrogen-doped zethrene 4, which shows, in comparison to zethrene, enhanced electron-affinity and reactivity toward nucleophiles.^13^ These results suggest that the structural modification of zethrene can afford strongly electron-accepting π-systems.

Two effective strategies exist to create highly effective electron-accepting compounds *via* the chemical modification of existing π-systems. One is the functionalization of the periphery with electron-withdrawing imide groups. For example, rylene diimides such as naphthalene diimide (NDI)^14^ and perylene bisimide (PBI)^15^ have significantly lower LUMOs than those of their parent rylenes. The other strategy is the replacement of carbon atoms with more electronegative elements, such as nitrogen atoms.^16–18^ These two strategies have usually been implemented independently.

**Fig. 2:**
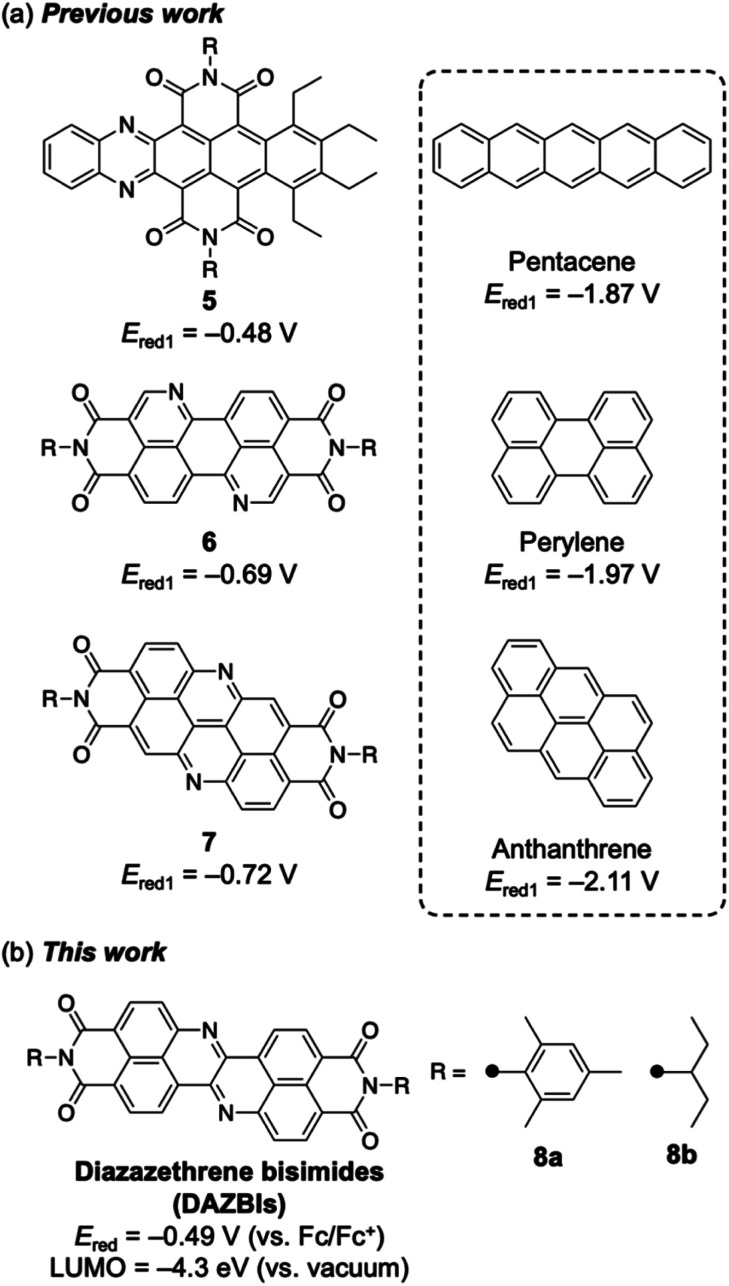
(a) Selected examples of the incorporation of both imide substituents and imine-type nitrogen atoms. (b) Diazazethrene bisimides. The first reduction potentials (*E*_red1_) are standardized relative to the ferrocene/ferrocenium couple (Fc/Fc^+^).

Recently, several researchers have reported the incorporation of both nitrogen atoms and imide substituents to achieve strongly electron-accepting π-systems.^19–27^ Such a dual modification benefits from the aforementioned properties of both the imide groups and the imine-type nitrogen atoms. Selected examples are shown in Fig. 2a. Wang and co-workers have reported the integration of both nitrogen atoms and imide substituents into pentacene 5, which exhibits a relatively high reduction potential (−0.48 V *vs.* Fc/Fc^+^).^19^ Okamoto and co-workers have demonstrated that nitrogen-doped PBI 6 acts as a robust n-type semiconductor.^20–22^ Our groups have recently reported the synthesis of a nitrogen-doped anthanthrene derivative with two imide substituents (7), which exhibits a relatively high electron mobility (0.90 cm^2^ V^−1^ s^−1^) in a single crystal and affords an air-stable radical anion upon electron injection.^23^ In each of these cases, the dual incorporation of two imide groups and two imine-type nitrogen atoms stabilized the LUMO level by *ca.* 1.3 eV. Herein, we report the synthesis and properties of diazazethrene bisimide (DAZBI) 8, a molecule designed during the course of our research into creating new functional π-systems by combining NDI subunits with additional heteroatoms (Fig. 2b).^23,28–30^

## Results and discussion

### Synthesis

**Scheme 1:**
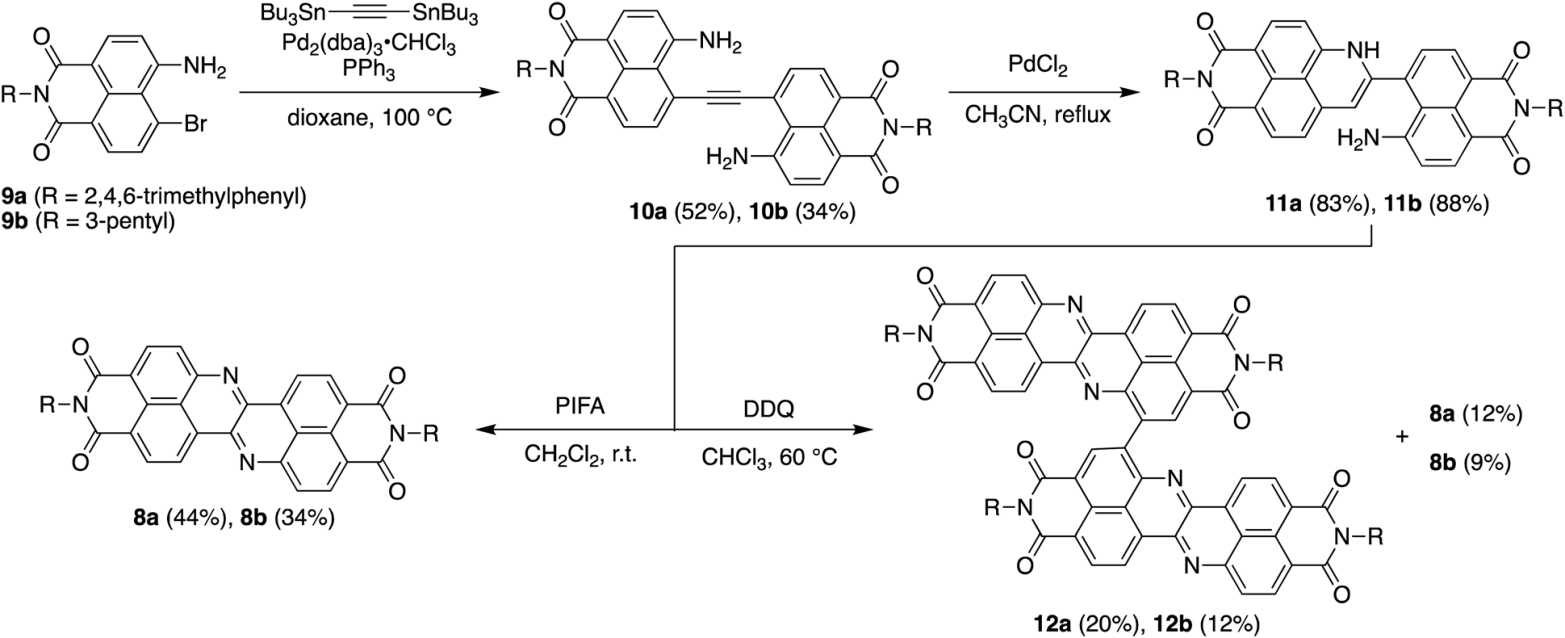
Synthesis of DAZBI monomers and dimers.

Scheme 1 depicts the synthesis of DAZBI 8 starting from the 4-bromo-5-aminonaphthalene monoimides 9a (R = 2,4,6-trimethylphenyl) and 9b (R = 3-pentyl), which were synthesized according to literature methods.^23,31^ The palladium-catalyzed Migita–Kosugi–Stille cross-coupling^32,33^ of 9a and 9b with bis(tributylstannyl)acetylene produced the corresponding ethynylene-linked dimers 10a and 10b in 52% and 34% yield, respectively. The palladium-catalyzed intramolecular hydroamination of 10a and 10b generated precursors 11a and 11b as enamines in 83% and 88% yield, respectively. Subsequent oxidation of the enamines with bis(trifluoroacetoxy)iodobenzene (PIFA) afforded DAZBIs 8a and 8b in 44% and 34% yield, respectively. Interestingly, the treatment of precursors 11a and 11b with 2,3-dichloro-5,6-dicyano-*p*-benzoquinone (DDQ) yielded the directly linked dimers 12a and 12b together with monomers 8a and 8b, respectively. The attempted oxidative dimerization of 8a and 8b with DDQ did not proceed (Fig. S66†). At present, we speculate that the radical coupling of the azaphenalenyl intermediates 11a and 11b generated dimers 12a and 12b (Scheme S1†).

### Structural analysis

**Fig. 3:**
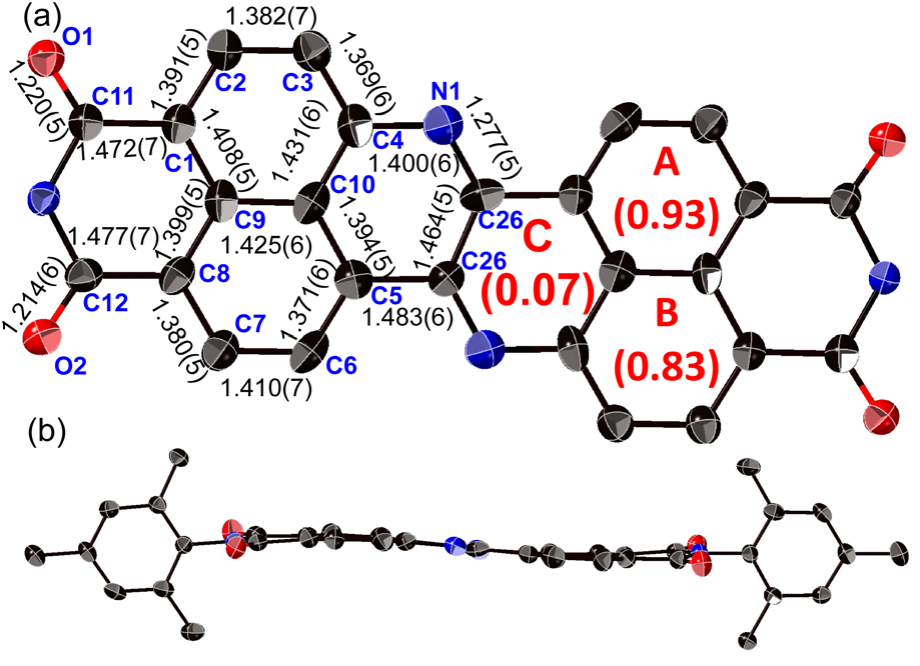
X-ray crystal structure of 8a with thermal ellipsoids at 50% probability. (a) Top view and (b) side view. All hydrogen atoms and solvent molecules are omitted for clarity. In (a), the 2,4,6-trimethylphenyl groups are omitted for clarity.

The structure of 8a was determined unequivocally *via* a single-crystal X-ray diffraction analysis (Fig. 3).^34^ DAZBI 8a adopts a *Z*-shaped structure, wherein the mean plane deviation of the central π-system is 0.044 Å, which indicates high planarity. The length of the C26–N1 bond (1.277(5) Å), which links the central nitrogen atom with the fused ethene unit, is shorter than that of the C4–N1 bond (1.400(6) Å) connecting the central nitrogen atom to the naphthalene monoimide unit. The C4–N1–C26 angle (117.7(4)°) is slightly smaller than that of an ideal hexagon (120°). Furthermore, the harmonic oscillator model of aromaticity (HOMA) value^35^ for ring C (0.07) is smaller than the HOMA values for rings A (0.93) and B (0.83), indicative of a substantial bond-length alternation in ring C. These results suggest the structure is characterized by the contribution of the 1,4-diaza-1,3-butadiene-like structure in the center. A similar trend was observed for the parent zethrene.^9^

**Fig. 4:**
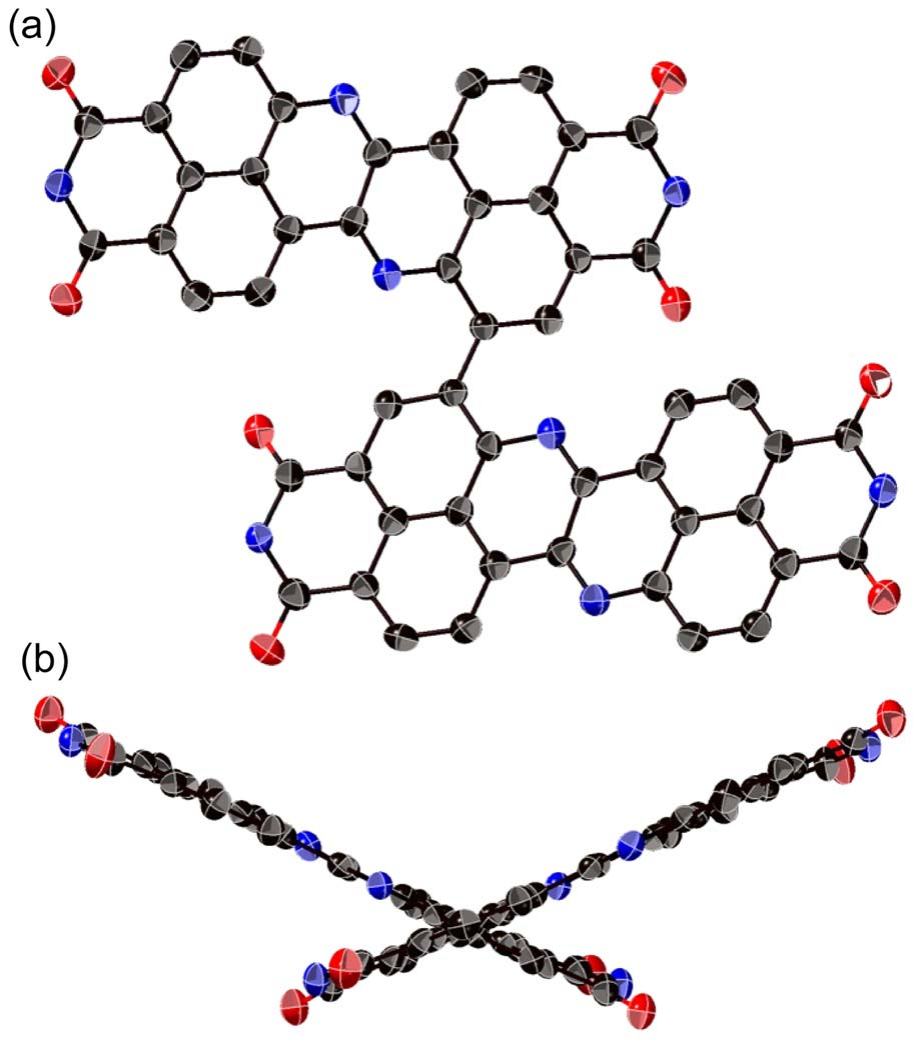
X-ray crystal structure of 12b with thermal ellipsoids at 50% probability. (a) Top view and (b) side view. All hydrogen atoms, the 3-pentyl groups, and solvent molecules are omitted for clarity.

The X-ray crystal structure of dimer 12b is shown in Fig. 4. The two DAZBI moieties are crystallographically equivalent. Each dimer unit was separated by co-crystallized solvent molecules of *o*-xylene. The DAZBI planes are twisted with a dihedral angle of 53°. The mean plane deviation of each DAZBI moiety (0.030 Å) is comparable to that of DAZBI monomer 8a (0.044 Å). Judging from the C11–N2 bond lengths (1.277(5) Å), the central nitrogen atoms retain their imine-type character (Fig. S41†). Interestingly, the HOMA value for ring C in dimer 12b (0.49) is higher than the HOMA value in monomer 8a (0.07), suggesting that the two DAZBI subunits in 12b are efficiently conjugated.

### Optical and electrochemical properties

**Fig. 5:**
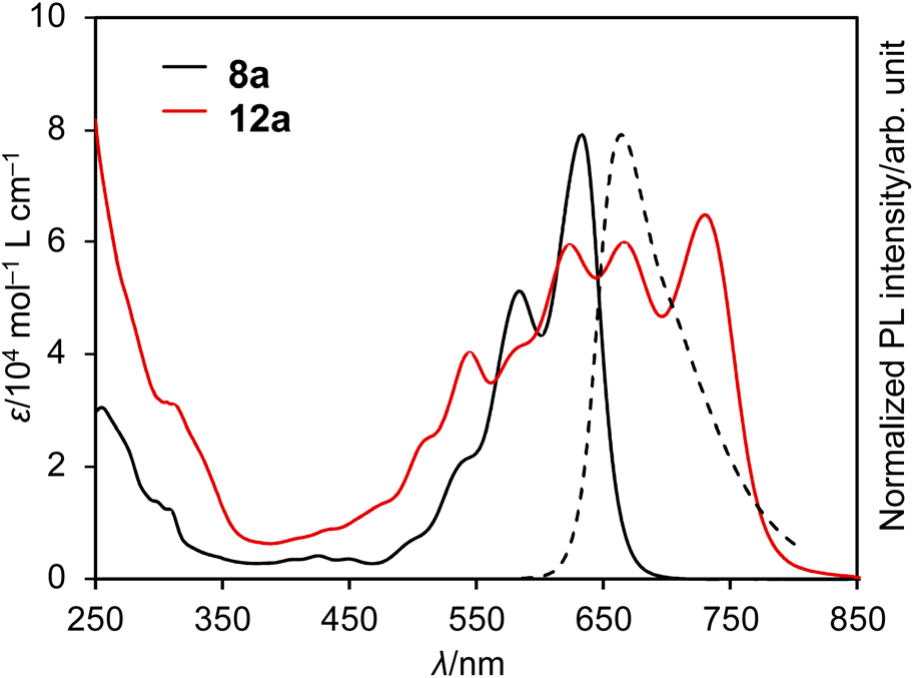
UV/vis absorption spectra of 8a and 12a (solid lines) and emission spectrum of 8a (dashed line); solvent: CH_2_Cl_2_; *λ*: wavelength; *ε*: extinction coefficient; *λ*_ex_ = 550 nm.

The UV/vis absorption and emission spectra of DAZBI monomer 8a and dimer 12a in CH_2_Cl_2_ are shown in Fig. 5. DAZBI monomer 8a exhibits intense and well-resolved absorption bands in the 500–700 nm range. This spectral feature is similar to those observed for zethrene 1 (*λ*_Abs_ = 550 nm),^9^ zethrene bisimide 2 (*λ*_Abs_ = 648 nm),^11^ and 7,14-diazazethrene 4 (*λ*_Abs_ = 568 nm).^13^ The absorption spectrum of DAZBI 8a (*λ*_Abs_ = 633 nm) is drastically red-shifted compared to those of diazaperylene bisimide 6 (*λ*_Abs_ = 520 nm)^20^ and diazaanthanthrene bisimide 7 (*λ*_Abs_ = 497 nm),^23^ indicating that the choice of a zethrene core for the application of the current design concept: the incorporation of both imide substituents and imine-type nitrogen atoms, is critical to realize the responsivity in a long wavelength region. DAZBI 8a moreover shows red fluorescence with a quantum yield of 0.10 and a lifetime of 1.7 ns. These parameters afford a radiative decay rate constant (*k*_r_) and the sum of non-radiative and intersystem crossing decay rate constants (*k*_nr_ + *k*_ISC_) of 6.5 × 10^7^ s^−1^ and 5.0 ×10^8^ s^−1^, respectively. The emission spectrum is the mirror image of the absorption spectrum with a small Stokes shift of 436 cm^−1^. These results, combined with the presence of a clear vibrational structure, confirms that 8a is structurally rigid. On the other hand, the spectrum of DAZBI dimer 12a has multiple absorption bands that are bathochromically shifted relative to those of 8a. The wavelength of the absorption onset (*ca.* 800 nm) is red-shifted compared to that of monomer 8a (*ca.* 700 nm), suggesting that the two DAZBI subunits are efficiently conjugated. The emission spectrum of 12a in solution was too weak to be recorded, probably because the rotation around the single bond between the two DAZBI units induces a fast relaxation of its excited state.

Subsequently, we evaluated the electron affinity of 8a and 12a using cyclic voltammetry (Fig. S43–S45† and Table 1). The voltammograms were obtained in CH_2_Cl_2_ using 0.1 M [Bu_4_N][PF_6_] as the supporting electrolyte and a Ag/AgNO_3_ reference electrode; all potentials are given relative to the ferrocene/ferrocenium couple (Fc/Fc^+^). DAZBI monomer 8a exhibits two reversible reduction waves at −0.49 V and −0.73 V (Table 1, line 1). The first reduction potential of 8a is shifted positively by 0.35 V and 0.84 V relative to those of zethrene bisimide 2 (−0.84 V)^11^ and 7,14-diazazethrene 4 (−1.33 V),^13^ respectively. Furthermore, the first reduction potential of 8a (−0.49 V) is even higher than those of nitrogen-doped PBI 6 (−0.69 V)^20^ and nitrogen-doped anthanthrene bisimide 7 (−0.72 V),^23^ highlighting that the choice of a zethrene core for the application of the current design concept: the incorporation of both imide substituents and imine-type nitrogen atoms, is critical to realize high electron-accepting ability. According to the experimentally obtained reduction potential of 8a, its LUMO level was calculated to be −4.3 eV (*vs.* vacuum), which satisfies the criterion needed to be considered an air-stable electron-transporting material (< −4.0 eV *vs.* vacuum).^36,37^ The oxidation wave of 8a was irreversible and, using differential pulse voltammetry, the first oxidation potential was determined to be 1.24 V (*vs.* Fc/Fc^+^). The electrochemical HOMO–LUMO gap for 8a is 1.73 V. The cyclic voltammogram obtained for 12a shows a reversible four-step reduction process and an irreversible oxidation wave. The first reduction and oxidation potentials are −0.36 V and 1.07 V, respectively (Table 1, line 3). The reduction potential of 12a is equal to that of *p*-chloranil (−0.36 V),^38^ a common mild oxidant. The electrochemical HOMO–LUMO gap for 12a (1.43 V) is narrower than that of DAZBI monomer 8a (1.73 V), which constitutes further evidence for the effective π-conjugation between the two DAZBI units in dimer 12a.

**Table 1:** Redox potentials of 8a, 12a, and 14b. All potentials are standardized to the ferrocene/ferrocenium (Fc/Fc^+^) couple; supporting electrolyte: 0.1 M [Bu_4_N][PF_6_]; reference electrode: Ag/AgNO_3_

	*E*_ox1_/V	*E*_ox2_/V	*E*_ox3_/V	*E*_ox4_/V	*E*_red1_/V	*E*_red2_/V	*E*_red3_/V	*E*_red4_/V	Δ*E*/V
8a^a^	1.24	—	—	—	−0.49	−0.73	—	—	1.73
8a^b^	1.23	—	—	—	−0.44	−0.78	—	—	1.67
12a^a^	1.07	1.20	1.35	1.53	−0.36	−0.48	−0.73	−0.91	1.43
14b^b^	0.10	1.18	—	—	−1.76	−2.05	—	—	1.86

a Conducted in CH_2_Cl_2_.

b Conducted in THF.

To obtain further insight into the electronic structure of DAZBI and its dimer, we conducted density functional theory (DFT) calculations for the DAZBI monomer 8c (R = Me) and its DAZBI dimer 12c (R = Me) at the B3LYP/6-31G(d) level using the Gaussian 09 software package (Fig. S47 and S48†). The initial geometry was used from their X-ray crystal structures.

The HOMO and LUMO of 8c are delocalized over the entire π-system, the distribution of which is similar to those of zethrene 1, zethrene bisimide 2c (R = Me), and diazazethrene 4. Conversely, the LUMO levels decrease drastically in the order 1 (−2.34 eV) > 4 (−2.82 eV) > 2c (−3.67 eV) > 8c (−4.05 eV). This order clearly demonstrates that both the imide functionalities and the sp^2^-hybridized nitrogen atoms significantly stabilize the LUMO level. The LUMO level of 8c (−4.05 eV) is lower than those of diazaperylene bisimide 6 (−3.84 eV) and diazaanthanthrene bisimide 7 (−3.88 eV), which nicely accords with their redox properties. The HOMO level of 8c is stabilized by 1.40 eV compared to that of 1. Consequently, the HOMO–LUMO gap of 8c (Δ*E* = 1.91 eV) is decreased by 0.3 eV upon the dual incorporation of imide substituents and imine-type nitrogen atom. A similar trend has been observed for the comparisons between perylene and diazaperylene bisimide 6 as well as anthanthrene and diazaanthanthrene bisimide 7 (Fig. S49†). On the other hand, the HOMO–LUMO gap of 8c (Δ*E* = 1.91 eV) was narrower than those of 6 (Δ*E* = 2.62 eV) and 7 (Δ*E* = 2.58 eV) (Fig. S49†), which accords with the red-shifted absorption of 8a compared to those of 6 and 7.

The optimized structure of 12c is also twisted with a dihedral angle of 46°, which is close to the experimental value (53°). Nevertheless, the HOMO and HOMO−1 levels of dimer 12c are effectively split with a difference of 0.30 eV due to the anti-bonding and bonding interactions between the HOMOs of the two DAZBI subunits. The same splitting was also observed for the LUMO and LUMO+1 levels with a difference of 0.31 eV. Consequently, the HOMO–LUMO gap of dimer 12c (1.58 eV) is smaller than that of its monomer 8c (1.91 eV). Time-dependent DFT (TD-DFT) calculations showed that the wavelength of the absorption maxima of dimer 12c is red-shifted compared to those of monomer 8c (Fig. S50†). These results corroborate the notion of effective π-conjugation between the two DAZBI subunits in dimer 12c (Fig. S48†).

### Isolation of the reduced species

**Scheme 2:**
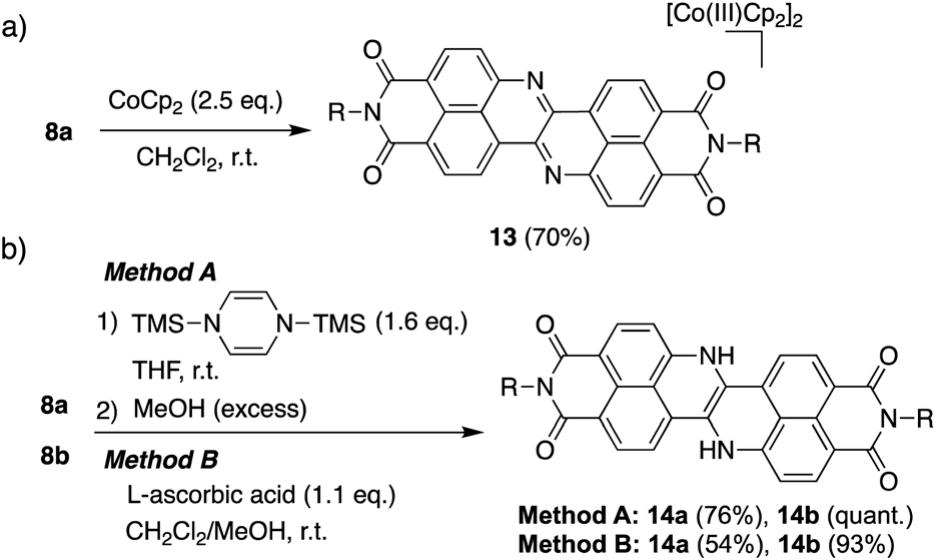
Synthesis of reduced species 13, 14a, and 14b.

The extremely deep-lying LUMO level of DAZBI encouraged us to isolate its reduced species. Radical anions and dianions that are easy to handle are currently attractive targets as materials in photochemical reactions,^14,39,40^ rechargeable batteries,^41^ and photodynamic therapy.^7^ The reaction of 8a with 2.5 equiv. of cobaltocene (CoCp_2_) in CH_2_Cl_2_ at room temperature afforded the corresponding dianion 13 in 70% yield as a green solid (Scheme 2a). The ^1^H NMR spectrum of 13 in DMSO-*d*_6_ showed signals arising from aromatic protons (8.14–6.92 ppm), which are upfield-shifted compared to those of 8a (9.25–8.27 ppm).

We also examined the observation of radical anion. The reductive titration of 8a with cobaltocene resulted in the two-step change of absorption spectra due to the stepwise generation of radical anion and dianion (Fig. S56†), which has been corroborated by spectroelectrochemical analysis (Fig. S57†). However, treatment of 8a with an equimolar amount of cobaltocene in CH_2_Cl_2_ produced a mixture of 8a and 13 because dianion 13 underwent precipitation due to the low solubility in nonpolar solvents, which promoted the disproportionation.

The hydrogenation of 8a and 8b was achieved by sequential treatment with 1,4-bis(trimethylsilyl)-1,4-dihydropyrazine^42^ and MeOH, affording the corresponding dihydro species 14a and 14b in excellent yield (Scheme 2b).^43^ The same transformation was accomplished using a weaker reductant, l-ascorbic acid, at room temperature. The ^1^H NMR spectrum of 14a showed a broad signal at 11.1 ppm, which was assigned to the NH protons (Fig. S70†).

Surprisingly, both dianion 13 and the dihydro species 14a are stable in the presence of O_2_ and H_2_O, thus allowing easy handling under ambient conditions. Moreover, 13 and 14a exhibited negligible degradation in commercially purchased DMSO with half-lives of 46 and 44 days, respectively (Fig. S67 and S68†). Notably, the high stability of 13 is outstanding because (1) the synthesis and isolation of organic dianions usually requires strictly inert conditions,^44–46^ (2) even PBI dianions are sensitive toward atmospheric oxygen,^47–49^ and (3) the stabilization of naphthalene monoimide dianion requires a sophisticated molecular design such as the introduction of cationic peripheral substituents.^50^

**Fig. 6:**
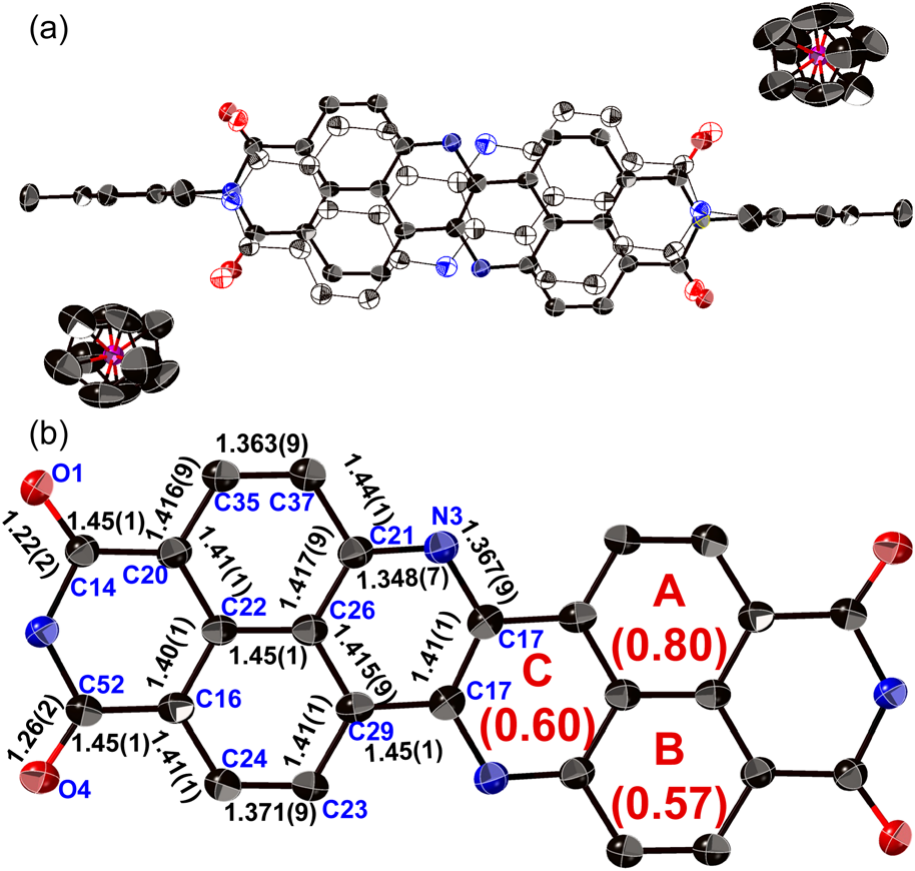
X-ray crystal structure of 13 with thermal ellipsoids at 50% probability. Hydrogen atoms and solvent molecules are omitted for clarity. (a) Hollow and solid ellipsoids show the disorder over two positions (occupancy: solid spheres/hollow spheres = 0.59/0.41). (b) The structure shows only the position with higher occupancy, whereby the 2,4,6-trimethylphenyl groups and cobaltocenium ions are omitted for clarity.

The structure of dianion 13 was determined unequivocally using single-crystal X-ray diffraction analysis (Fig. 6). Each molecule is disordered over two positions with relative occupancies of 0.59 and 0.41, whereby the inversion proceeds along the shorter molecular axis. The main skeleton is highly planar with a mean plane deviation of 0.046 Å. The dianion core is separated from the two counter ions ([Co(iii)Cp_2_]^+^), suggesting that there is negligible interaction between these ions. The C17–N3 bond length (1.367(9) Å) is comparable to the C21–N3 bond length (1.348(7) Å), consistent with an amine-type character of the central nitrogen atoms. The HOMA value of ring C (0.60) in 13 is higher than that of 8a (0.07). These results indicate that the injection of two electrons into 8a causes the relaxation of the bond-length alternation. The C–O bonds in 13 (1.22(2)/1.26(2) Å) are longer than those in 8a (1.220(5)/1.214(6) Å), suggesting a weakened carbonyl vibration. This notion was corroborated using infrared (IR) absorption spectroscopy (13: 1634 cm^1^; 8a: 1699 cm^−1^; Fig. S52†) and theoretical calculations (Fig. S54†).

The cyclic voltammogram of 14b in THF shows the first reduction and oxidation waves at −1.76 V and 0.10 V (*vs.* Fc/Fc^+^), respectively (Fig. S46† and Table 1, line 4). These potentials are shifted negatively compared to those of neutral 8a (Table 1, line 2). The electrochemical HOMO–LUMO gap (1.86 V) is slightly larger than that of the DAZBI monomer 8a (1.67 V).

**Fig. 7:**
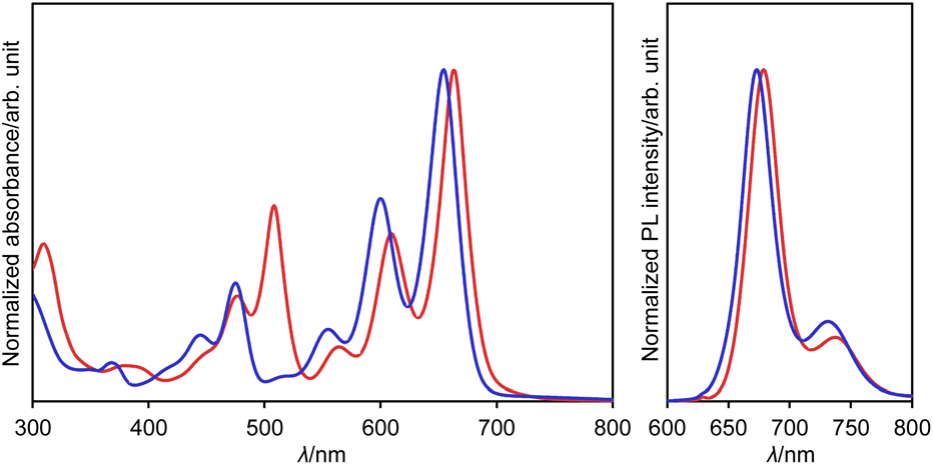
UV/vis absorption and emission (*λ*_ex_ = 630 nm) spectra of 13 (red line) in DMSO and 14a (blue line) in DMSO with one drop of TFA; *λ*: wavelength.

The UV/vis absorption and emission spectra of dianion 13 and dihydro species 14a in DMSO are shown in Fig. 7. One drop of trifluoroacetic acid (TFA) was added to the solution of 14a in order to suppress any spontaneous deprotonation (Fig. S69†). The photophysical parameters are summarized in Table S3.† The absorption and emission spectra of 13 resemble those of 14a, except for an absorption at 508 nm. This similarity suggests highly similar electronic structures for these two compounds. These spectra were also reproduced well using TD-DFT calculations (Fig. S51†). The absorption and emission spectra of 13 and 14a are well-resolved with small stokes shifts of 333 and 408 cm^−1^, respectively, suggesting a small structural relaxation in their excited states. Importantly, dianion 13 and dihydro species 14a exhibit a red fluorescence with a quantum yield of 0.53 and 0.40, respectively. These values are higher than those of DAZBI 8 (0.10) and the PBI dianion (0.18).^47–49^ Given that DAZBI undergoes hydrogenation when treated with l-ascorbic acid to afford the highly emissive reduced species 14, it should be possible to use DAZBI as a fluorescent sensor that responds to a reductive environment in biological systems.^51^

**Fig. 8:**
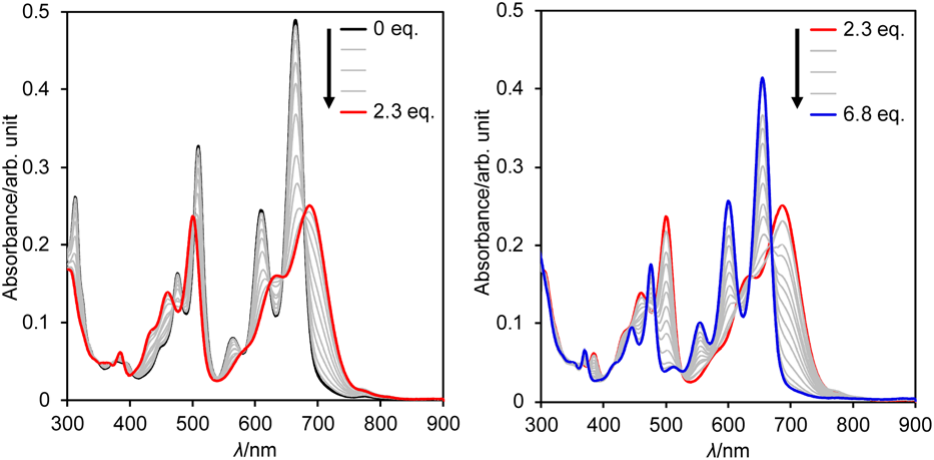
Change in the absorption spectrum of 13 in DMSO upon addition of TFA; *λ*: wavelength.

Subsequently, we examined the interconversion between dianion 13 and dihydro species 14a. Titration of a DMSO solution of 13 with TFA resulted in a two-step change of its absorption spectrum with clear isosbestic points (Fig. 8). The resulting absorption spectrum is identical to that of 14a. The addition of 1,8-diazabicyclo[5.4.0]undec-7-ene (DBU) to this solution retrieved the original spectrum of 13. This reversible acid–base response was also monitored using ^1^H NMR spectroscopy (Fig. S71†).

### Electron-conducting properties

An X-ray diffraction analysis of 3-pentyl-substituted DAZBI 8b showed a quasi-one-dimensional packing structure with a π–π distance of 3.48(1) Å (Fig. S42†). Each molecule is disordered over two positions with relative occupancies of 0.58 and 0.42, whereby the inversion proceeds along the shorter molecular axis. The transfer integrals (*t*) between the LUMOs in its packing structure were simulated using DFT calculations at the PBEPBE/6-31G(d) level (Fig. S65 and Table S5†). Due to the crystal disorder, the transfer integrals must be considered for three combinations. The major and minor disordered structures are defined as 8b-A and 8b-B, respectively. The *t* values along the π-stacking direction are 89.1 meV for 8b-A–8b-A, 7.6 meV for 8b-A–8b-B, and 37.2 meV for 8b-B–8b-B. Smaller *t* values (0.4–2.5 meV) were estimated along the horizontal directions.

Bottom-gate top-contact organic field-effect transistor (OFET) devices were fabricated from 8b. An Al_2_O_3_/SiO_2_ dielectric layer substrate was treated with a self-assembled monolayer of 12-cyclohexyldodecylphosphonic acid (CDPA),^52^ before a thin layer of 8b was vacuum-deposited on the substrate (*ca.* 5 × 10^−4^ Pa 0.3 Å s^−1^). Gold electrodes were vacuum-deposited on the active layer as the source and drain electrodes. A polarization-optical-micrograph (POM) analysis of the deposited film afforded a dark image. An atomic-force-microscopy (AFM) analysis showed a smooth surface with tiny domains (Fig. S61†). Out-of-plane X-ray diffraction measurements did not show any obvious peaks (Fig. S62†). These results indicate that the thin layer is amorphous. Photoelectron-yield-spectroscopy measurements revealed an ionization potential of 5.75 eV (Fig. S63†). Using the onset of the absorption spectrum, the band gap of the obtained thin film was determined to be 1.68 eV (Fig. S64†). According to these results, the energy level of the conduction band was estimated to be −4.07 eV, which matches well with the LUMO level of DAZBI.

**Fig. 9:**
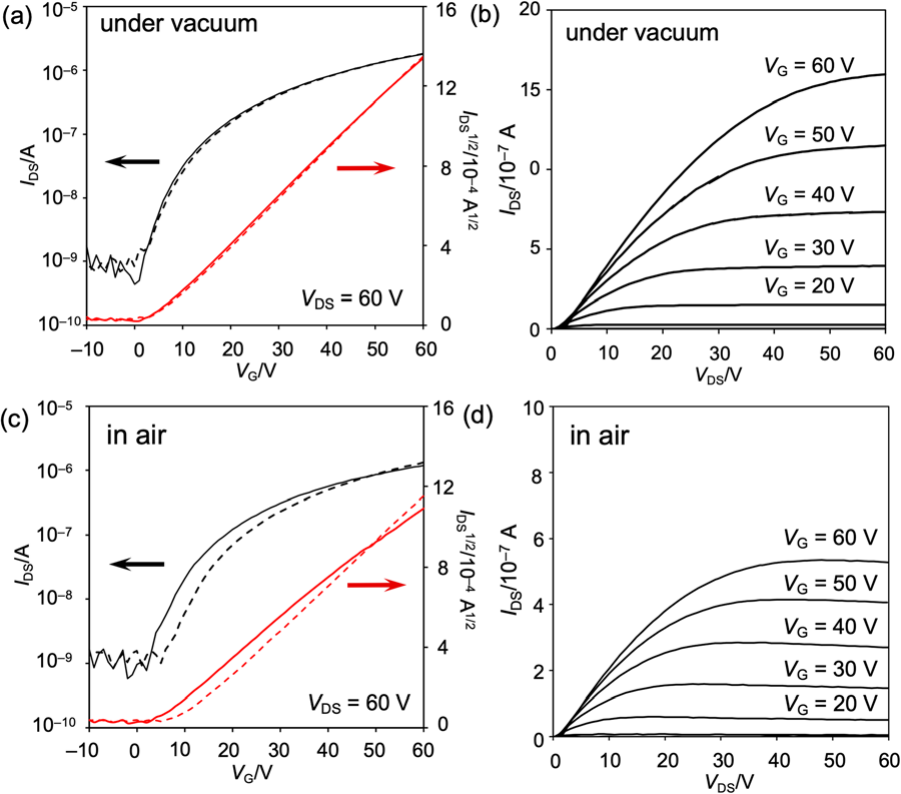
Properties of the vacuum-deposited OFET device fabricated from 8b (a, b) *in vacuo* and (c, d) under atmospheric conditions. (a, c) Transfer characteristics and (b, d) output characteristics.

The properties of the OFET device were measured at ambient temperature *in vacuo* (*ca.* 3 × 10^−1^ Pa) and under atmospheric conditions (Fig. 9 and S60 as well as Table S4†). The obtained OFET device exhibited typical n-type behavior. Under vacuum conditions, the maximum and average electron mobilities *μ*_e_ are 6.7 × 10^−3^ and (6.1 ± 0.5) × 10^−3^ cm^2^ V^−1^ s^−1^, respectively, with an average on/off current ratio *I*_on_/*I*_off_ of (3.4 ± 1.8) × 10^3^. Negligible current hysteresis was observed in the transfer characteristics. Notably, the devices exhibited a low threshold voltage, *V*_th_, of 2 V, reflecting the extraordinary electron affinity of 8b. Furthermore, exposure to air scarcely decreased the electron mobility, leading to a maximum and average electron mobility *μ*_e_ of 5.4 × 10^−3^ and (4.6 ± 0.7) × 10^−3^ cm^2^ V^−1^ s^−1^, respectively. The current hysteresis was negligible, even under atmospheric conditions.

## Conclusions

We synthesized the two diazazethrene bisimides (DAZBIs) 8a and 8b, which are nitrogen-doped zethrene derivatives with electron-withdrawing imide substituents. Moreover, the two DAZBI dimers 12a and 12b were prepared from 8a and 8b, respectively. Notably, DAZBI monomer 8a exhibits an extraordinary electron affinity with a first reduction potential of −0.49 V. DAZBI dimer 12a has an even higher electron affinity and the ability to accept multiple electrons due to the efficient electronic conjugation between its two DAZBI units. Treatment of DAZBI with cobaltocene afforded dianion 13. Hydrogenation of DAZBI proceeded, even with l-ascorbic acid, to provide the dihydro species 14. These reduced species exhibit both remarkable stability under ambient conditions and bright red fluorescence. In addition, these two reduced species are interconvertible upon treatment with acid and base. A thin film of DAZBI is able to function as an air-stable n-type organic semiconductor with a low threshold voltage. Notably, DAZBI 8 is attractive compared to the structurally resembled species 6 and 7 in light of the superior electron-accepting ability and narrower HOMO–LUMO gap. The present study demonstrates that the choice of the main skeleton (herein zethrene) is crucial upon applying the design concept of the dual incorporation of both imide substituents and imine-type nitrogen atoms in order to design various functional materials with superb electron-accepting properties.

## Data availability

Crystallographic data for 8a, 8b, 12b, and 13 has been deposited at the CCDC under 2211115, 2211112, 2211113, and 2211114, respectively, and can be obtained from https://www.ccdc.cam.ac.uk/. The datasets supporting this article have been uploaded as part of the ESI material.†

## Author contributions

The manuscript was written through contributions of all authors. All authors have approved of the final version of the manuscript. H. S. and N. F. designed and conducted the project and finalized the manuscript. K. T. carried out all the experiments including the synthesis, characterization, and OFET-fabrication. K. T. also wrote the first draft. K. M. and H. Y. supervised the evaluation of OFET characteristics.

## Conflicts of interest

There are no conflicts to declare.

## Supplementary Material

SC-014-D2SC05992D-s001

SC-014-D2SC05992D-s002
