# Supplementary material for: Diazazethrene bisimide: a strongly electron-accepting π-system synthesized via the incorporation of both imide substituents and imine-type nitrogen atoms into zethrene
Source: Chem Sci. 2022 Dec 6;14(3):635–42. doi: 10.1039/d2sc05992d (PMC9847653; doi:10.1039/d2sc05992d)
Supplement: SC-014-D2SC05992D-s001 [file SC-014-D2SC05992D-s001.pdf]

Electronic Supplementary Material (ESI) for Chemical Science.
This journal is © The Royal Society of Chemistry 2022

Supporting Information

Diazazethrene Bisimide: A Strongly Electron-Accepting π-System Synthesized via the Incorporation of both Imide Substituents and Imine-type Nitrogen Atoms into Zethrene

Keita Tajima,[1] Kyohei Matsuo,[2] Hiroko, Yamada,*[2] Norihito Fukui,*[1,3] and Hiroshi Shinokubo*[1]

[1]Department of Molecular and Macromolecular Chemistry, Graduate School of Engineering, and Integrated Research Consortium on Chemical Science (IRCCS), Nagoya University, Furo-cho, Chikusa-ku, Nagoya, Aichi 464-8603, Japan

[2]Division of Materials Science, Graduate School of Science and Technology, Nara Institute of Science and Technology, 8916-5 Takaya-ma-cho, Ikoma, Nara 630-0192, Japan

[3]PRESTO, Japan Science and Technology Agency (JST), Kawaguchi, Saitama 332-0012 (Japan)

E-mail: hyamada@ms.naist.jp, fukui@chembio.nagoya-u.ac.jp, hshino@chembio.nagoya-u.ac.jp

Table of Contents

1. Instrumentation and materials 3

2. Experimental procedures and compound data 4

3. NMR spectra 14

4. Mass spectra 40

5. Crystal data 54

6. Electrochemistry 58

7. DFT calculations 61

8. IR spectra 64

9. Photophysical properties 66

10. OFET devices 69

11. Other properties 74

12. References 82

1. Instrumentation and materials

^{1}H NMR (500 MHz, 600 MHz) and ^{13}C NMR (126 MHz, 151 MHz) spectra were recorded on a Varian INOVA-500 and JEOL JNM-ECA600II spectrometer. Chemical shifts were reported as the delta scale in ppm relative to $CHCl_3$ (δ = 7.26 ppm) and DMSO-d_6 (δ = 2.50 ppm) for ^{1}H NMR, and $CDCl_3$ (δ = 77.16 ppm) and DMSO-d_6 (δ = 39.52 ppm) for ^{13}C NMR. UV/vis/NIR absorption spectra were recorded on a JASCO V-670 spectrometer. Emission spectra were recorded on a JASCO FP-6500 spectrometer, and absolute fluorescence quantum yields were measured by the photon-counting method using an integration sphere. Preparative separations were performed by silica gel column chromatography (Wako gel® C-300 or C-400, and FUJISILYSIA CHROMATOREX NH−DM1020). High-resolution atmospheric pressure chemical ionization time-of-flight (APCI-TOF) and electrospray ionization time-of-flight (ESI-TOF) mass spectra were taken on a Bruker micrOTOF instrument using a positive or negative ionization mode. High-resolution matrix-assisted laser desorption and ionization time-of-flight (MALDI-TOF) mass spectra were taken on a Bruker autoflex max using a positive ionization mode. Redox potentials were measured by cyclic voltammetry on an ALS electrochemical analyzer model 612C. X-ray data were obtained using a Rigaku CCD diffractometer (Saturn 724 with MicroMax-007) with Varimax Mo optics. Dry CH_2Cl_2 was prepared by distillation from CaH_2. Dry THF and 1,4-dioxane were prepared by using GlassContour™ solvent purification systems. Cobaltocene purchased from a commercial supplier was purified by sublimation. An oil bath was used as a heat source for reactions requiring heating. Unless otherwise noted, materials obtained from commercial suppliers were used without further purification. 4-Amino-5-bromo-*N*-(3-pentyl)-1,8-naphthalenedicarboxylic imide **9b** was synthesized according to the literature.[S1]

2. Experimental procedures and compound data

N-(2,4,6-Trimethylphenyl)-4-bromo-5-nitro-1,8-naphthalenedicarboxylic monoamide S1

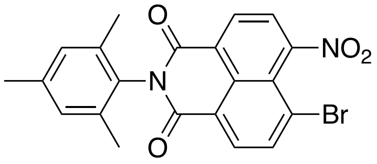

4-Bromo-5-nitro-1,8-naphthalenedicarboxylic anhydride (3.22 g, 10.0 mmol) and acetic acid (200 mL) were placed in a round-bottom flask. The mixture was stirred at room temperature for 3 min. To the flask, 2,4,6-trimethylaniline (2.67 g, 19.8 mmol) was added. The mixture was refluxed for 13 h. The resulting mixture was cooled to room temperature. The reaction was quenched by the addition of HCl aq. (1 M). The precipitations were collected by filtration and washed with water and MeOH. Recrystallization from CH_2Cl_2/hexane afforded compound **S1** (2.62 g, 5.94 mmol, 59%) as a beige solid.

^{1}H NMR (500 MHz, $CDCl_3$, 298 K): δ = 8.77 (d, J = 7.8 Hz, 1H), 8.58 (d, J = 8.0 Hz, 1H), 8.26 (d, J = 8.0 Hz, 1H), 7.97 (d, J = 7.8 Hz, 1H), 7.05 (s, 2H), 2.36 (s, 3H), 2.08 (s, 6H) ppm; ^{13}C NMR (126 MHz, $CDCl_3$, 298 K): δ = 162.35, 161.60, 151.68, 139.27, 136.19, 135.02, 132.88, 131.85, 131.30, 130.29, 129.67, 125.89, 124.73, 123.75, 122.60, 121.59, 21.30, 17.87 ppm; HRMS (APCI-TOF, positive mode): $[M+H]^+$ Calcd for $C_{21}H_{16}N_2O_4{}^{79}Br$ 439.0288; Found 439.0285.

N-(2,4,6-Trimethylphenyl)-4-bromo-5-amino-1,8-naphthalenedicarboxylic monoamide 9a

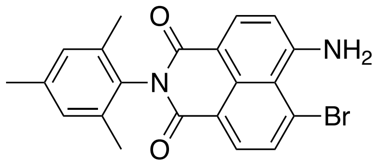

Compound **S1** (1.76 g, 4.00 mmol) and ethanol (80 mL) were placed in a round-bottom flask. The mixture was stirred at room temperature for 3 min. A solution of $SnCl_2$ (3.79 g, 20.1 mmol) in conc. HCl aq. (15 mL) was added slowly to the mixture. The mixture was refluxed for

1 h. The mixture was filtered and washed with water, affording **9a** (1.50 g, 3.68 mmol, 92%) as a yellow solid.

^{1}H NMR (500 MHz, DMSO-d_6, 298 K): δ = 8.26 (d, J = 8.6 Hz, 1H), 8.24 (d, J = 8.0 Hz, 1H), 7.94 (d, J =8.0 Hz, 1H), 7.66 (s, 2H), 7.07 (d, J = 8.6 Hz, 1H), 6.99 (s, 2H), 2.29 (s, 3H), 1.93 (s, 6H) ppm; ^{13}C NMR (126 MHz, DMSO-d_6, 298 K): δ = 162.74, 162.09, 153.06, 137.25, 134.99, 134.21, 132.85, 131.97, 131.80, 131.45, 128.67, 126.31, 121.66, 116.68, 111.74, 108.34, 20.62, 17.32 ppm; HRMS (APCI-TOF, positive mode): $[M+H]^+$ Calcd for $C_{21}H_{18}N_2O_2{}^{79}Br$ 409.0546; Found 409.0563.

Ethynylene-bridged aminonaphthalene monoimide dimer 10a

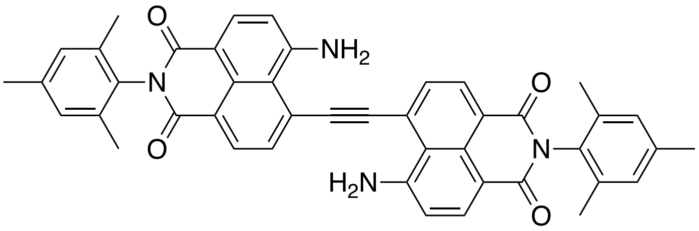

A round-bottom flask containing compound **9a** (853 mg, 2.09 mmol), $Pd_2(dba)_3$•$CHCl_3$ (108 mg, 0.106 mmol), and triphenylphosphine (110 mg, 0.419 mmol) was purged with N_2 and then charged with dry dioxane (100 mL). Bis(tributylstannyl)acetylene (625 mg, 1.03 mmol) was added to the mixture. The mixture was stirred at 100 °C for 3 h. The resulting mixture was cooled to room temperature. The mixture was filtered, and the residue was washed with hexane. The residue was suspended in $CHCl_3$ (100 mL), and then hexane (50 mL) was added to the mixture. The precipitates collected by filtration and washed with hexane, affording **10a** (373 mg, 547 μmol, 52%) as a reddish-purple solid.

^{1}H NMR (500 MHz, DMSO-d_6, 298 K): δ = 8.51 (d, J = 7.6 Hz, 2H), 8.32 (d, J = 8.4 Hz, 2H), 8.10 (d, J = 7.6 Hz, 2H), 7.65 (s, 4H), 7.11 (d, J = 8.4 Hz, 2H), 7.02 (s, 4H), 2.31 (s, 6H), 1.96 (s, 12H) ppm; ^{13}C NMR (126 MHz, DMSO-d_6, 298 K): δ = 162.67, 162.23, 152.96, 137.28, 135.02, 134.27, 132.00, 131.19, 130.48, 128.70, 123.18, 122.83, 118.04, 111.25, 108.61, 98.51, 20.63, 17.34 ppm (One signal is missing due to overlapping); HRMS (MALDI-TOF, positive mode, DIT matrix): $[M+H]^+$ Calcd for $C_{44}H_{35}N_4O_4$ 683.2653; Found 683.2681.

Compound 11a

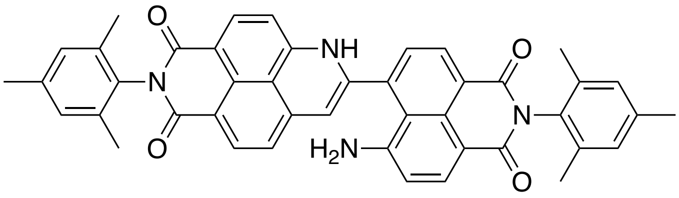

Compound **10a** (342 mg, 501 μmol), $PdCl_2$ (17.7 mg, 99.8 μmol), and acetonitrile (250 mL) were placed in a round-bottom flask. The mixture was refluxed for 1 h. The resulting mixture was cooled to room temperature. The mixture was filtered, and residue was washed with acetonitrile and hexane, affording **11a** (286 mg, 418 μmol, 83%) as a reddish brown solid.

^{1}H NMR (500 MHz, DMSO-d_6, 333 K): δ = 12.17 (s, 1H, The signal was weakened by the addition of D_2O), 8.61 (d, J = 7.5 Hz, 1H), 8.38 (d, J = 8.5 Hz, 1H), 8.33 (d, J = 8.0 Hz, 1H), 8.28 (d, J = 8.5 Hz, 1H), 7.81 (d, J = 7.5 Hz, 1H), 7.20 (d, J = 8.0 Hz, 1H), 7.15 (d, J = 8.5 Hz, 1H), 7.03 (s, 2H), 7.01 (d, J = 8.5 Hz, 1H, overlapped), 7.00 (s, 2H), 6.92 (s, 1H), 6.47 (s, 2H), 2.33 (s, 3H), 2.32 (s, 3H), 2.01 (s, 6H), 1.96 (s, 6H) ppm; ^{13}C NMR (126 MHz, DMSO-d_6, 333 K): δ = 162.52, 162.26, 162.07, 161.94, 152.11, 145.08, 144.18, 141.66, 136.96, 136.95, 136.39, 134.75, 134.68, 133.67, 133.28, 133.18, 132.97, 131.81, 131.60, 131.16, 130.42, 128.36, 128.28, 123.20, 116.77, 114.64, 113.04, 111.53, 109.25, 109.04, 108.75, 105.78, 20.66, 17.36, 17.30 ppm (Three signals are missing due to overlapping); HRMS (ESI-TOF, negative mode): $[M–H]^-$ Calcd for $C_{44}H_{34}N_4O_4$ 681.2496; Found 681.2498.

DAZBI 8a

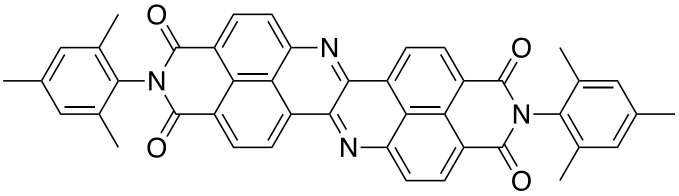

Compound **11a** (67.8 mg, 99.3 μmol), [bis(trifluoroacetoxy)iodo]benzene (129 mg, 301 μmol), and CH_2Cl_2 (50 mL) were placed in a round-bottom flask. The mixture was stirred at room temperature for 2 h. The mixture was filtered over a pad of silica gel eluted with CH_2Cl_2/AcOEt (v/v = 60/1). After removal of the solvent *in vacuo*, the residue was separated by

silica gel column chromatography eluted with CH_2Cl_2 to afford compound **8a** (29.5 mg, 43.5 μmol, 44%) as a dark green solid.

^{1}H NMR (500 MHz, $CDCl_3$, 298 K): δ = 9.25 (d, *J* = 7.9 Hz, 2H), 8.87 (d, *J* = 7.9 Hz, 2H), 8.79 (d, *J* = 7.9 Hz, 2H), 8.27 (d, *J* = 7.9 Hz, 2H), 7.08 (s, 4H), 2.38 (s, 6H), 2.14 (s, 12H) ppm; ^{13}C NMR (126 MHz, $CDCl_3$, 298 K): *δ* = 162.95, 162.61, 149.91, 146.01, 139.02, 135.23, 133.89, 133.55, 131.78, 130.94, 130.93, 129.65, 129.11, 126.90, 125.65, 123.41, 122.28, 21.36, 17.99 ppm; HRMS (MALDI-TOF, positive mode, TCNQ matrix): $[M+H]^+$ Calcd for $C_{44}H_{31}N_4O_4$ 679.2340; Found 679.2361.

DAZBI dimer12a

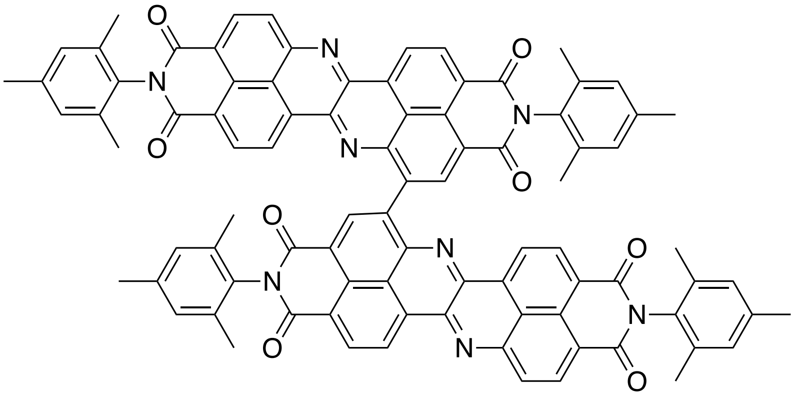

Compound **11a** (68.0 mg, 99.7 μmol), 2,3-dichloro-5,6-dicyano-*p*-benzoquinone (68.1 mg, 300 μmol), and CH_2Cl_2 (50 mL) were placed in a round-bottom flask. The mixture was stirred for 3 h at 60 °C. The mixture was filtered over a pad of silica gel eluted with CH_2Cl_2/AcOEt (v/v = 40/1). After removal of the solvent *in vacuo*, the residue was separated by silica gel column chromatography eluted with $CHCl_3$ to afford **12a** (13.6 mg, 10.1 μmol, 20%) as a black solid and compound **8a** (8.00 mg, 11.8 μmol, 12%) as a dark green solid.

^{1}H NMR (500 MHz, $CDCl_3$, 333 K): δ = 9.51 (s, 2H), 9.39 (d, *J* = 8.0 Hz, 2H), 8.99 (d, *J* = 8.0 Hz, 2H), 8.87 (d, *J* = 8.0 Hz, 2H), 8.78 (d, *J* = 7.9 Hz, 2H), 8.62 (d, *J* = 8.0 Hz, 2H), 8.31 (d, *J* = 7.9 Hz, 2H), 7.11 (s, 4H), 7.05 (s, 4H), 2.39 (s, 6H), 2.38 (s, 6H), 2.29 (s, 12H), 2.11 (s, 12H) ppm; ^{13}C NMR (126 MHz, $CDCl_3$, 333 K): *δ* = 162.97, 162.93, 162.78, 162.45, 149.59, 149.54, 146.36, 144.02, 139.40, 139.14, 138.99, 137.64, 135.38, 135.28, 134.58, 133.98, 133.51, 132.10, 131.68, 131.42, 131.20, 131.13, 129.82, 129.63, 129.32, 129.17, 127.46, 127.21, 126.02, 125.96, 123.88, 123.18, 122.43, 122.04, 21.27, 21.26, 18.02, 17.91 ppm; HRMS (MALDI-TOF,

positive mode, TCNQ matrix): $[M+H]^+$ Calcd for $C_{88}H_{59}N_8O_8$ 1355.4450; Found 1355.4458.

Ethynylene-bridged aminonaphthalene monoimide dimer 10b

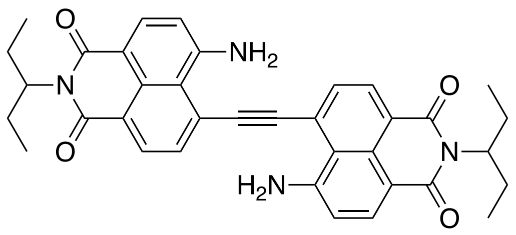

A round-bottom flask containing compound **9b** (181 mg, 500 µmol), $Pd_2(dba)_3$•$CHCl_3$ (25.2 mg, 24.9 µmol), and triphenylphosphine (26.1 mg, 99.6 µmol) was purged with N_2, and then charged dry dioxane (25 mL). Bis(tributylstannyl)acetylene (153 mg, 253 µmol) was added to the mixture. The mixture was stirred at 100 °C for 3 h. After removal of the solvent *in vacuo*, the residue was separated by silica gel column chromatography eluted with CH_2Cl_2/AcOEt (v/v = 10/1) to afford compound **10b** (49.5 mg, 84.4 µmol, 34%) as a red solid.

^{1}H NMR (500 MHz, $CDCl_3$, 333 K): δ = 8.56 (d, J = 7.6 Hz, 2H), 8.45 (d, J = 8.2 Hz, 2H), 7.89 (d, J = 7.6 Hz, 2H), 6.90 (d, J = 8.2 Hz, 2H), 6.05 (s, 4H), 5.03 (tt, J_1 = 9.3 Hz, J_2 = 5.9 Hz, 2H), 2.24 (qdd, J_1 = 7.5 Hz, J_2 = 13.8 Hz, J_3 = 9.3 Hz, 4H), 1.92 (qdd, J_1 = 7.5 Hz, J_2 = 13.8 Hz, J_3 = 5.9 Hz, 4H), 0.91 (t, J_1 = 7.5 Hz, 12H) ppm.; ^{13}C NMR (126 MHz, $CDCl_3$, 333 K): δ = 164.69, 164.61, 150.21, 134.44, 132.29, 131.37, 130.37, 125.08, 121.96, 118.99, 113.09, 112.13, 98.53, 57.70, 25.36, 11.44 ppm.; HRMS (MALDI-TOF, positive mode, DIT matrix): $[M+H]^+$ Calcd for $C_{36}H_{35}N_4O_4$ 587.2653; Found 587.2642.

Compound 11b

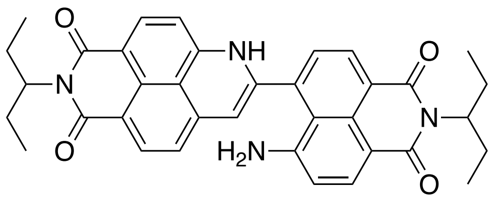

Compound **10b** (95.5 mg, 163 µmol), $PdCl_2$ (5.79 mg, 32.7 µmol), and acetonitrile (80 mL) were placed in a round-bottom flask. The mixture was refluxed for 1 h. After removal of the

solvent *in vacuo*, the residue was washed with acetonitrile and hexane, affording **11b** (84.1 mg, 143 μmol, 88%) as a red solid.

^{1}H NMR (500 MHz, DMSO-d_6, 333 K): δ = 12.01 (s, 1H, The signal was weakened by the addition of D_2O), 8.53 (d, J = 7.5 Hz, 1H), 8.33 (d, J = 8.4 Hz, 1H), 8.26 (d, J = 8.0 Hz, 1H), 8.23 (d, J = 8.4 Hz, 1H), 7.75 (d, J = 7.5 Hz, 1H), 7.13 (d, J = 8.0 Hz, 1H), 7.08 (d, J = 8.4 Hz, 1H), 6.94 (d, J = 8.4 Hz, 1H), 6.81 (s, 1H), 6.34 (s, 2H, The signal was weakened by the addition of D_2O), 5.03 (tt, J_1 = 9.5 Hz, J_2 = 5.8 Hz, 1H), 4.95 (tt, J_1 = 9.6 Hz, J_2 = 5.8 Hz, 1H), 2.19 (m, 4H), 1.86 (qdd, J_1 = 7.5 Hz, J_2 = 11.4 Hz, J_3 = 5.8 Hz, 2H), 1.83 (qdd, J_1 = 7.5 Hz, J_2 = 11.4 Hz, J_3 = 5.8 Hz, 2H), 0.83 (t, J_1 = 7.5 Hz, 6H), 0.79 (t, J_1 = 7.5 Hz, 6H) ppm; ^{13}C NMR (126 MHz, DMSO-d_6, 298 K): δ = 163.71, 163.63, 163.44, 163.31, 151.40, 144.57, 143.99, 141.00, 136.34, 133.40, 133.09, 133.02, 130.83, 130.54, 129.99, 128.06, 123.32, 122.82, 116.38, 114.32, 113.06, 111.35, 109.30, 109.04, 108.59, 105.46, 55.80, 55.35, 24.22, 24.05, 10.73, 10.66 ppm; HRMS (ESI-TOF, negative mode): $[M–H]^-$ Calcd for $C_{36}H_{34}N_4O_4$ 585.2496; Found 585.2508.

DAZBI 8b

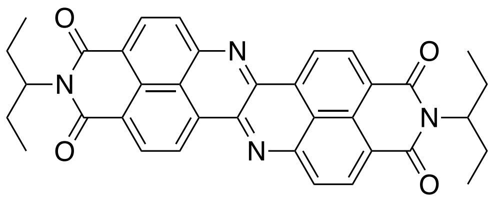

Compound **11b** (35.2 mg, 60.0 μmol), [bis(trifluoroacetoxy)iodo]benzene (77.5 mg, 180 μmol), and CH_2Cl_2 (30 mL) were placed in a round-bottom flask. The mixture was stirred at room temperature for 2 h. The mixture was separated by silica gel column chromatography eluted with CH_2Cl_2/AcOEt (v/v = 35/1) to afford compound **8b** (12.0 mg, 20.6 μmol, 34%) as a green solid.

^{1}H NMR (500 MHz, $CDCl_3$, 298 K): δ = 9.13 (d, J = 8.0 Hz, 2H), 8.75 (d, J = 8.0 Hz, 2H), 8.66 (d, J = 7.8 Hz, 2H), 8.14 (d, J = 7.8 Hz, 2H), 5.08 (tt, J_1 = 9.3 Hz, J_2 = 6.0 Hz, 2H), 2.24 (qdd, J_1 = 7.5 Hz, J_2 = 14.0 Hz, J_3 = 9.3 Hz, 4H), 1.92 (qdd, J_1 = 7.5 Hz, J_2 = 13.8 Hz, J_3 = 6.0 Hz, 4H), 0.91 (t, J_1 = 7.5 Hz, 12H) ppm; ^{13}C NMR (126 MHz, $CDCl_3$, 333 K): δ = 164.27, 163.98, 149.95, 145.71, 133.53, 133.03, 131.34, 130.68, 128.70, 126.73, 126.06, 123.82, 122.05, 58.28, 25.38, 11.50 ppm; HRMS (MALDI-TOF, positive mode, TCNQ matrix): $[M]^+$ Calcd for $C_{36}H_{30}N_4O_4$ 582.2262; Found 582.2255.

DAZBI dimer 12b

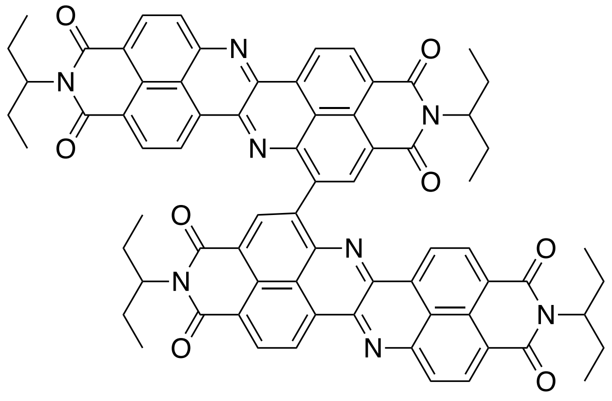

Compound **3b** (37.4 mg, 63.7 µmol), 2,3-dichloro-5,6-dicyano-*p*-benzoquinone (43.3 mg, 191 µmol), and CH_2Cl_2 (30 mL) were placed in a round-bottom flask. The mixture was stirred for 3 h at 60 °C. The mixture was filtered over a pad of silica gel eluted with CH_2Cl_2. After removal of the solvent *in vacuo*, the residue was separated by silica gel column chromatography eluted with CH_2Cl_2 to afford **12b** (4.57 mg, 3.93 µmol, 12%) as a black solid and compound **8b** (3.37 mg, 5.78 µmol, 9%) as a green solid.

^{1}H NMR (500 MHz, $CDCl_3$, 313 K): δ = 9.29 (s, 2H), 9.18 (d, J = 8.0 Hz, 2H), 8.82 (d, J = 8.0 Hz, 2H), 8.66 (t, J = 8.0 Hz, 2H), 8.60 (d, J = 7.9 Hz, 2H), 8.44 (d, J = 8.0 Hz, 2H), 8.13 (d, J = 7.9 Hz, 2H), 5.14 (tt, J_1 = 9.3 Hz, J_2 = 6.0 Hz, 2H), 4.98 (tt, J_1 = 9.3 Hz, J_2 = 6.0 Hz, 2H), 2.34 (m, 4H), 2.18 (qdd, J_1 = 7.5 Hz, J_2 = 14.0 Hz, J_3 = 9.3 Hz, 4H), 2.05 (m, 4H), 1.93 (m, 4H), 1.08 (t, J = 7.5 Hz, 12H) 0.90 (d, J = 7.5 Hz, 12H) ppm; ^{13}C NMR (126 MHz, $CDCl_3$, 313 K): δ = 164.16, 163.67 (br, overlapped), 149.09, 149.07, 145.70, 143.37, 139.05, 137.35, 133.80, 133.35, 133.03, 131.61, 131.17, 131.01, 128.59, 128.47, 127.17, 126.87, 125.99, 125.89, 123.74, 122.52, 121.90, 121.83, 58.35, 58.16, 25.30, 25.20, 11.65, 11.49 ppm; HRMS (MALDI-TOF, positive mode, TCNQ matrix): $[M+H]^+$ Calcd for $C_{72}H_{59}N_8O_8$ 1163.4450; Found 1163.4428.

Dianion 13

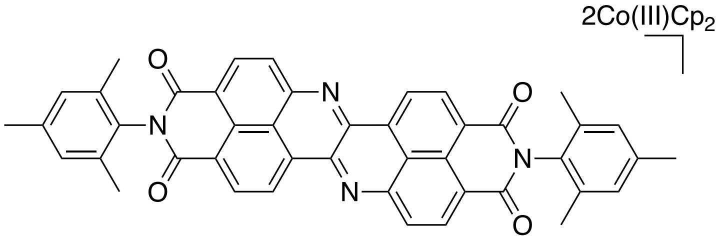

In an argon-filled glovebox, compound **8a** (13.8 mg, 20.3 μmol), cobaltocene (9.7 mg, 14 μmol), and dry THF (4 mL) were placed in a round-bottom flask. The mixture was stirred at room temperature for 1 h. The precipitates were collected by filtration and washed with benzene. Recrystallization from CH_3CN/benzene afforded compound **13** (15.1 mg, 14.3 μmol, 70%) as a dark green solid.

^1H NMR (500 MHz, DMSO-d_6, 298 K): δ = 8.14 (d, J = 8.5 Hz, 2H), 8.09 (d, J = 8.5 Hz, 2H), 7.79 (d, J = 9.0 Hz, 2H), 6.96 (s, 4H), 6.92 (d, J = 9.0 Hz, 2H), 5.80 (s, 20H, Cp), 2.31 (s, 6H), 1.91 (s, 12H) ppm.; ^{13}C NMR (126 MHz, DMSO-d_6, 298 K): δ = 162.17, 161.04, 150.57, 137.86, 135.49, 135.42, 135.33, 135.23, 135.10, 129.25, 128.90, 128.25, 123.95, 115.17, 110.70, 110.55, 100.25, 84,86 (Cp), 20.64, 17.62 ppm.; HRMS (ESI-TOF, negative mode): $[M^{2-}+H]^-$ Calcd for $C_{44}H_{31}N_4O_4$ 679.2340; Found 679.2323; HRMS (ESI-TOF, positive mode): $[CoCp_2]^+$ Calcd for $C_{10}H_{10}Co$ 189.0109; Found 189.0110.

Dihydro-form 14a

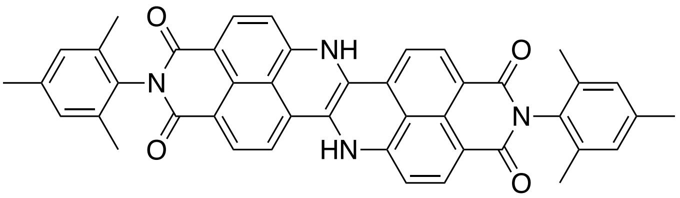

(Method A) In an-argon filled glovebox, compound **8a** (3.51 mg, 5.17 μmol), 1,4-bis(trimethylsilyl)-1,4-dihydropyrazine (2.0 mg, 8.8 μmol), and dry THF (2 mL) were placed in a round-bottom flask. The mixture was stirred at room temperature for 30 min. MeOH (2 mL) was added to the reaction mixture. The precipitates were collected by filtration and washed with methanol, affording compound **14a** (2.66 mg, 3.91 μmol, 76%) as a dark green solid.

(Method B) Compound **8a** (6.73 mg, 9.99 μmol) and CH_2Cl_2 (5 mL) were placed in a round-bottom flask. A MeOH solution (1.8 mL) of L-ascorbic acid (6.1 mM) was added to the

reaction mixture. The mixture was stirred for 20 min at room temperature. After removal of the solvent *in vacuo*, the residue was washed with methanol to afford compound **14a** (3.67 mg, 5.39 μmol, 54%) as a green solid.

^{1}H NMR (500 MHz, DMSO-d_6+CF_3COOH, 298 K): δ = 11.14 (s, 2H, The signal was weakened by the addition of D_2O), 8.39 (d, *J* = 8.5 Hz, 2H), 7.97 (d, *J* = 8.5 Hz, 2H), 7.75 (d, *J* = 8.5 Hz, 2H), 7.03 (s, 4H), 6.92 (d, *J* = 8.5 Hz, 2H), 2.33 (s, 6H), 2.01 (s, 12H) ppm; ^{13}C NMR (126 MHz, DMSO-d_6+CF_3COOH, 298 K): *δ* = 162.36, 161.90, 142.05, 136.90, 134.95, 132.91, 132.26, 131.23, 130.75, 128.68, 122.79, 122.01, 116.37, 114.06, 111.38, 108.38, 106.45, 20.63, 17.44 ppm; HRMS (MALDI-TOF, positive mode, DIT matrix): $[M+H]^+$ Calcd for $C_{44}H_{33}N_4O_4$ 681.2496; Found 681.2520.

Dihydro-form 14b

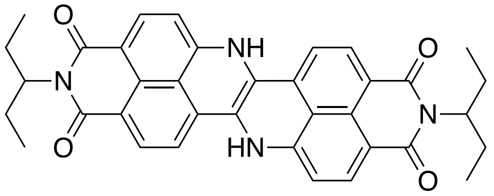

(Method A) In an argon-filled glovebox, compound **8b** (2.97 mg, 5.10 μmol), 1,4-bis(trimethylsilyl)-1,4-dihydropyrazine (2.3 mg, 10 μmol), and dry THF (2 mL) were placed in a round-bottom flask. The mixture was stirred at room temperature for 30 min. MeOH (2 mL) was added to reaction mixture. The precipitates were collected by filtration and washed with methanol to afford compound **14b** (2.99 mg, 5.45 μmol, quant.) as a dark green solid.

(Method B) Compound **8b** (5.83 mg, 10.0 μmol) and CH_2Cl_2 (5 mL) were placed in a round-bottom flask. A MeOH solution (1.8 mL) of L-ascorbic acid (6.1 mM) was added to the reaction mixture. The mixture was stirred for 20 min at room temperature. After removal of the solvent *in vacuo*, the residue was washed with methanol to afford compound **14b** (5.42 mg, 9.27 μmol, 93%) as a green solid.

^{1}H NMR (500 MHz, DMSO-d_6+CF_3COOH, 323 K): δ = 11.42 (s, 2H, The signal was weakened by the addition of D_2O), 8.42 (d, *J* = 8.2 Hz, 2H), 8.21 (d, *J* = 8.5 Hz, 2H), 7.94 (d, *J* = 8.2 Hz, 2H), 7.29 (d, *J* = 8.5 Hz, 2H), 5.02 (m, 2H), 2.20 (m, 4H), 1.85 (sep, *J* = 7.5 Hz, 4H), 0.81 (d, *J* = 7.5 Hz, 12H) ppm.; ^{13}C NMR (151 MHz, DMSO-d_6+CF_3COOH, 323 K): *δ* = 162.36 (br, overlapped), 142.06, 132.54, 130.63, 122.86, 121.99, 117.94, 116.02, 114.10, 112.18, 111.34,

106.63, 55.69, 24.18, 10.99 ppm.; HRMS (MALDI-TOF, positive mode, DIT matrix): $[M+H]^+$ Calcd for $C_{36}H_{33}N_4O_4$ 585.2496; Found 585.2496.

3. NMR spectra

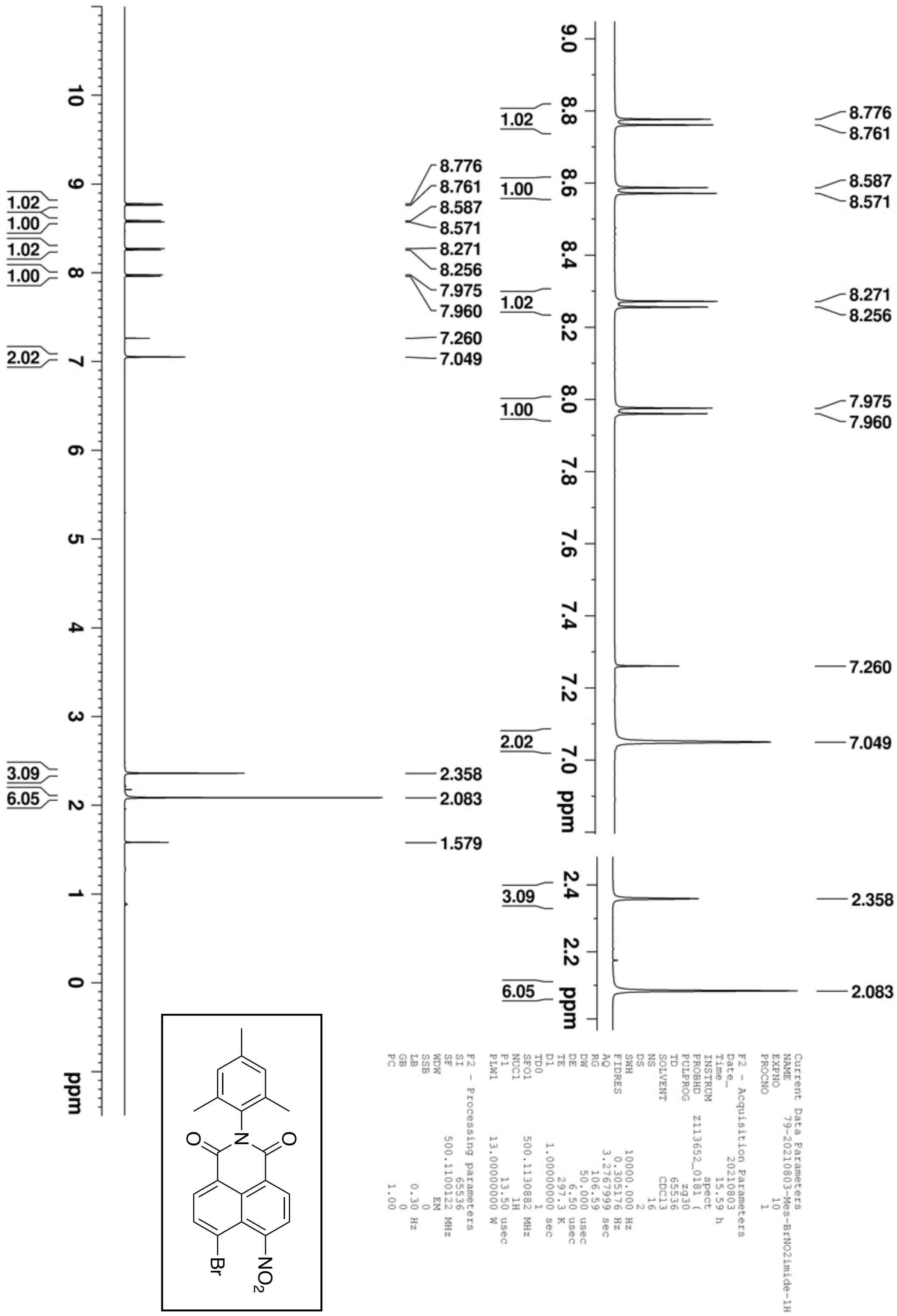

Figure S1. ^{1}H NMR spectrum of **S1** in $CDCl_3$ at 25 °C.

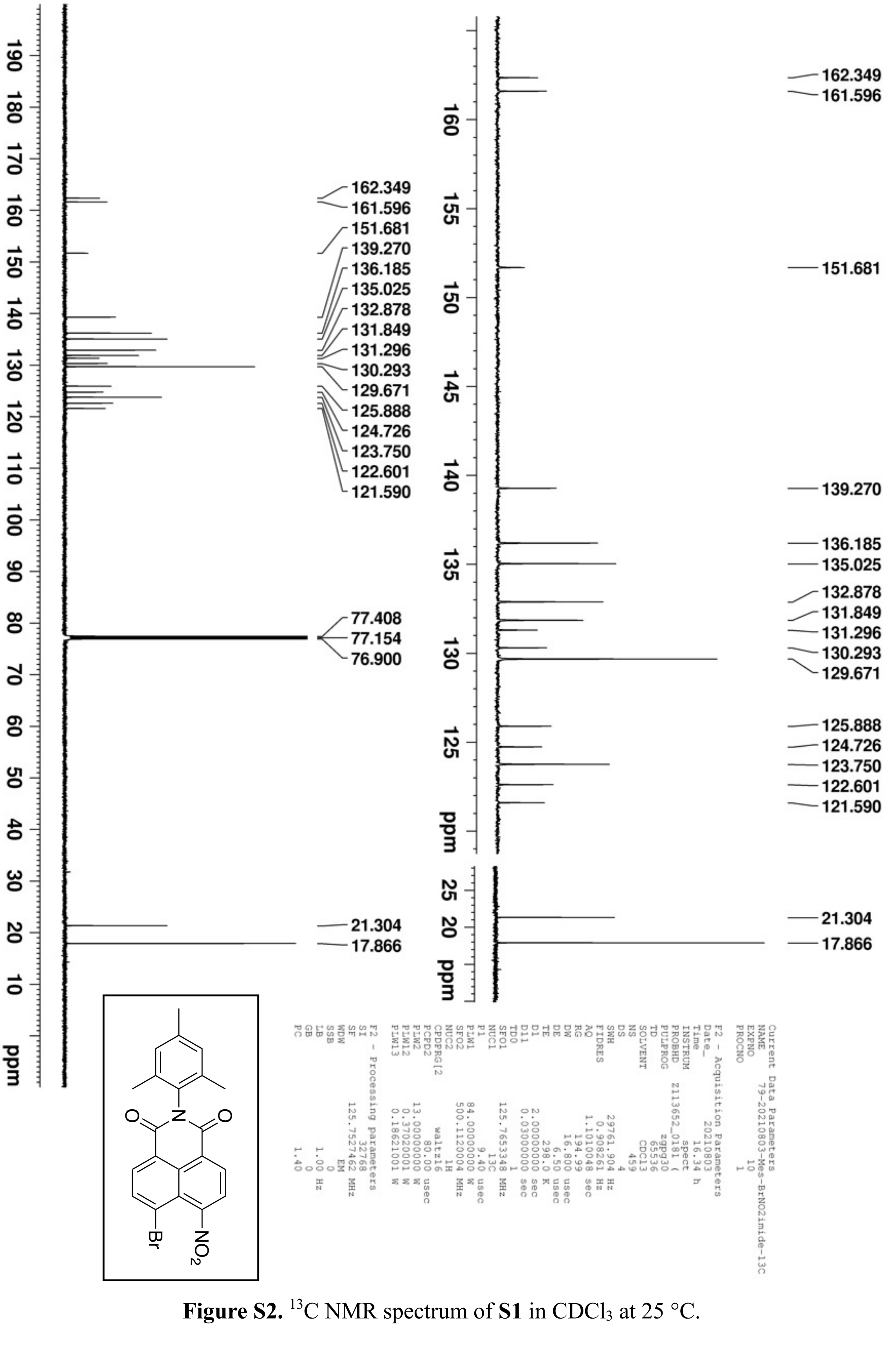

Figure S2. ^{13}C NMR spectrum of **S1** in $CDCl_3$ at 25 °C.

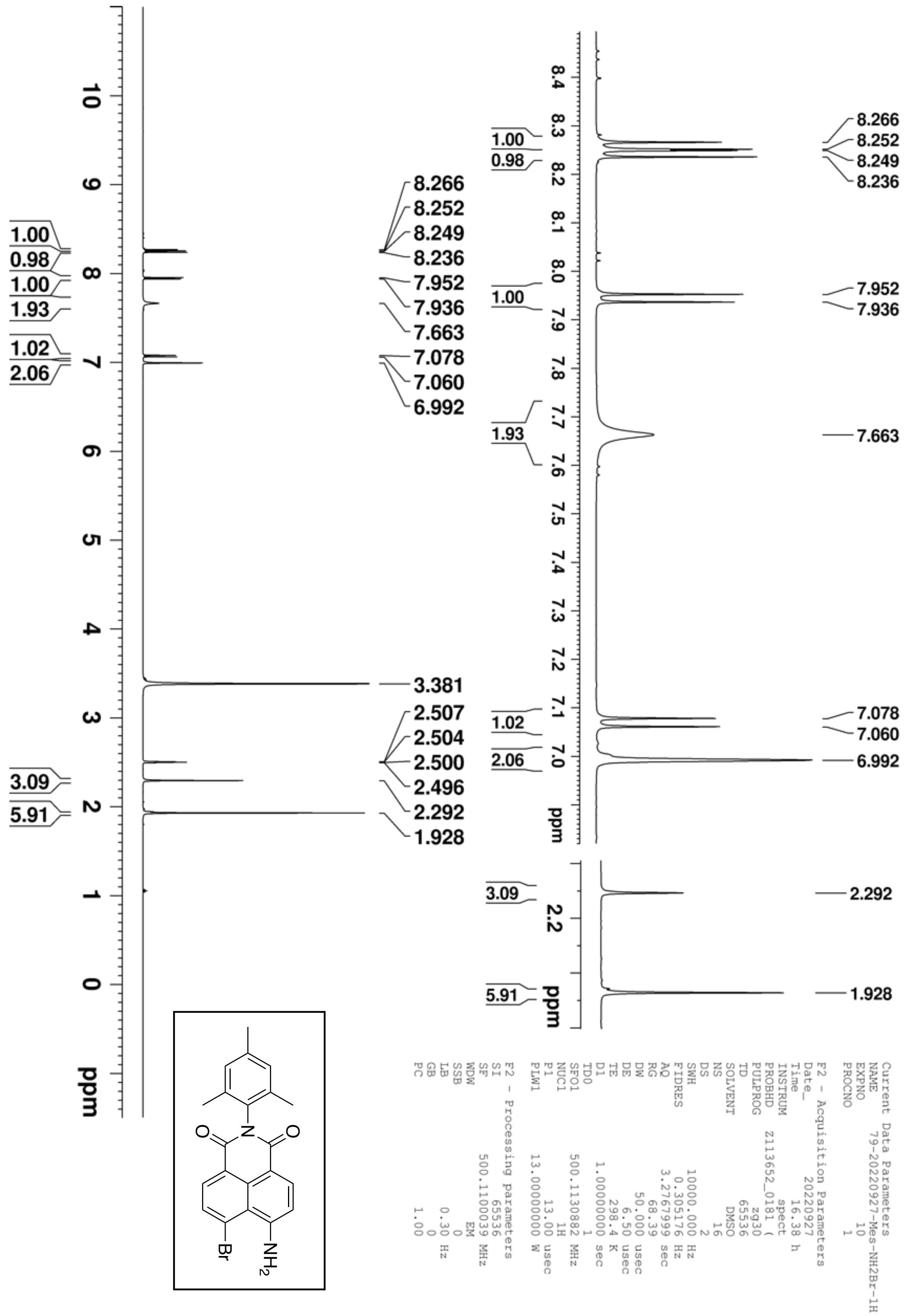

Figure S3. ^{1}H NMR spectrum of **9a** in DMSO-d_6 at 25 °C.

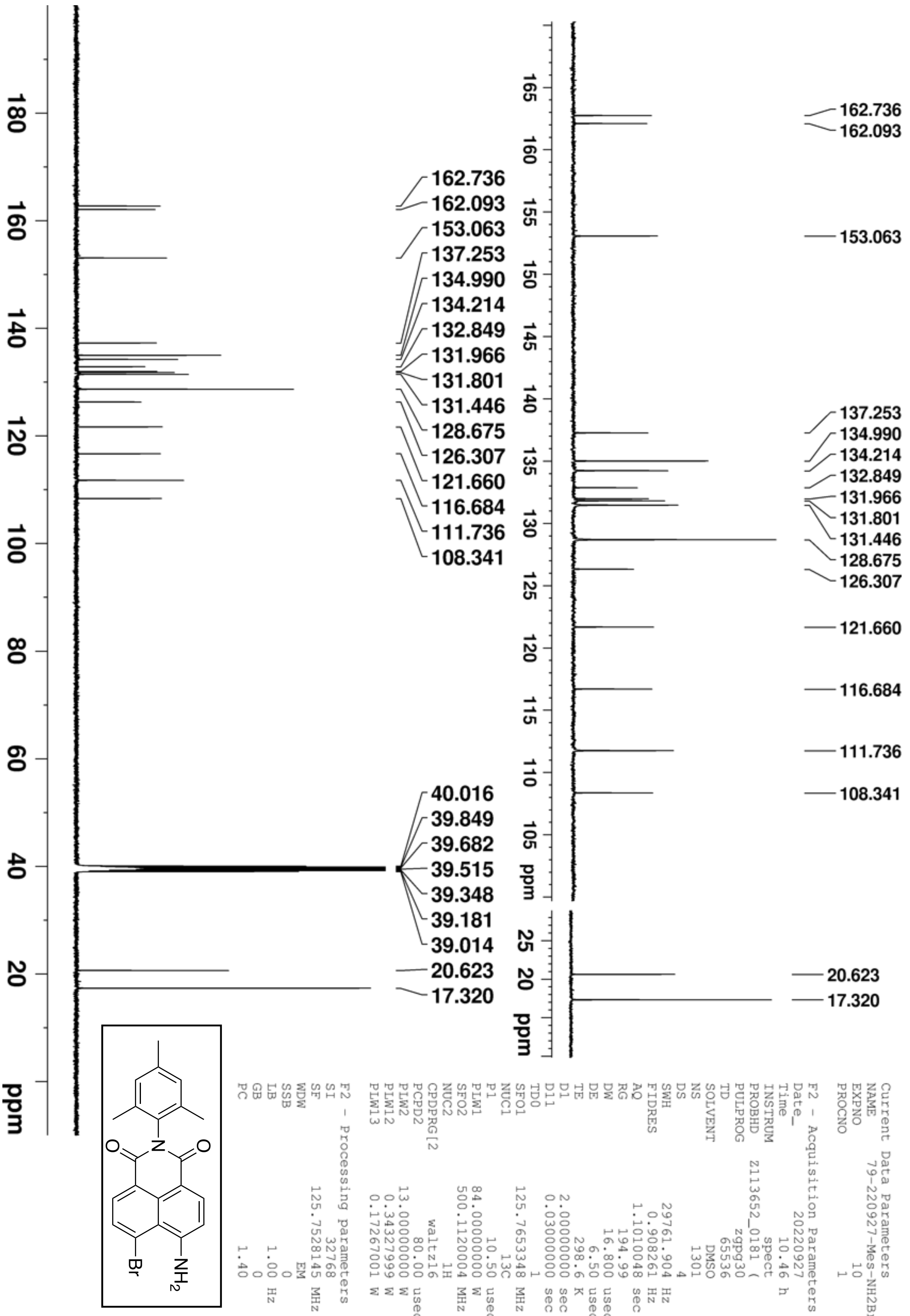

Figure S4. ^{13}C NMR spectrum of **9a** in DMSO-d_6 at 25 °C.

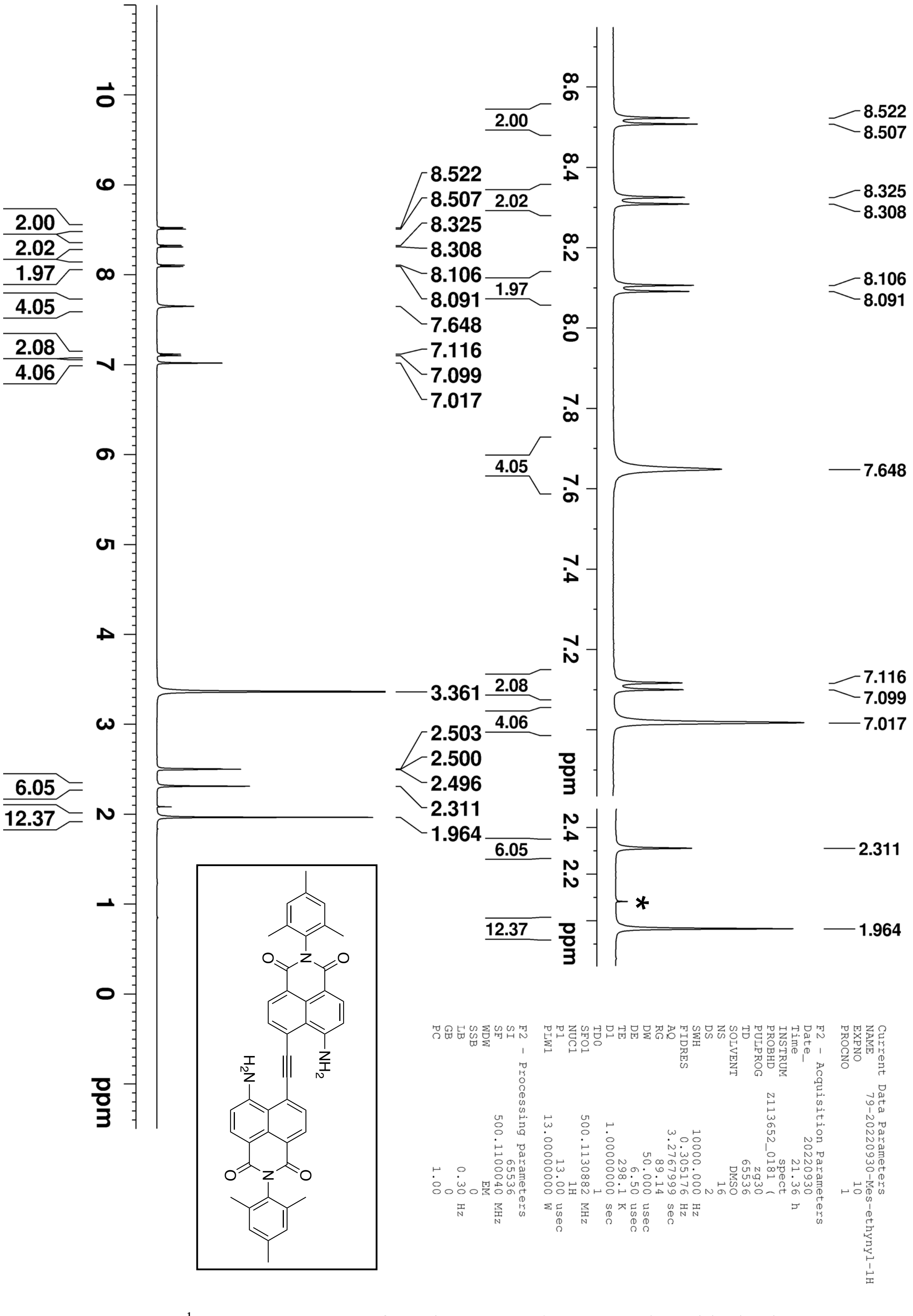

Figure S5. ^{1}H NMR spectrum of **10a** in DMSO-d_6 at 25 °C. *: residual solvents.

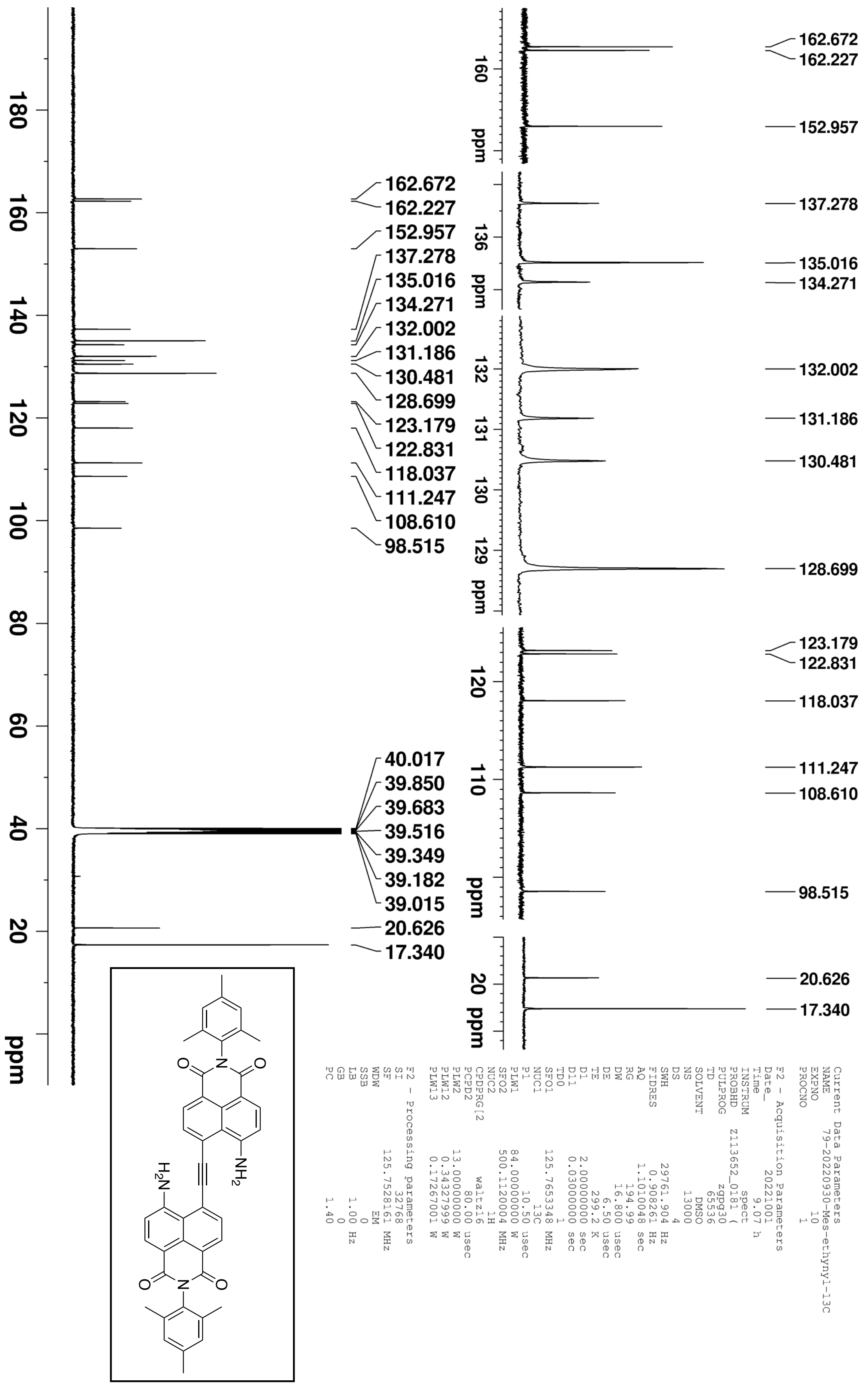

Figure S6. ^{13}C NMR spectrum of **10a** in DMSO-d_6 at 25 °C.

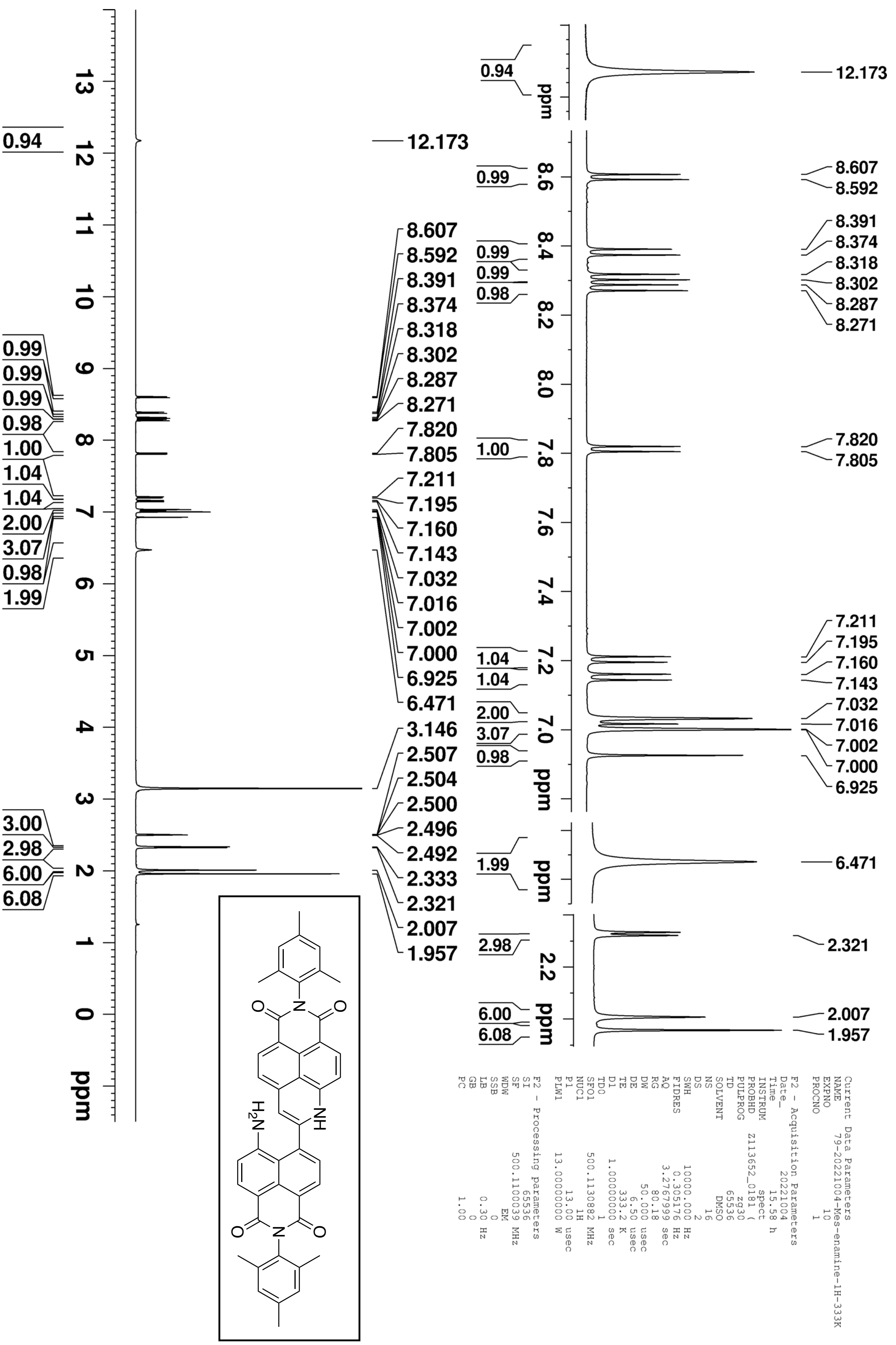

Figure S7. ^{1}H NMR spectrum of **11a** in DMSO-d_6 at 60 °C.

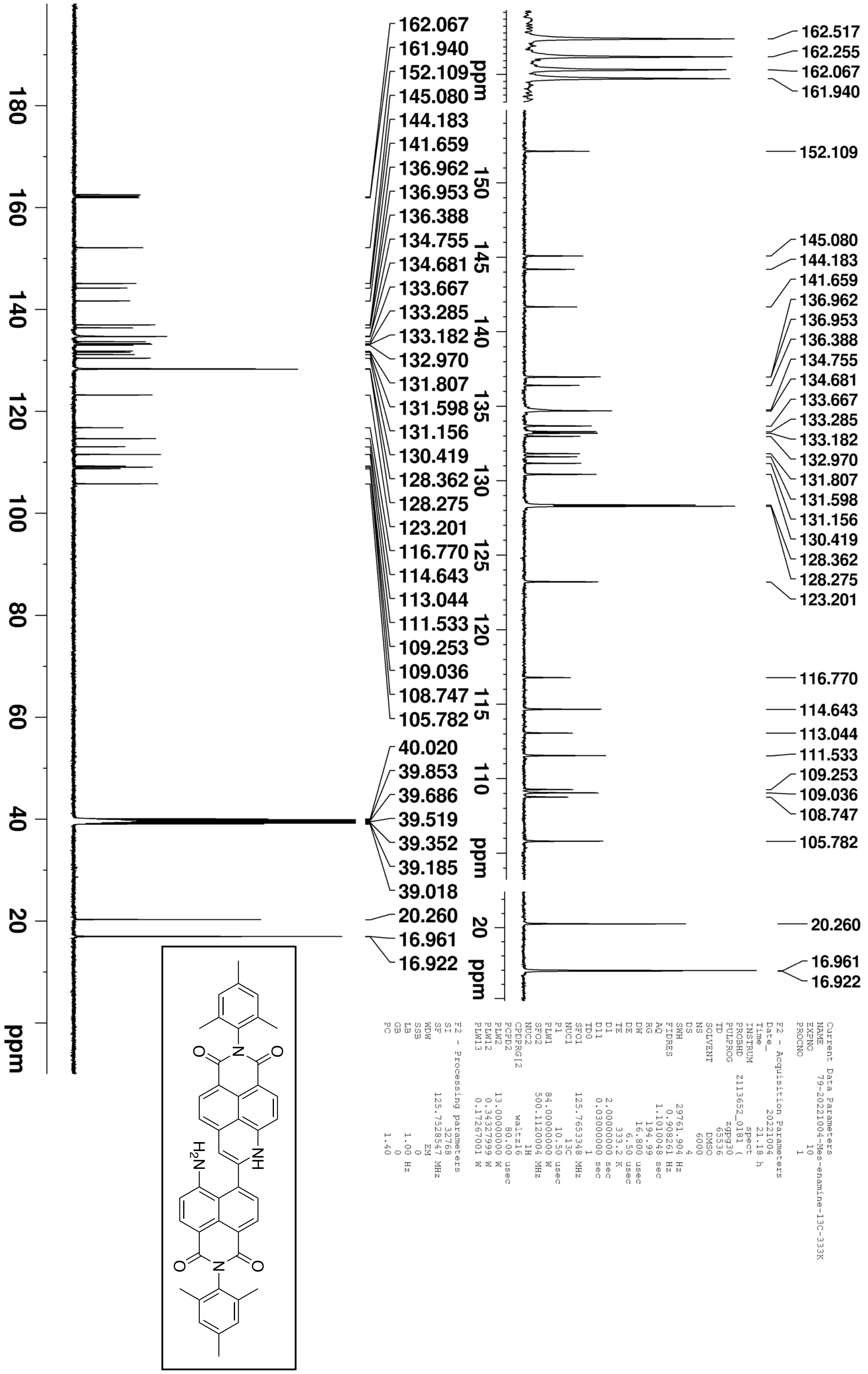

Figure S8. ^{13}C NMR spectrum of **11a** in DMSO-d_6 at 60 °C.

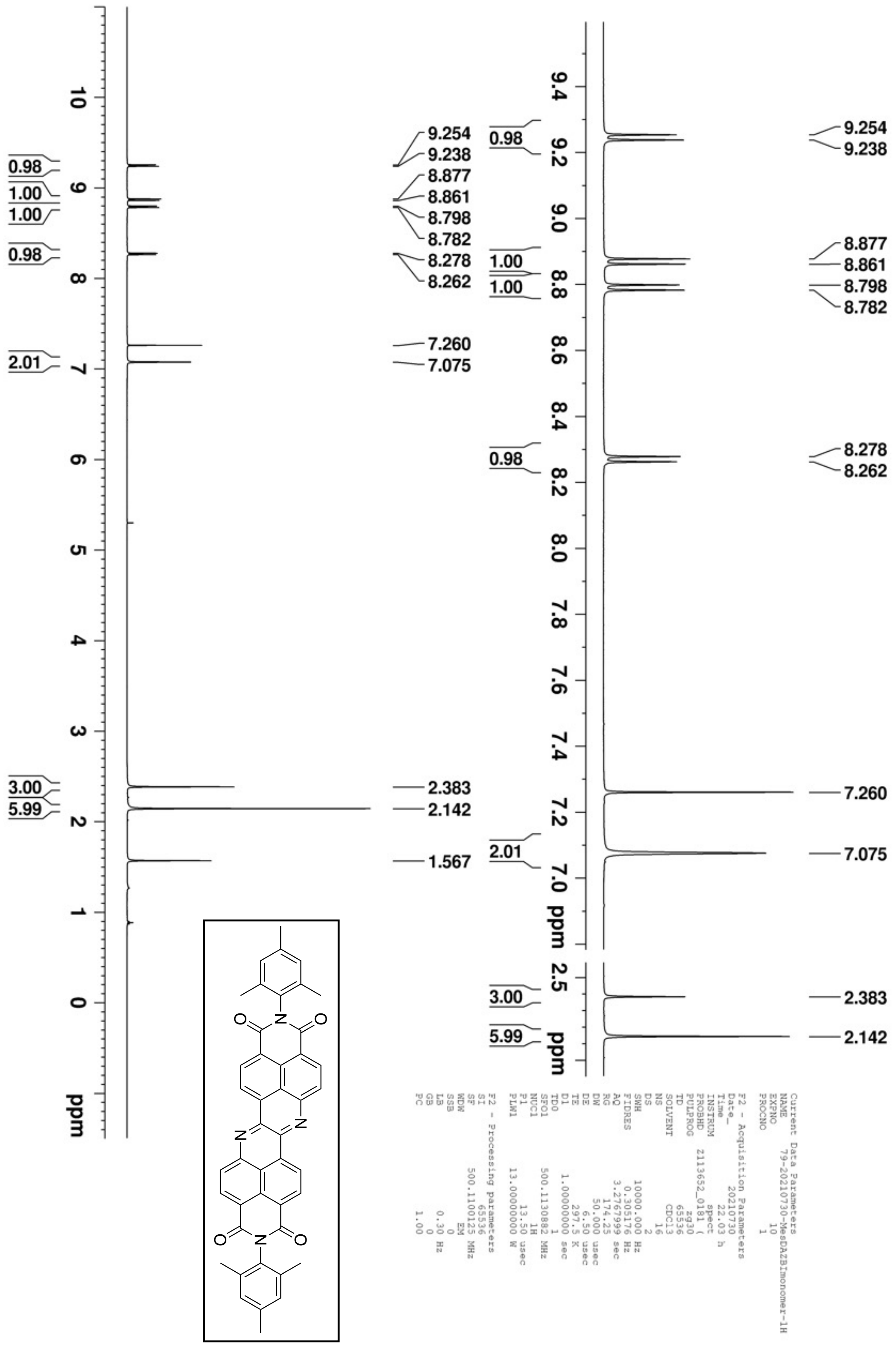

Figure S9. ^{1}H NMR spectrum of **8a** in $CDCl_3$ at 25 °C.

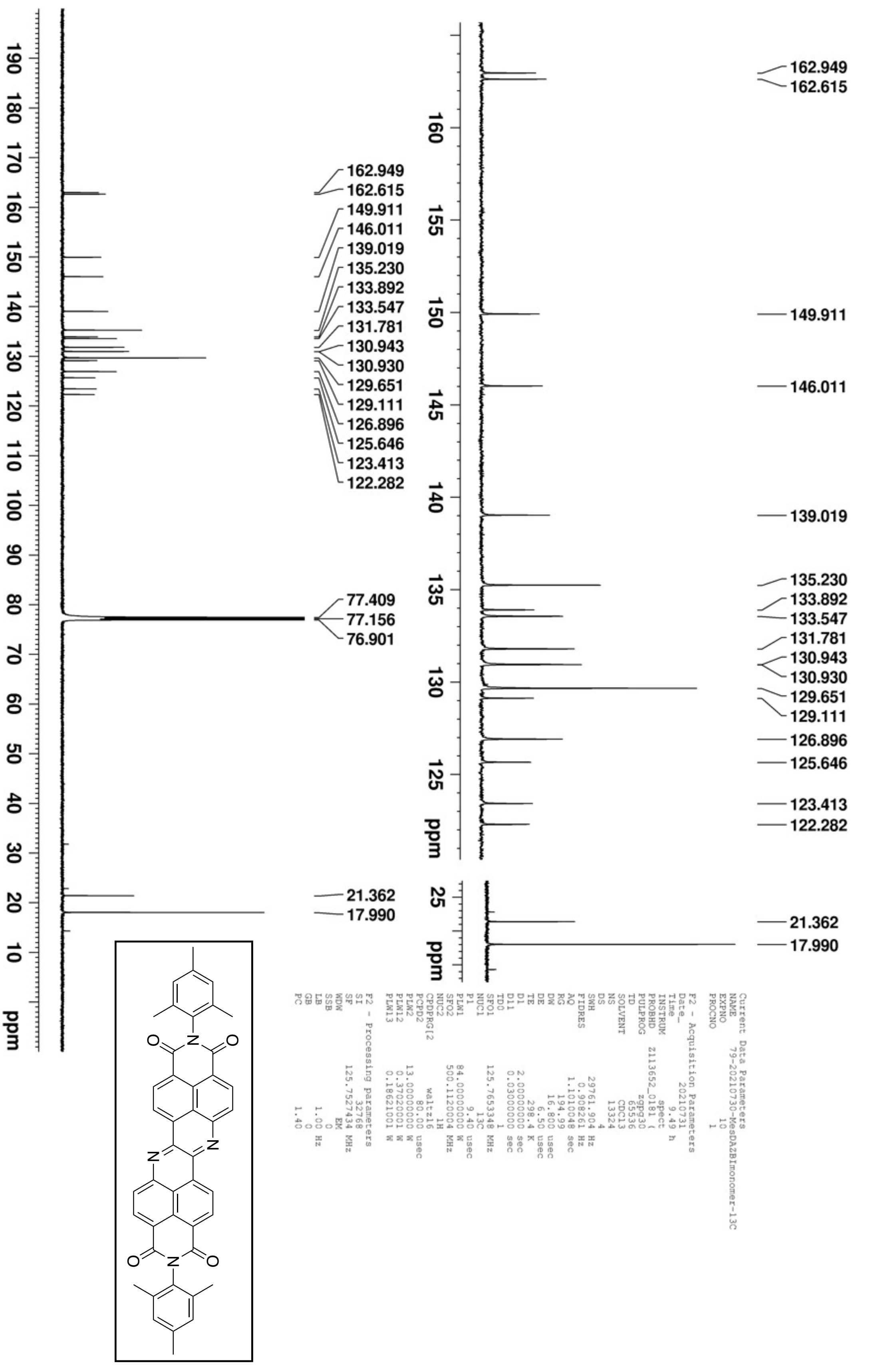

Figure S10. ^{13}C NMR spectrum of **8a** in $CDCl_3$ at 25 °C.

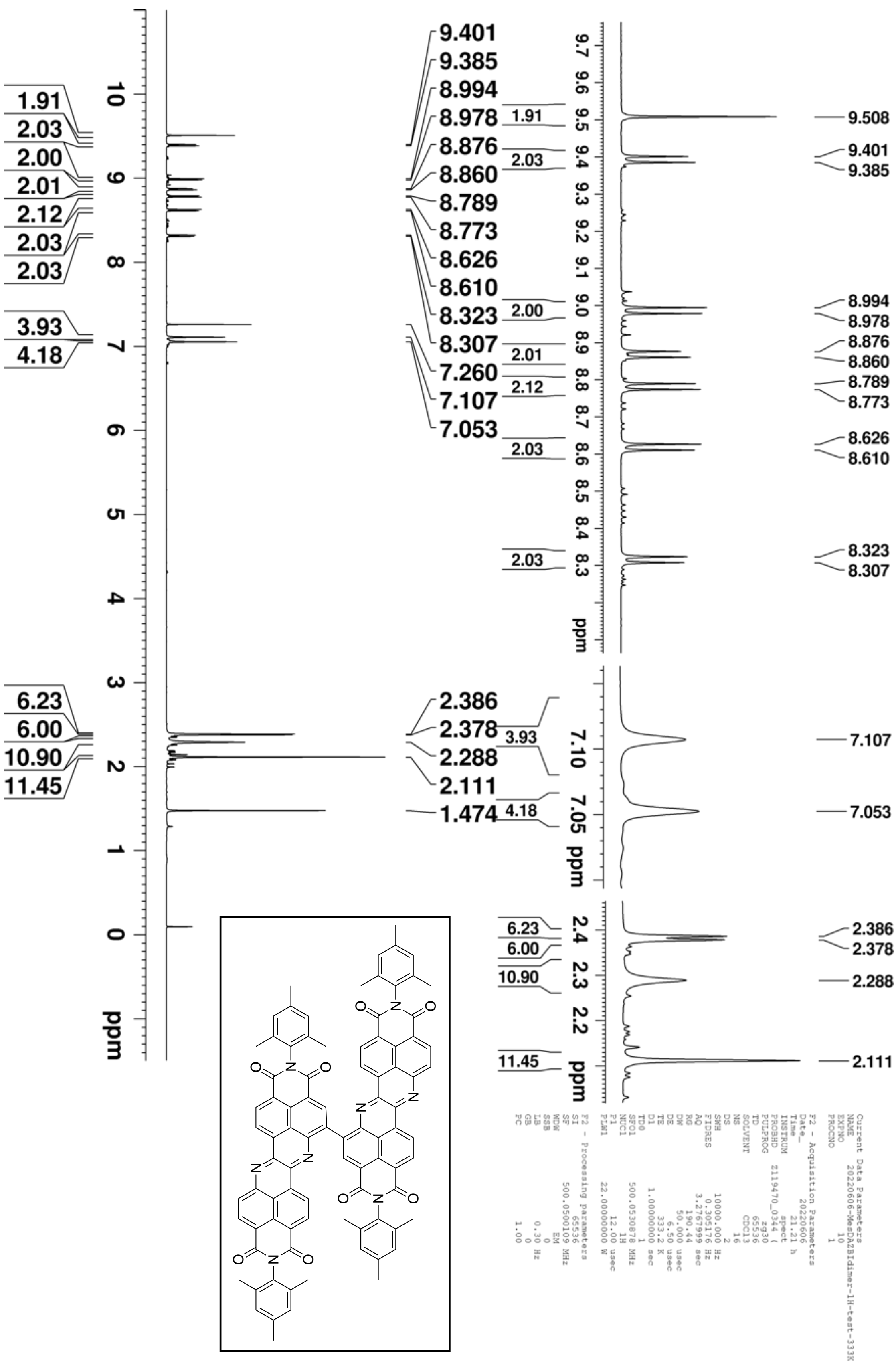

Figure S11. ^{1}H NMR spectrum of **12a** in $CDCl_3$ at 60 °C.

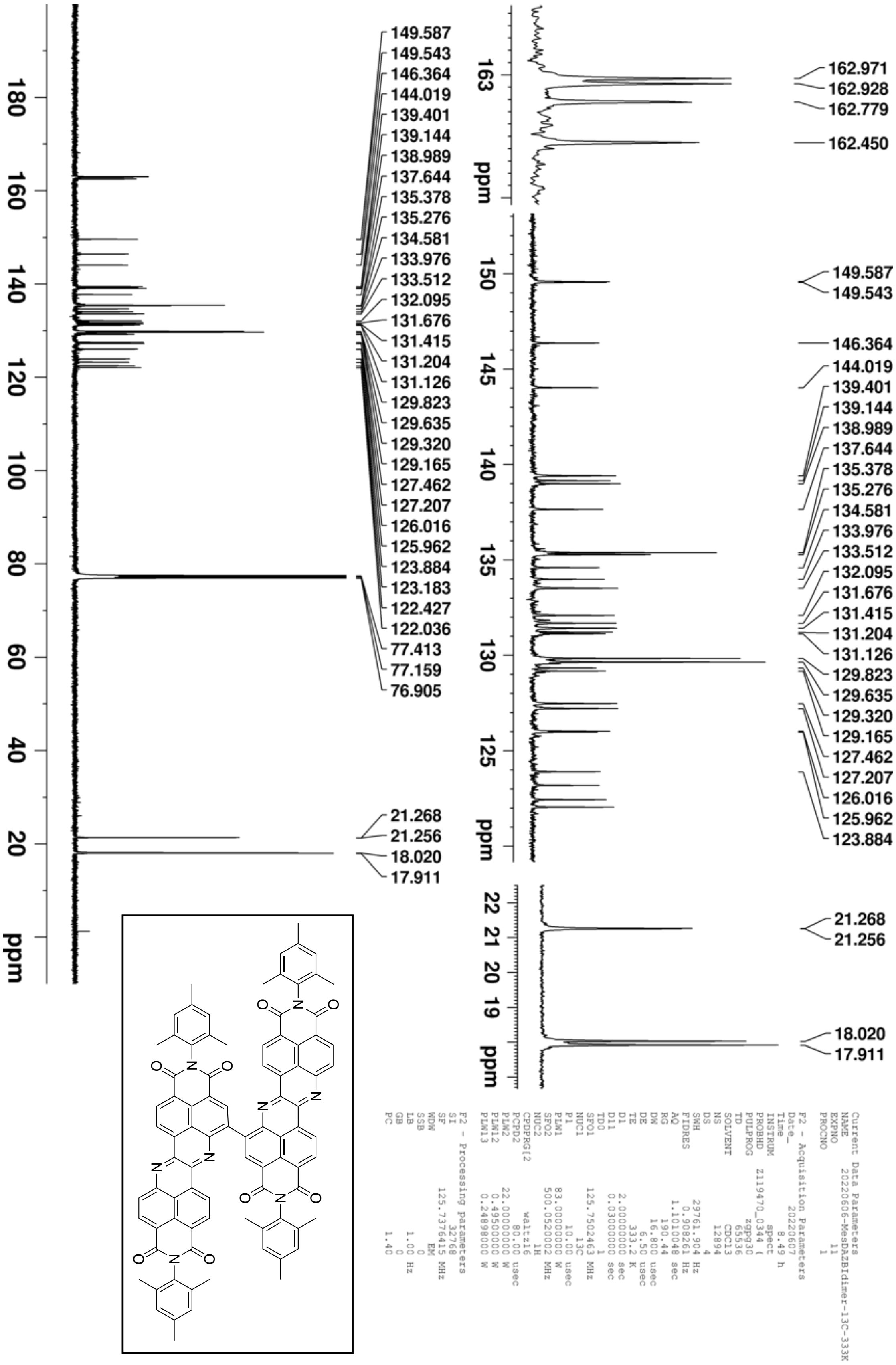

Figure S12. ^{13}C NMR spectrum of **12a** in $CDCl_3$ at 60 °C.

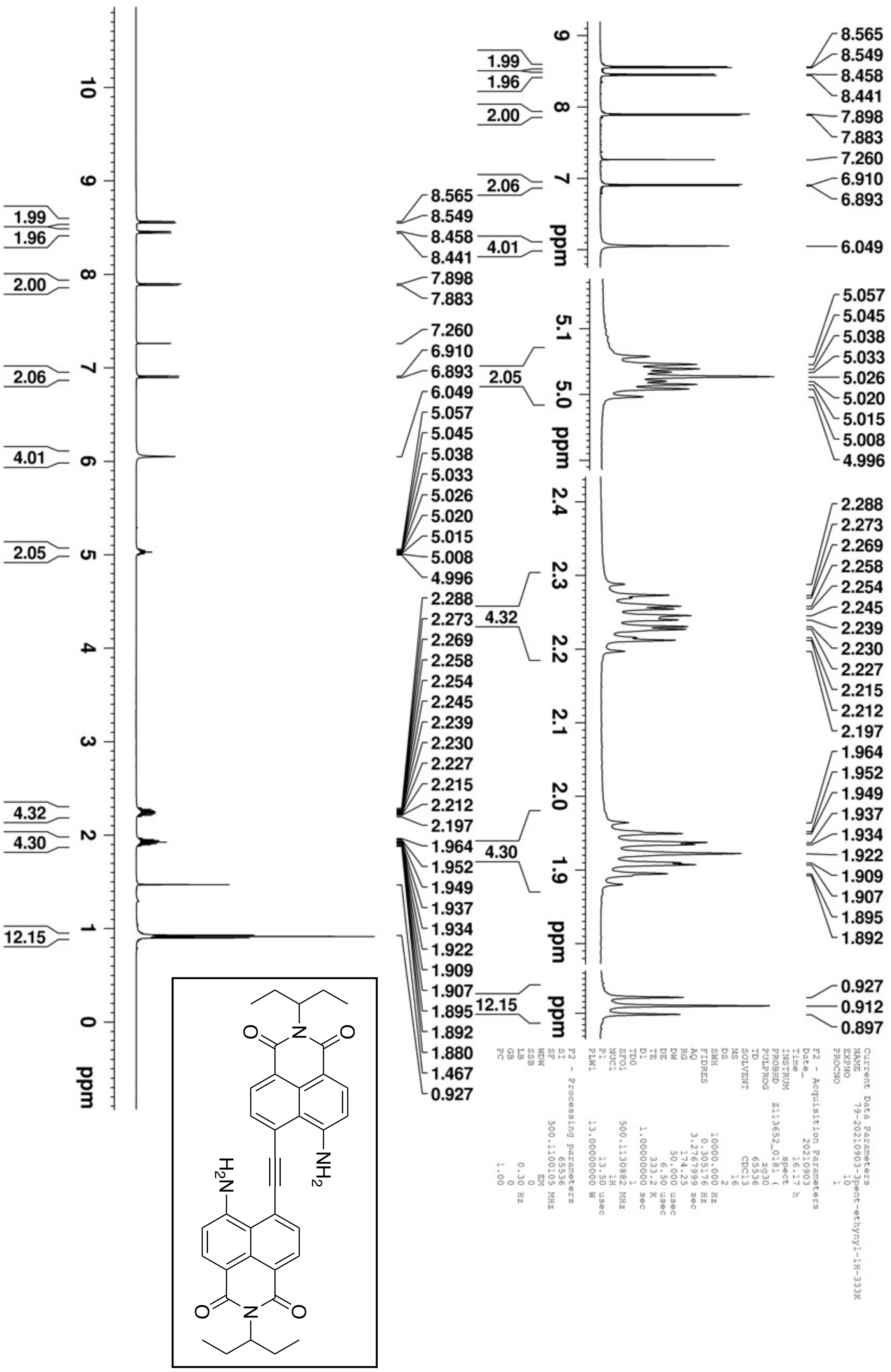

Figure S13. ^{1}H NMR spectrum of **10b** in $CDCl_3$ at 25 °C.

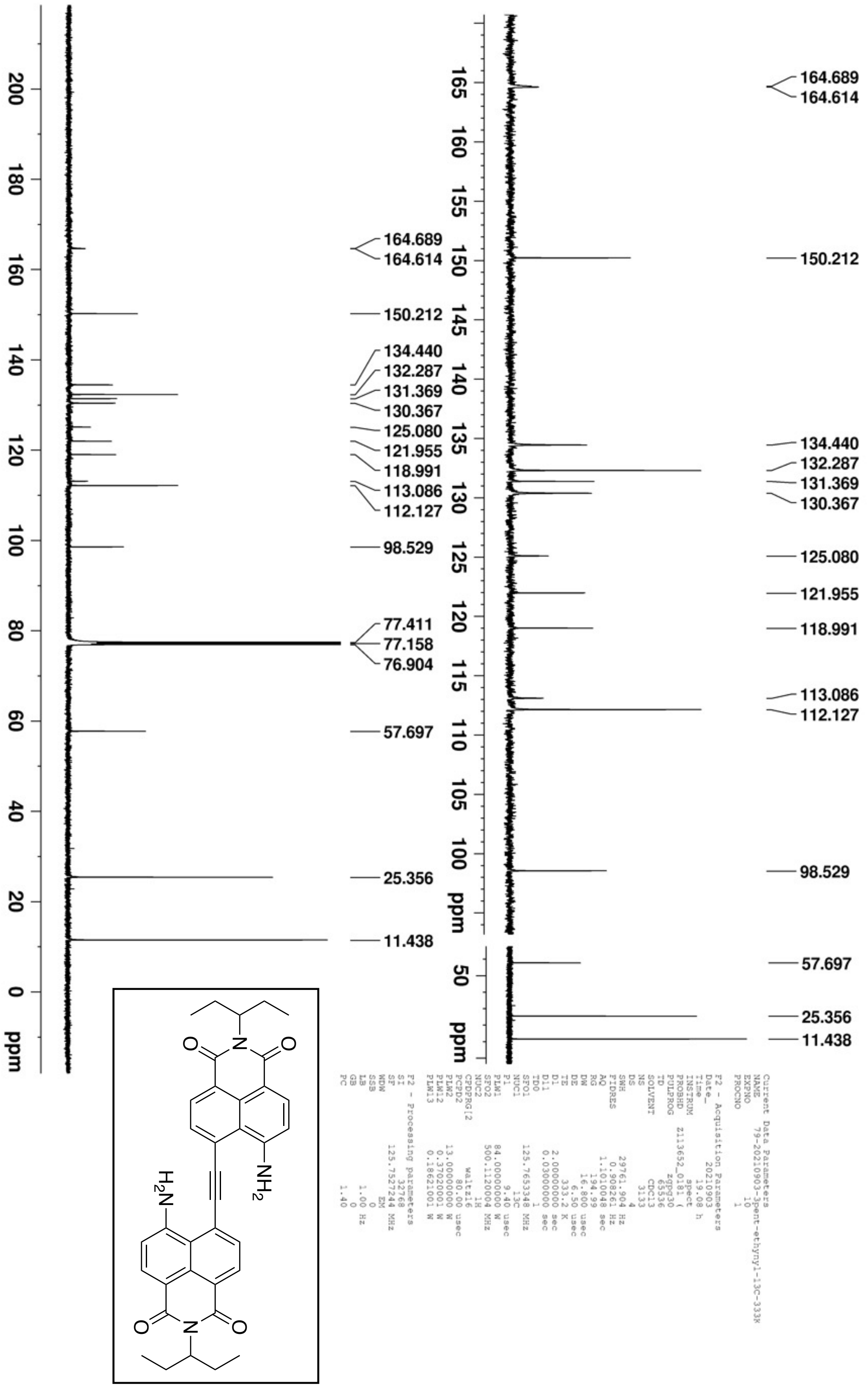

Figure S14. ^{13}C NMR spectrum of **10b** in $CDCl_3$ at 25 °C.

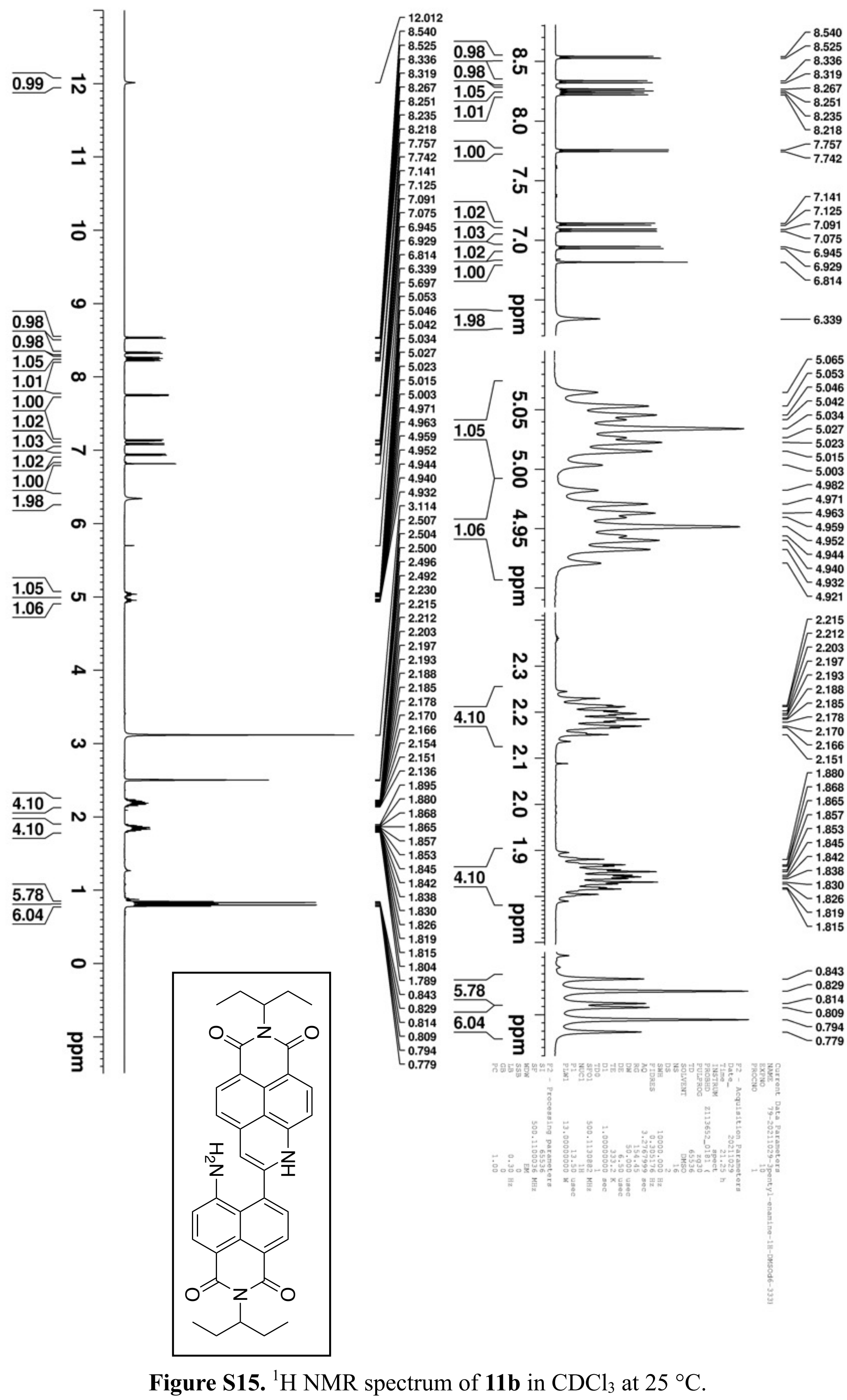

Figure S15. ^{1}H NMR spectrum of **11b** in $CDCl_3$ at 25 °C.

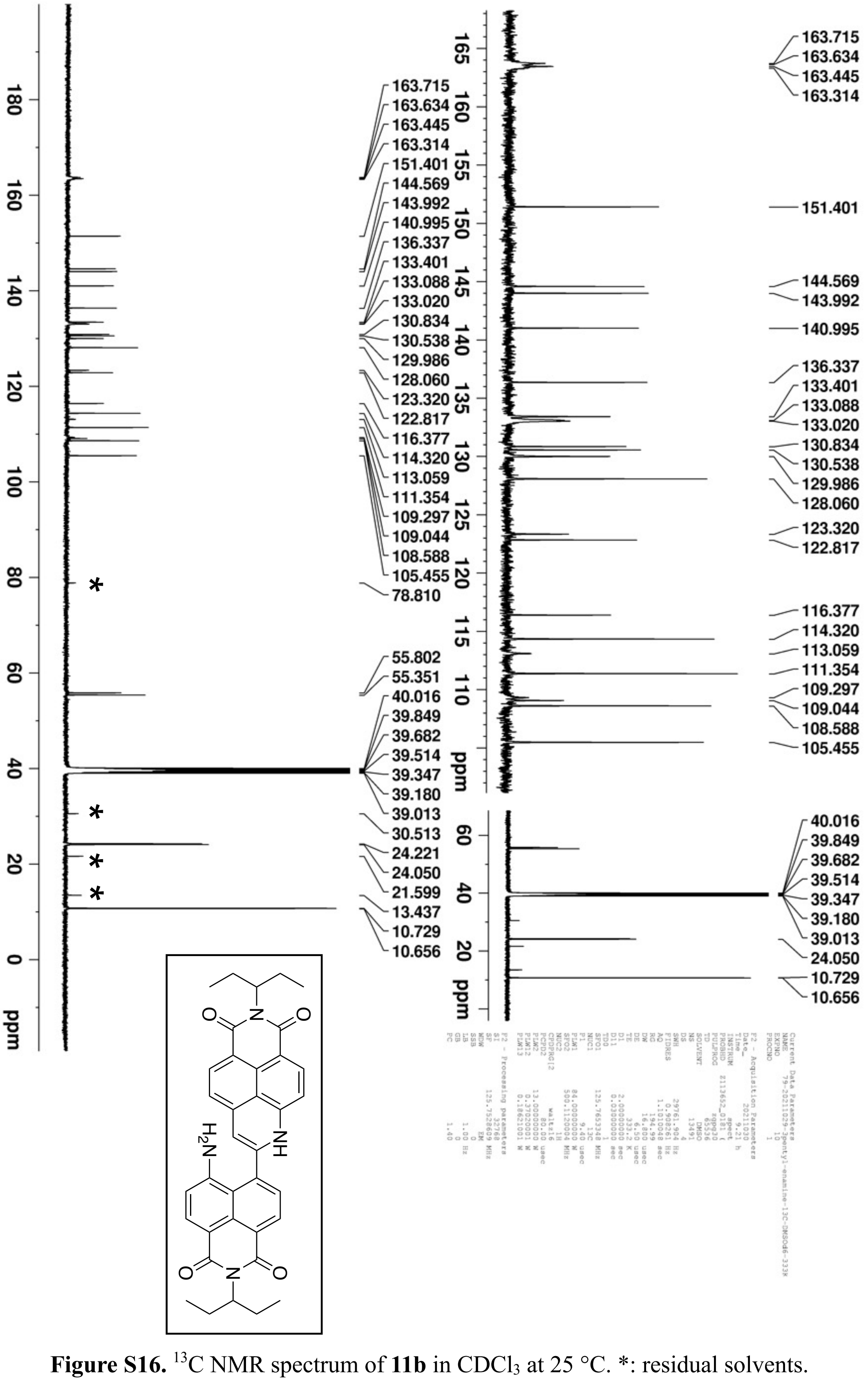

Figure S16. ^{13}C NMR spectrum of **11b** in $CDCl_3$ at 25 °C. *: residual solvents.

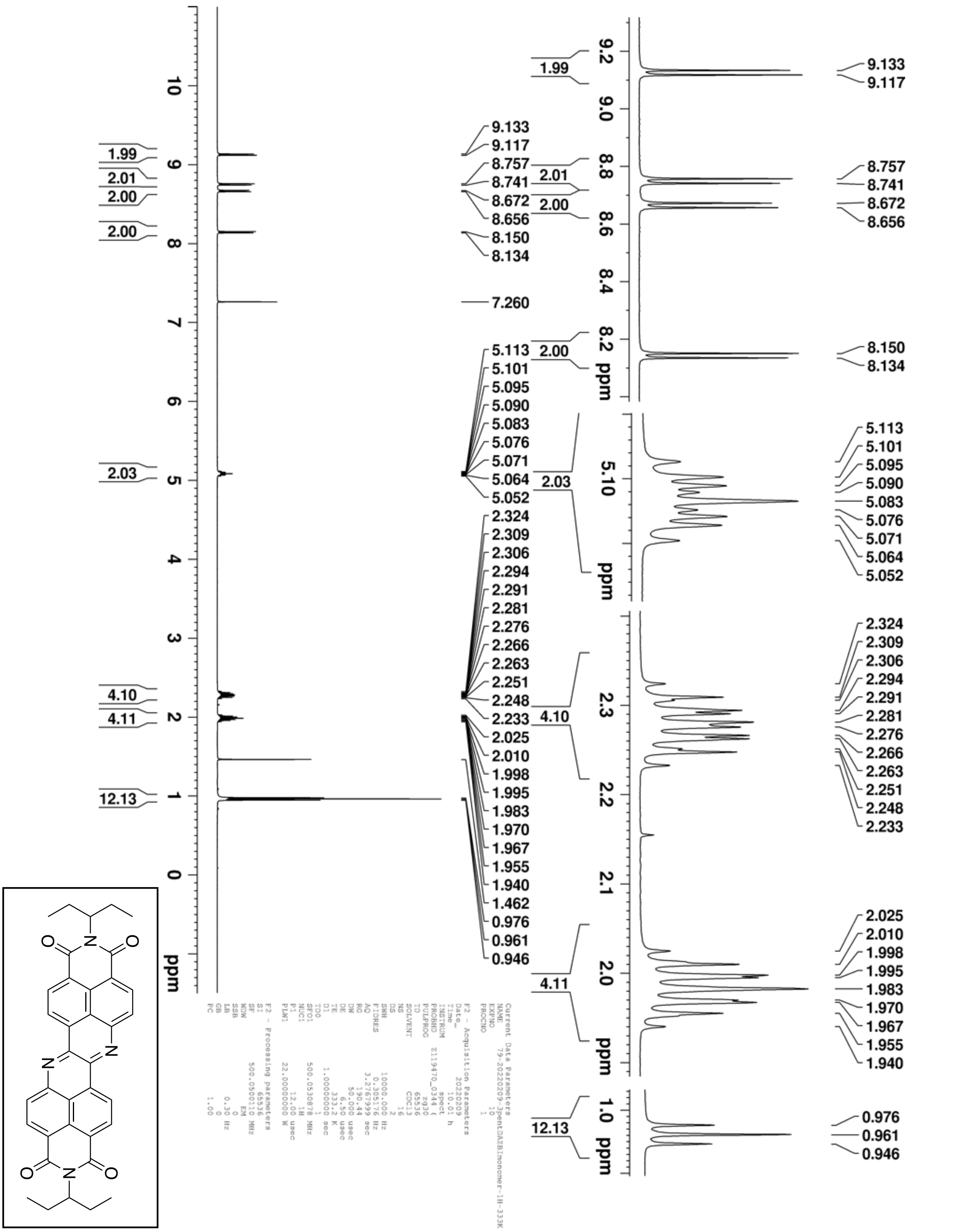

Figure S17. ^{1}H NMR spectrum of **8b** in $CDCl_3$ at 25 °C.

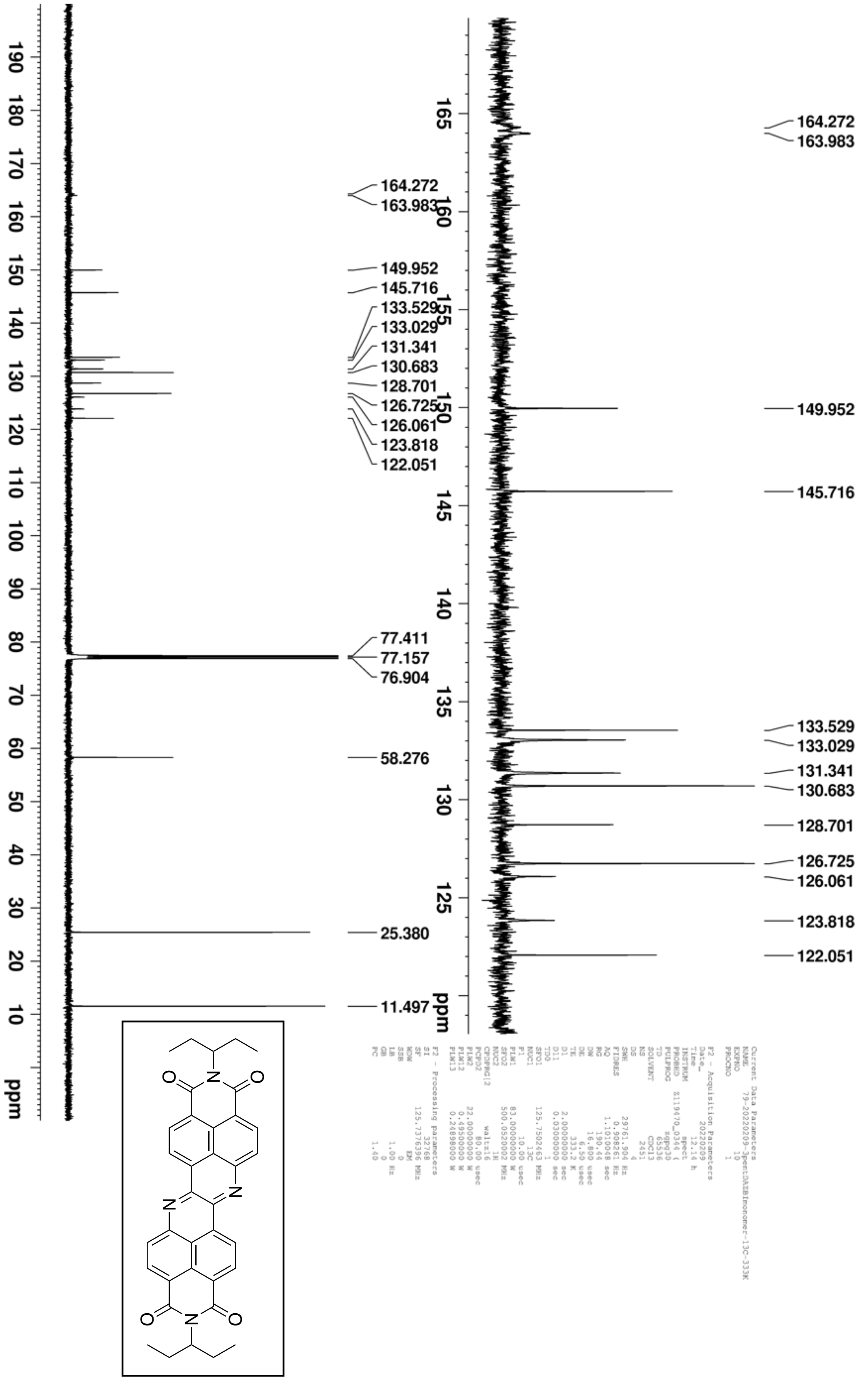

Figure S18. ^{13}C NMR spectrum of **8b** in $CDCl_3$ at 25 °C.

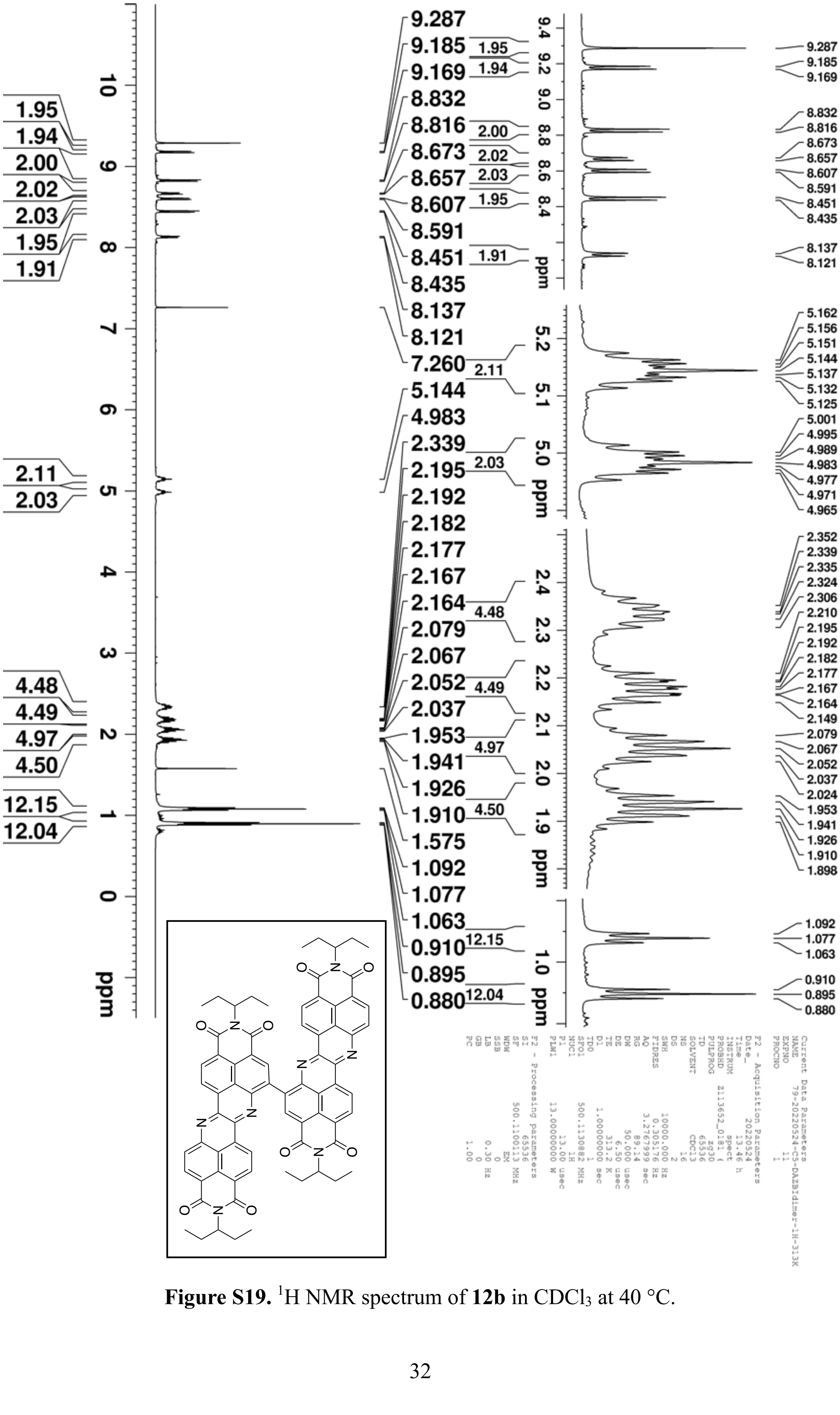

Figure S19. ^{1}H NMR spectrum of **12b** in $CDCl_3$ at 40 °C.

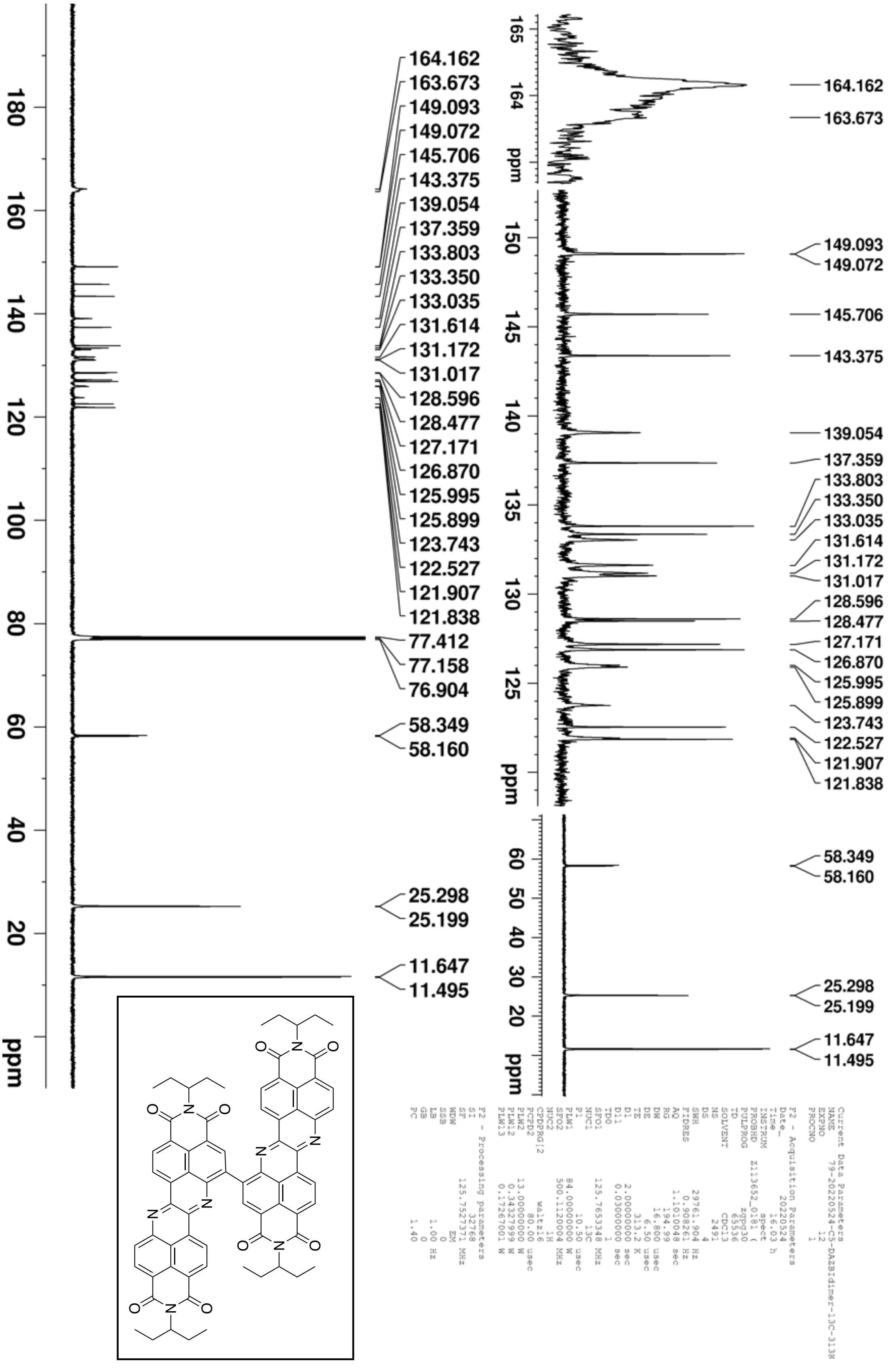

Figure S20. ^{13}C NMR spectrum of **12b** in $CDCl_3$ at 40 °C.

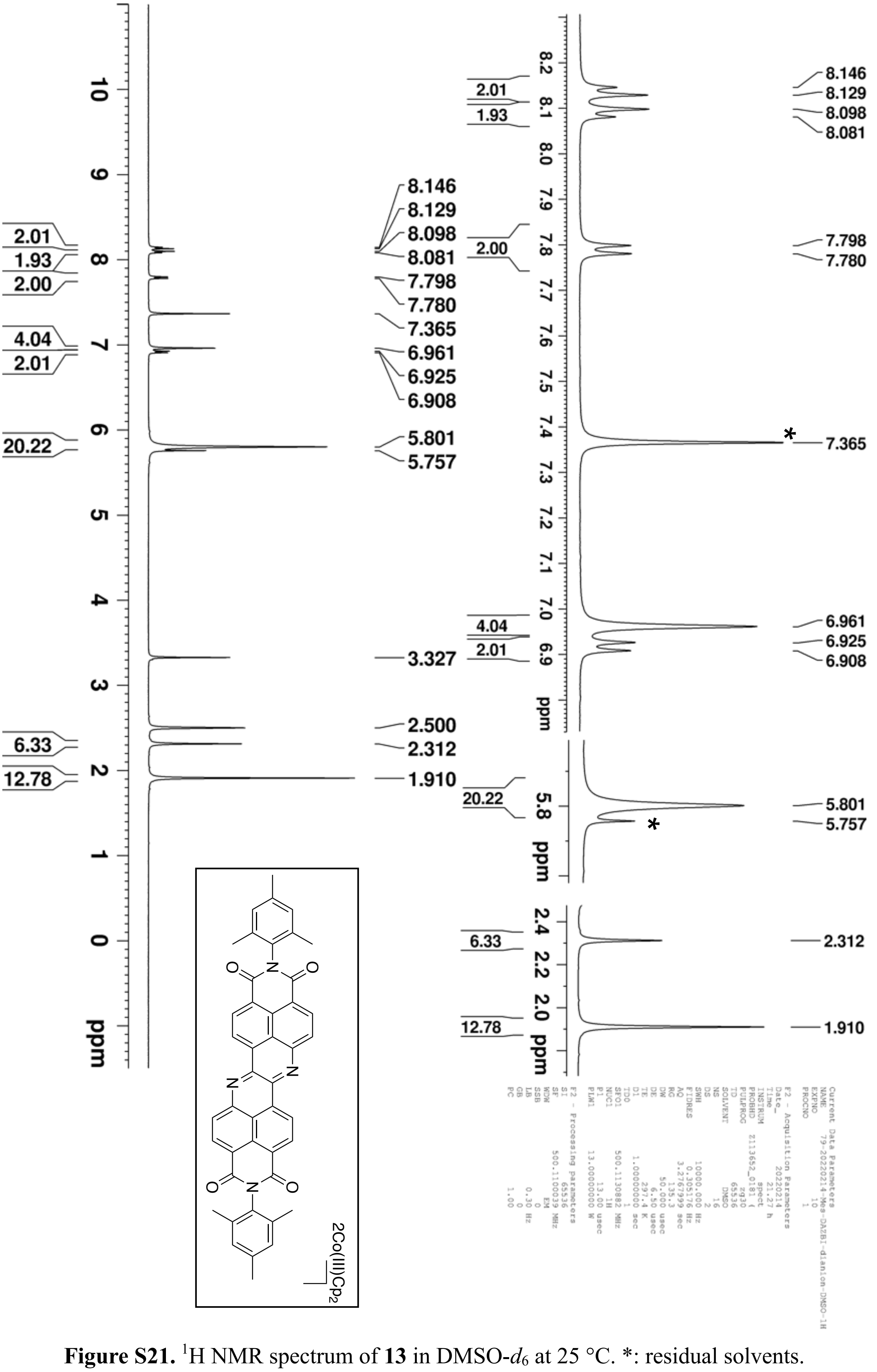

Figure S21. ^{1}H NMR spectrum of **13** in DMSO-d_6 at 25 °C. *: residual solvents.

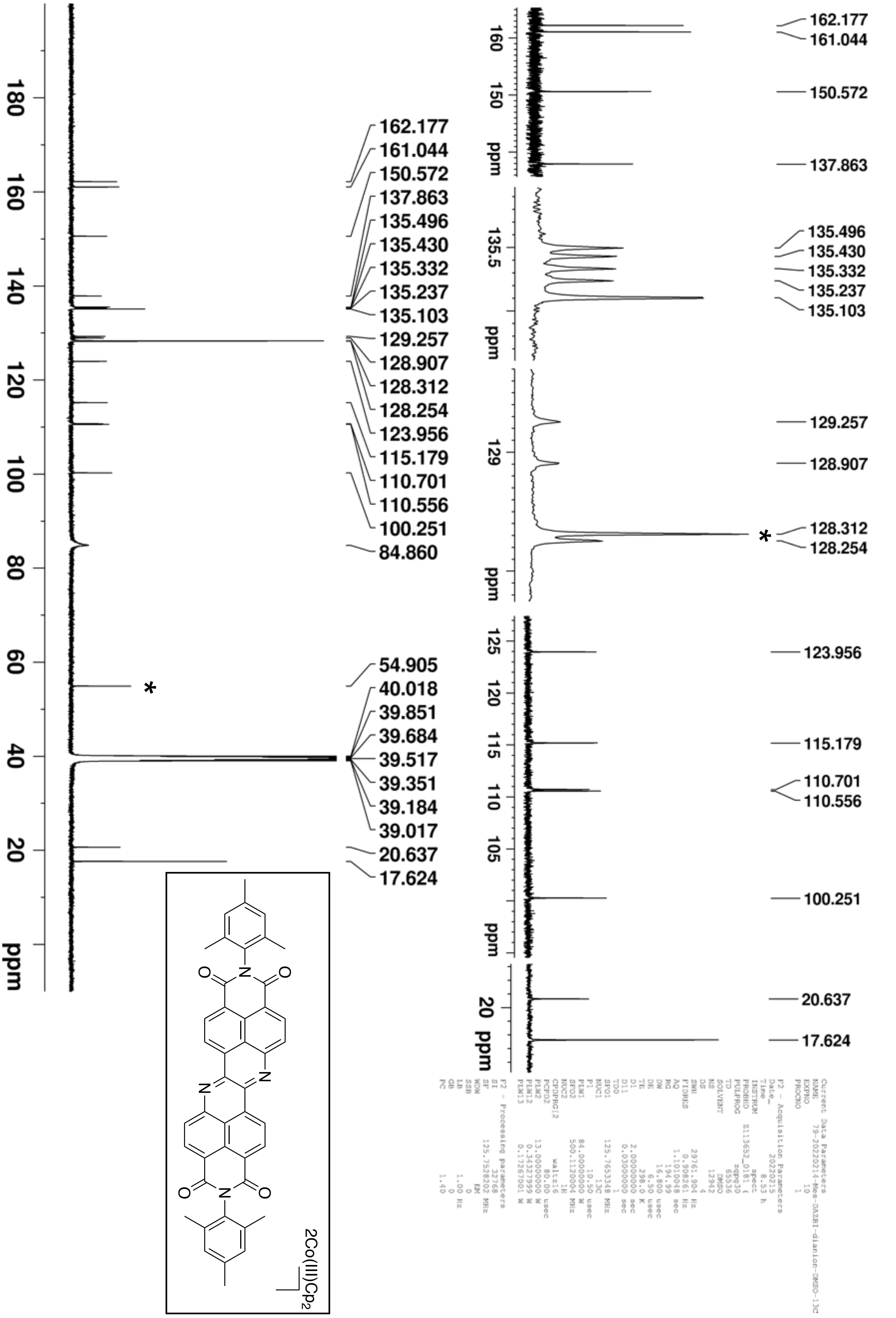

Figure S22. ^{13}C NMR spectrum of **13** in DMSO-d_6 at 25 °C. *: residual solvents.

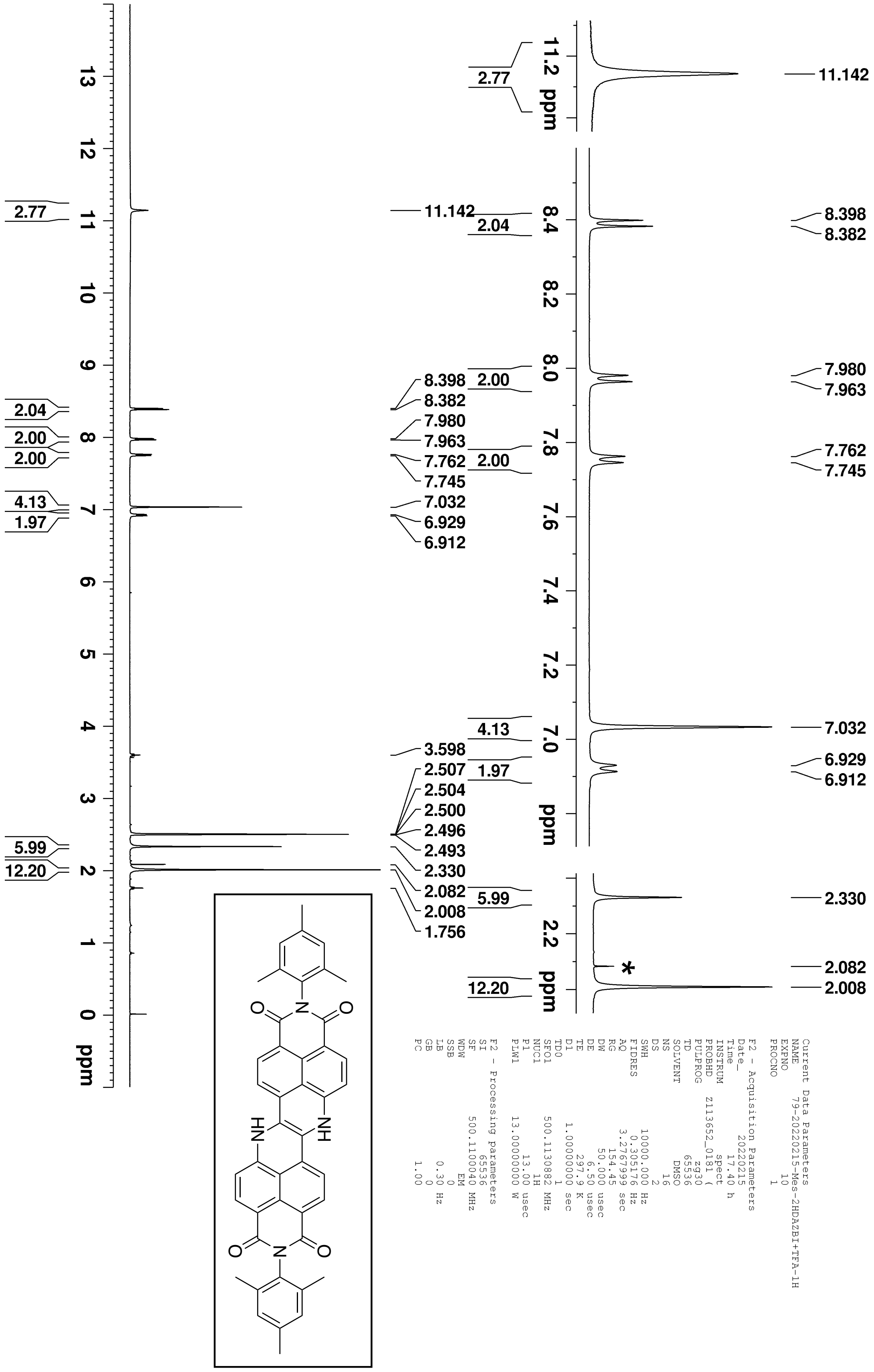

Figure S23. ¹H NMR spectrum of **14a** in DMSO-d_6 + CF_3COOH at 25 °C. *: residual solvents.

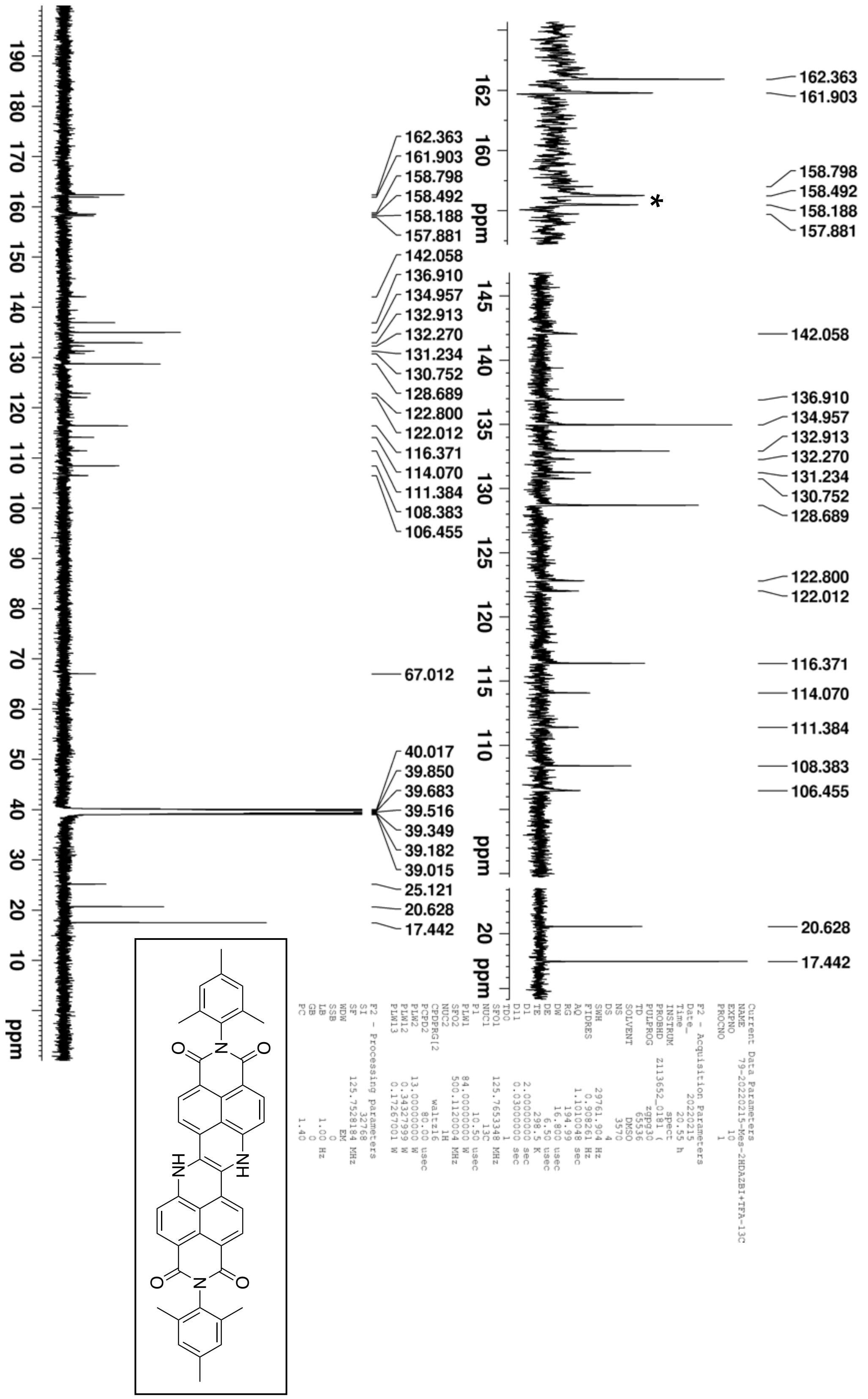

Figure S24. ^{13}C NMR spectrum of **14a** in DMSO-d_6 + CF_3COOH at 25 °C.

*: CF_3COOH signals.

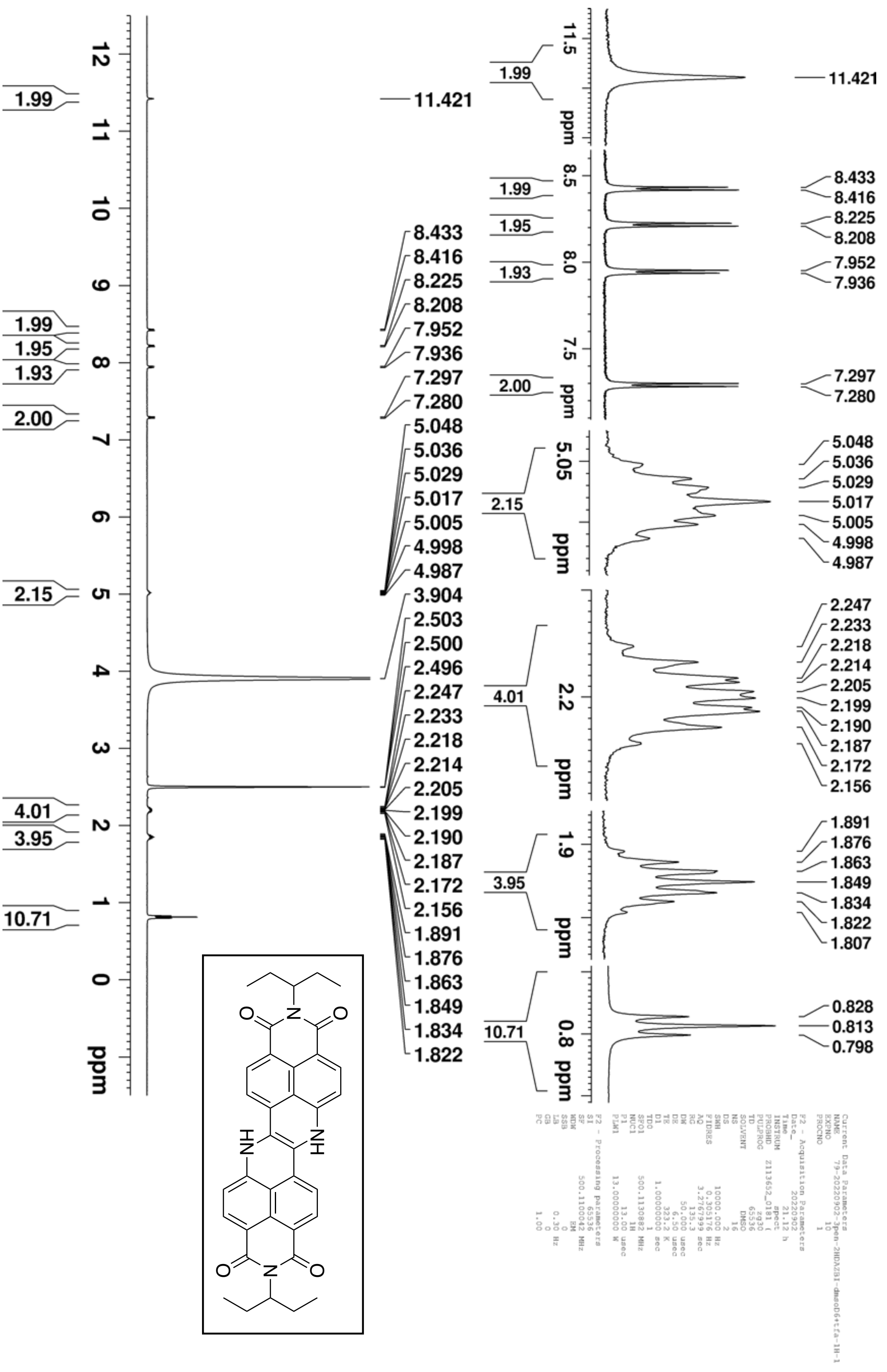

Figure S25. ^{1}H NMR spectrum of **14b** in DMSO-d_6 + CF_3COOH at 50 °C.

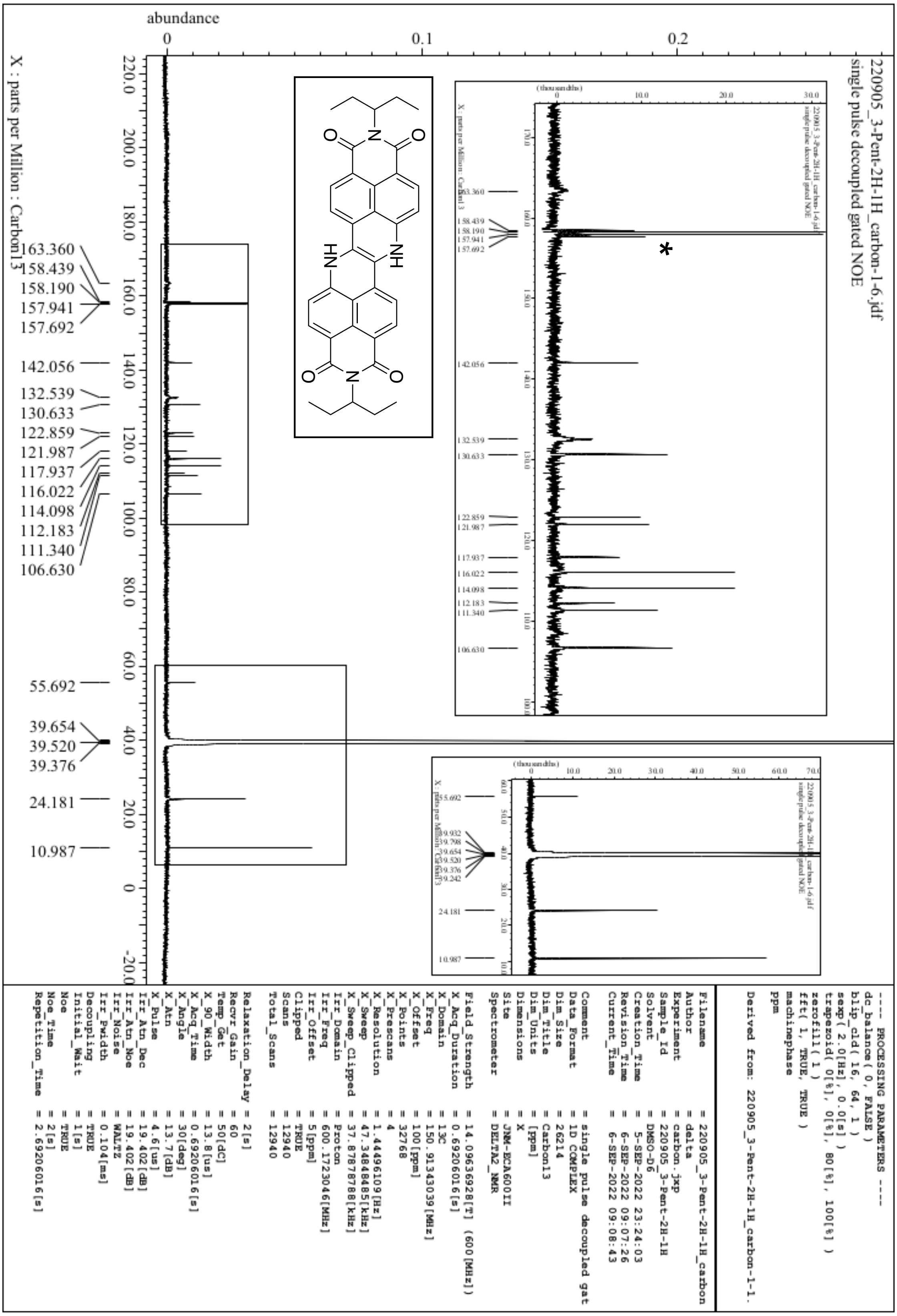

Figure S26. ^{13}C NMR spectrum of **14b** in DMSO-d_6 + CF_3COOH at 50 °C.

*: CF_3COOH signals.

4. Mass spectra

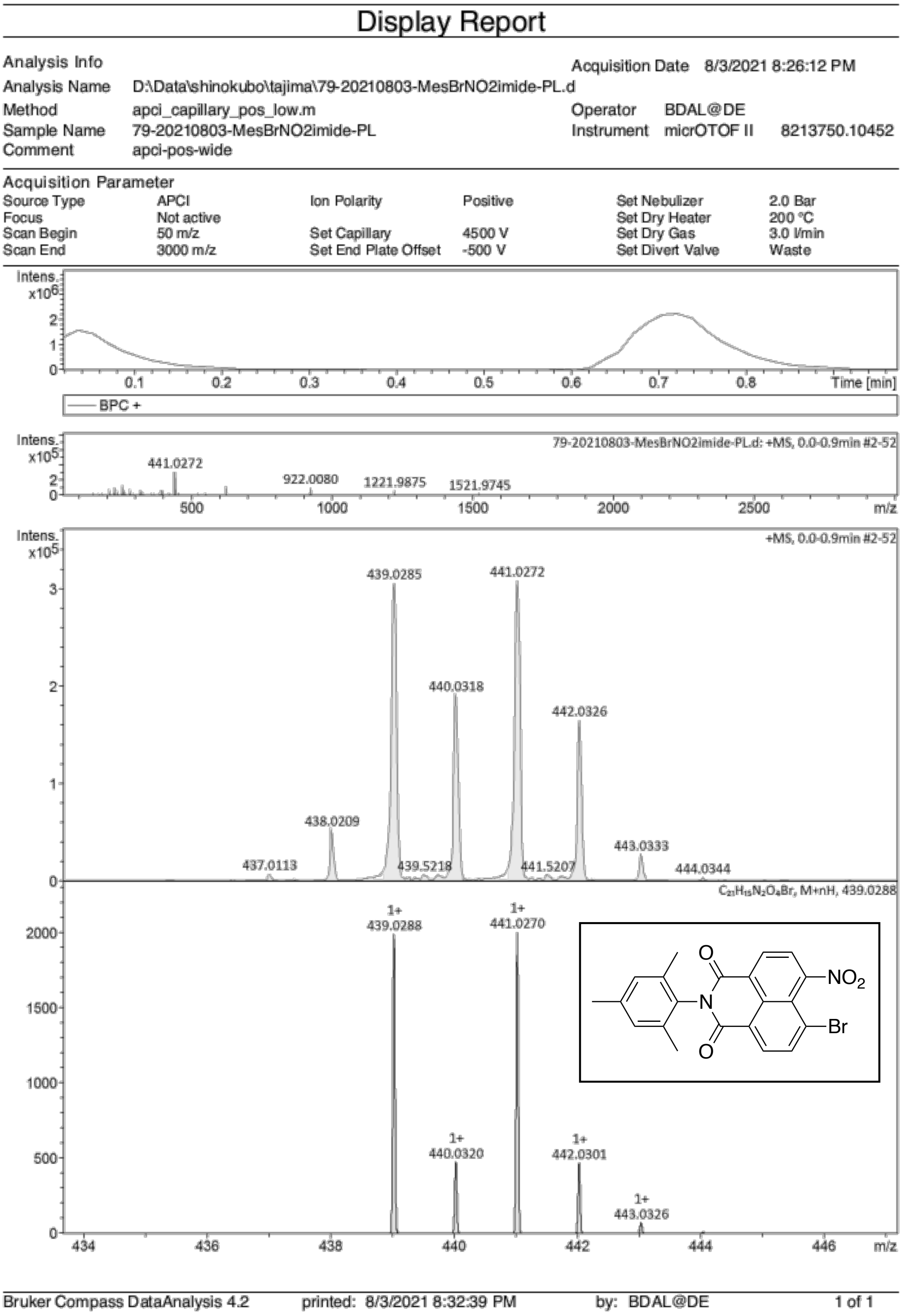

Figure S27. APCI-TOF (positive mode) mass spectrum of **S1**.

Display Report

Analysis Info

Analysis Name	D:\Data\shinokubo\tajima\79-20210917-Mesimide-NH2Br-PL-2.d	Acquisition Date	9/17/2021 2:41:53 PM
Method	apci_capillary_pos_low.m	Operator	BDAL@DE
Sample Name	79-20210917-Mesimide-NH2Br-PL-2	Instrument	micrOTOF II 8213750.10452
Comment	apciposwide		

Acquisition Parameter

Source Type	APCI	Ion Polarity	Positive	Set Nebulizer	2.0 Bar
Focus	Not active			Set Dry Heater	200 °C
Scan Begin	50 m/z	Set Capillary	4500 V	Set Dry Gas	3.0 l/min
Scan End	3000 m/z	Set End Plate Offset	-500 V	Set Divert Valve	Waste

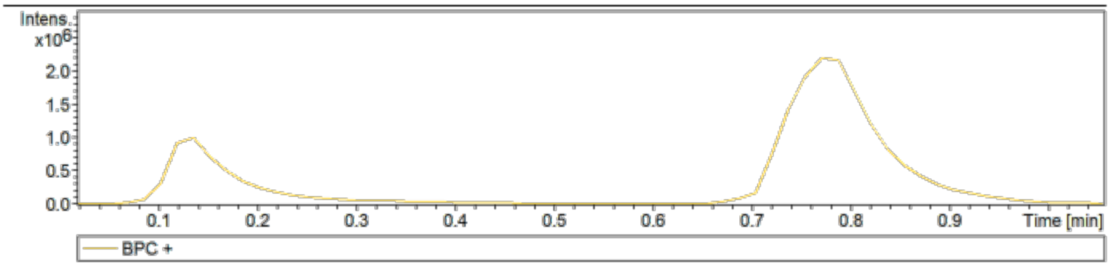

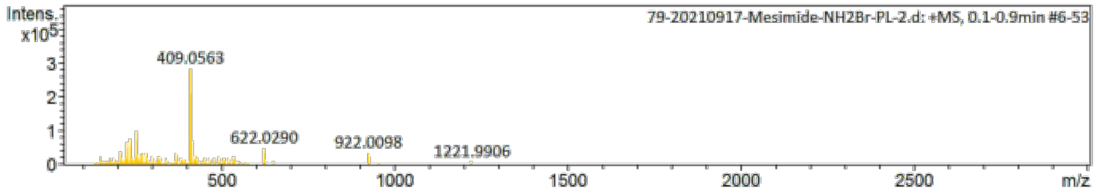

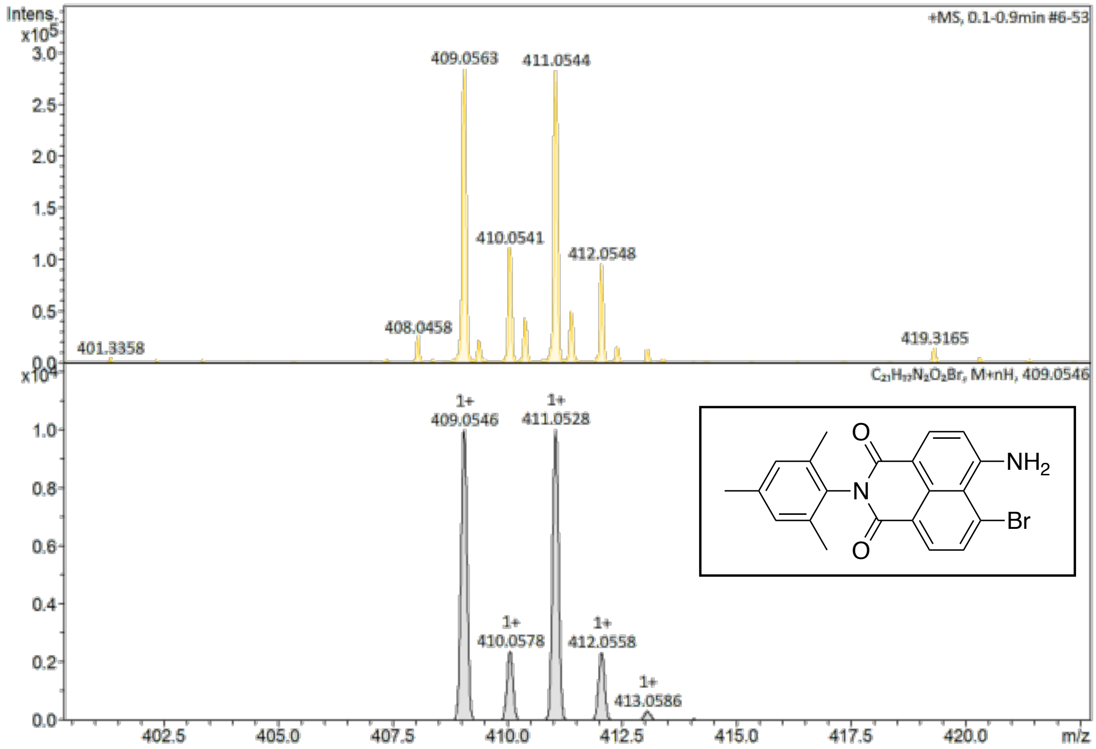

Figure S28. APCI-TOF (positive mode) mass spectrum of **9a**.

D:\Data\Shinokubo\tajima\79-20220217-Mes-ethynyldimer\Mes-ethynyldimer\0_A3\1\1Ref

Comment 1

Comment 2

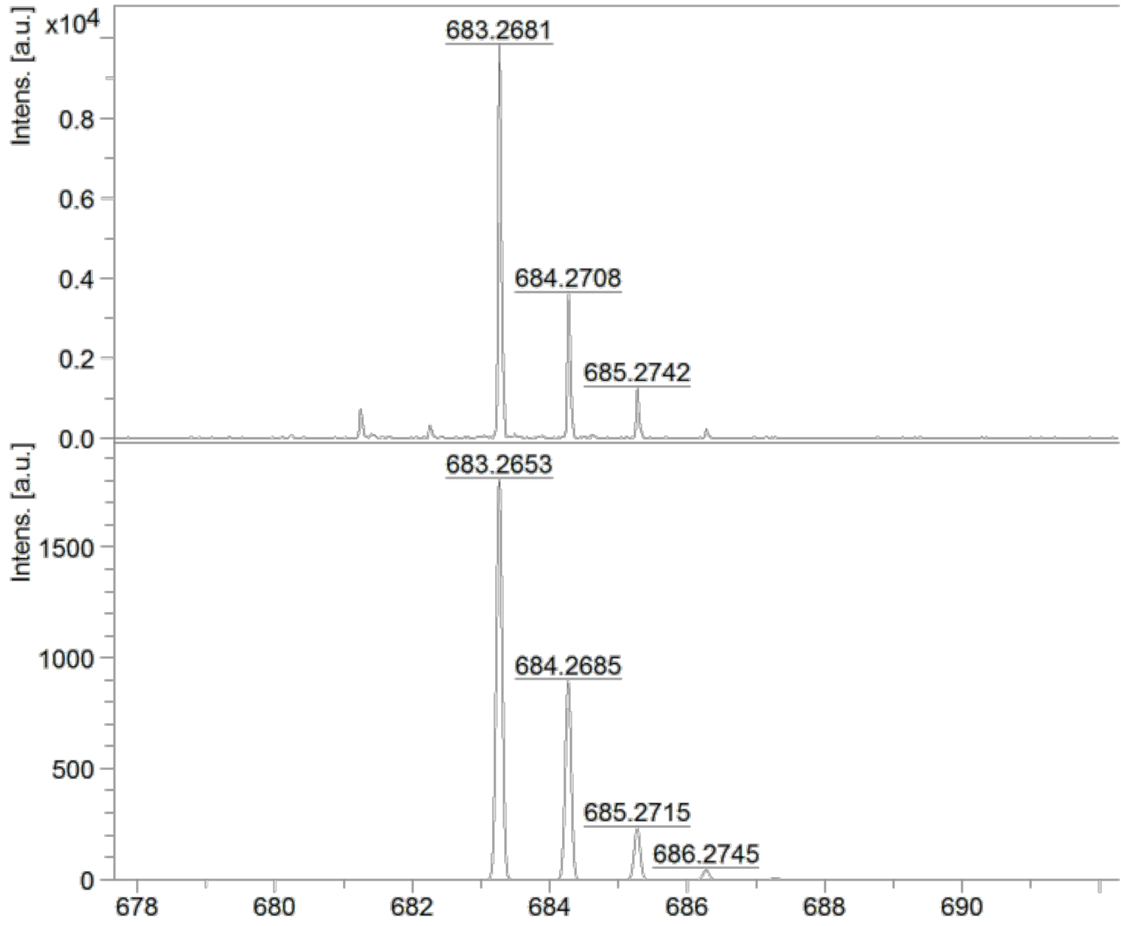

Acquisition Parameter

Date of acquisition	2022-02-17T11:34:06.444+09:00
Acquisition method name	D:\Methods\flexControlMethods\RP_0-2kDa.par
Aquisition operation mode	Reflector
Voltage polarity	POS
Number of shots	500
Name of spectrum used for calibration	
Calibration reference list used	APCI-L tuning mix

Instrument Info

User	tof-user
Instrument	FLEX-PC
Instrument type	autoflexTOF/TOF

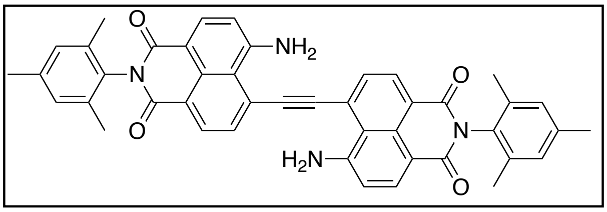

Bruker Daltonics flexAnalysis printed: 2/17/2022 11:38:31 AM

Figure S29. MALDI-TOF (positive mode) mass spectrum of **10a**.

Display Report

Analysis Info

Analysis Name	D:\Data\shinokubo\tajima\79-220106-Mes-enamine-ESI-NW-2.d	Acquisition Date	1/6/2022 5:09:00 PM
Method	esi_neg_wide_avg.m	Operator	BDAL@DE
Sample Name	79-220106-Mes-enamine-ESI-NW-2	Instrument	micrOTOF II 8213750.10452
Comment	poswide		

Acquisition Parameter

Source Type	ESI	Ion Polarity	Negative	Set Nebulizer	0.3 Bar
Focus	Not active			Set Dry Heater	200 °C
Scan Begin	50 m/z	Set Capillary	2900 V	Set Dry Gas	4.0 l/min
Scan End	3000 m/z	Set End Plate Offset	-500 V	Set Divert Valve	Waste

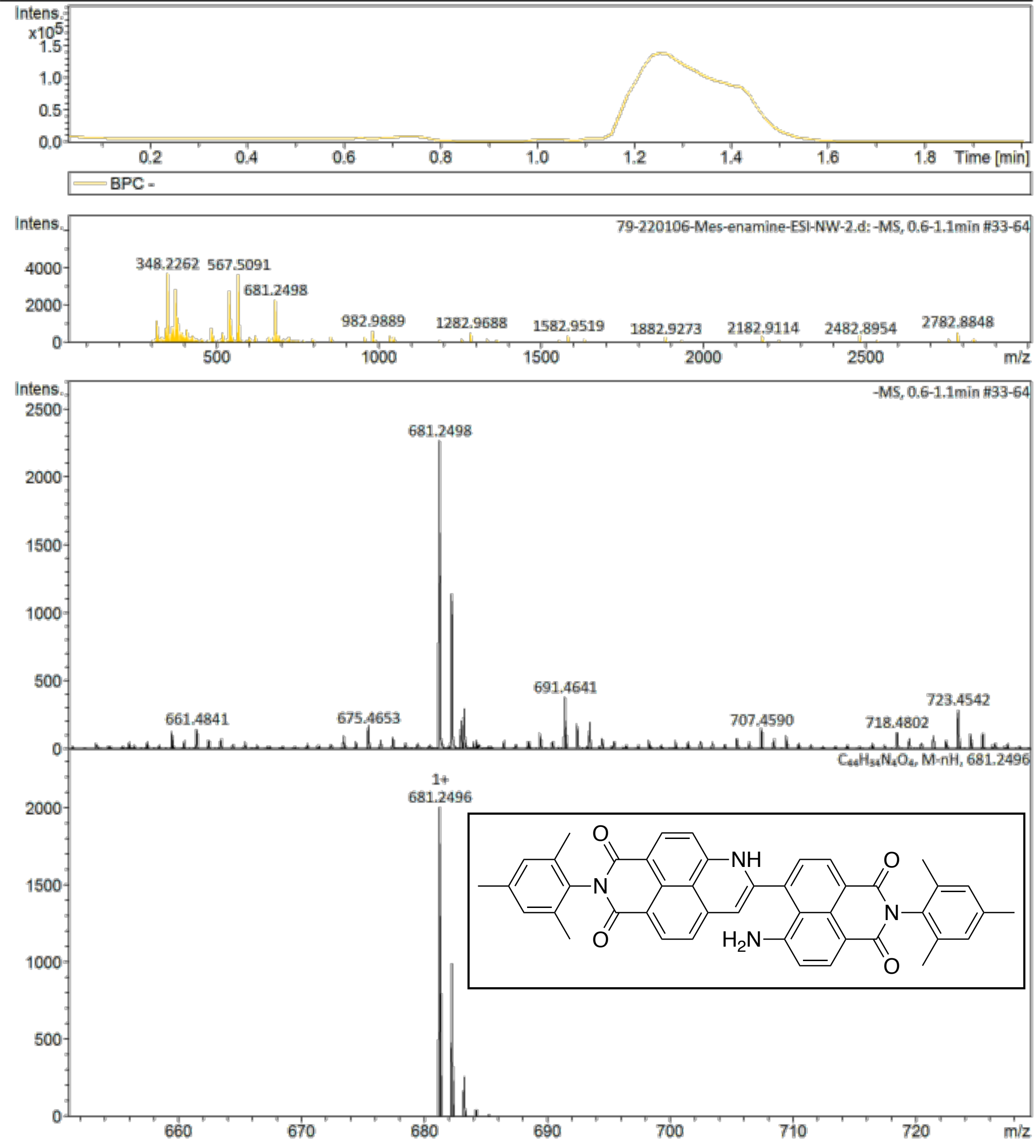

Bruker Compass DataAnalysis 4.2 printed: 1/6/2022 6:06:07 PM by: BDAL@DE 1 of 1

Figure S30. ESI-TOF (negative mode) mass spectrum of **11a**.

Comment 1
Comment 2

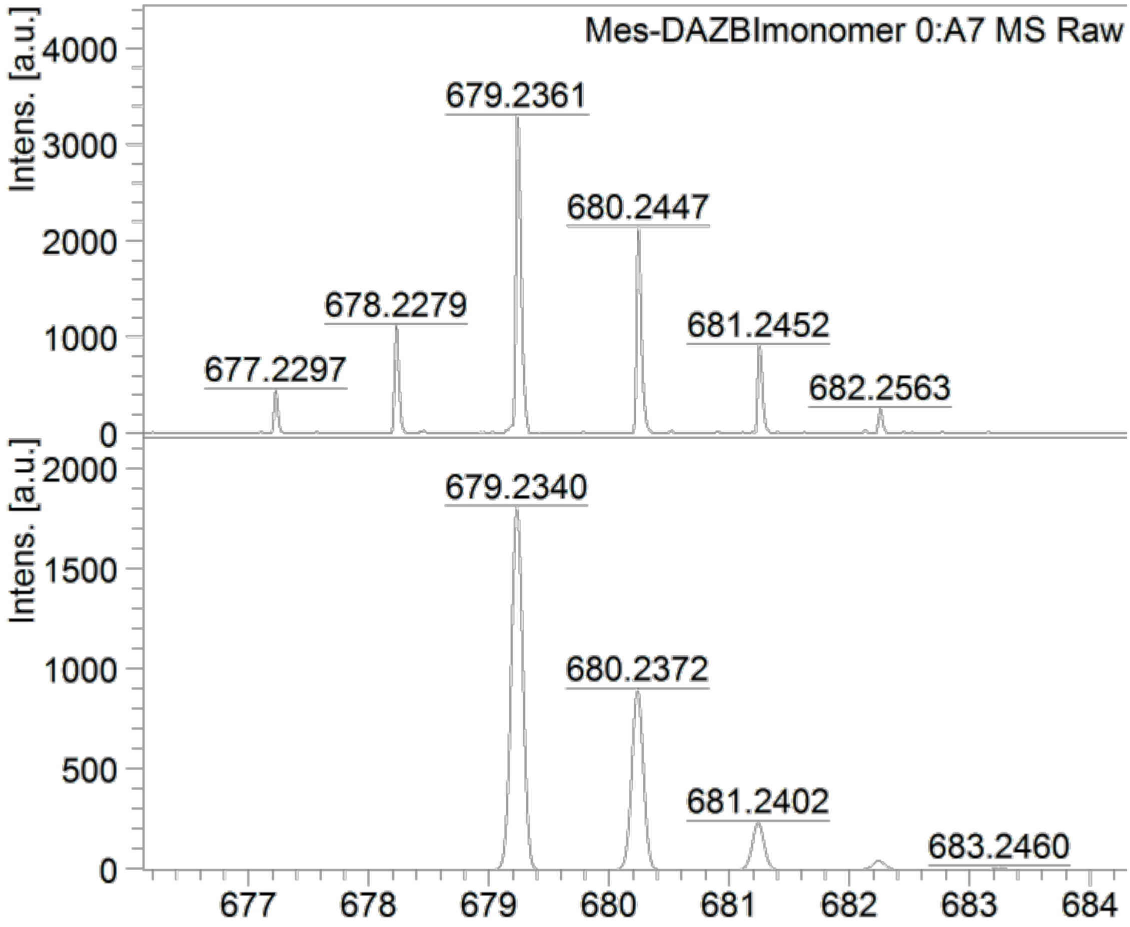

Acquisition Parameter

Date of acquisition
Acquisition method name

Aquisition operation mode
Voltage polarity
Number of shots
Name of spectrum used for calibration
Calibration reference list used

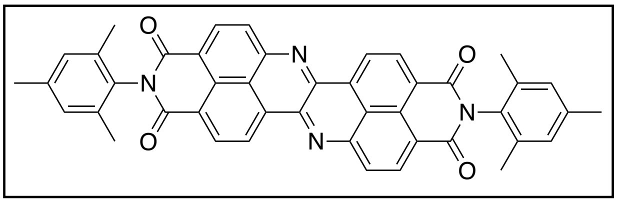

Instrument Info

User	tof-user
Instrument	FLEX-PC
Instrument type	

Bruker Daltonics flexAnalysis printed: 4/22/2022 3:31:59 PM

Figure S31. MALDI-TOF (positive mode) mass spectrum of **8a**.

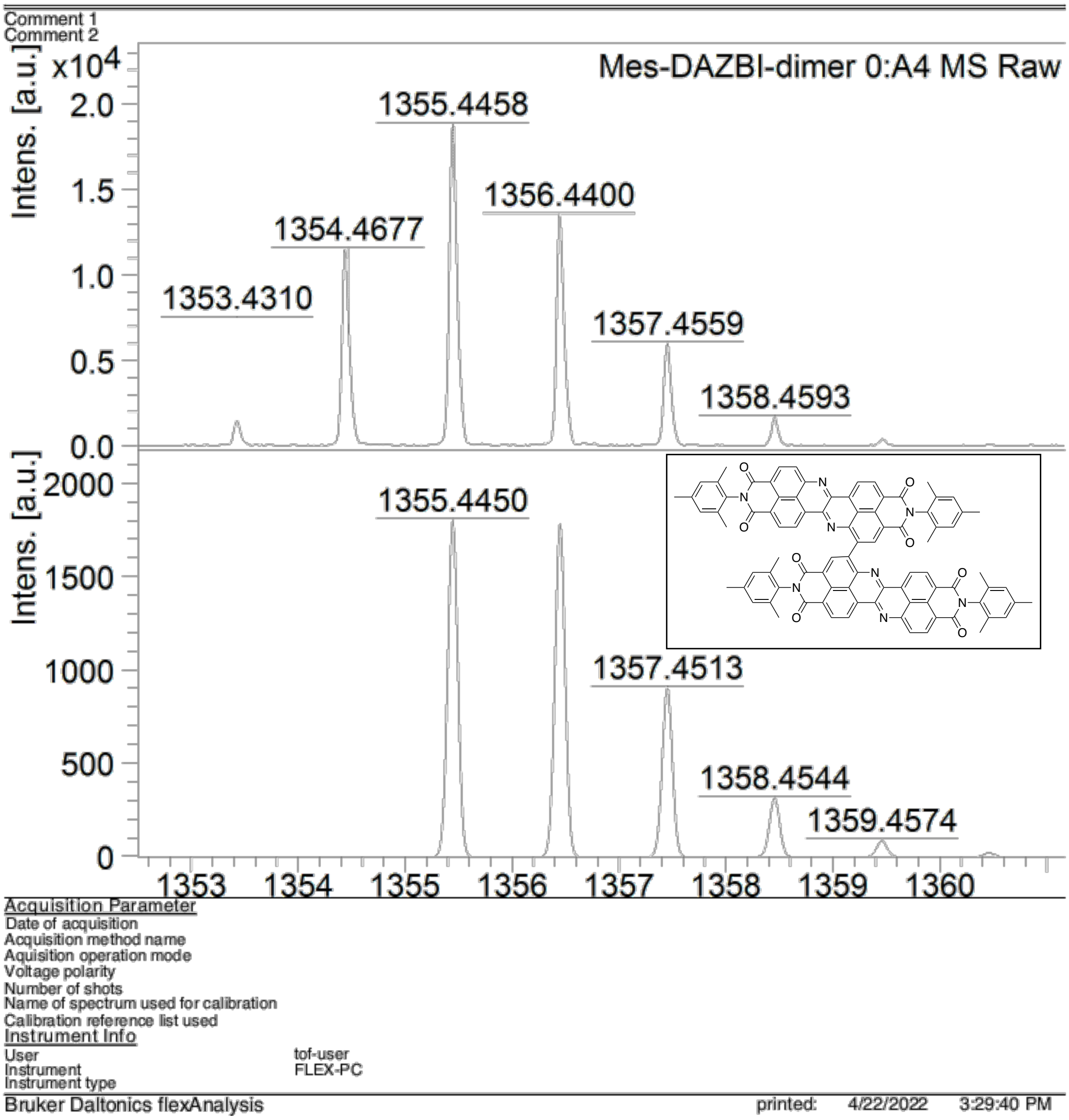

Figure S32. MALDI-TOF (positive mode) mass spectrum of **12a**.

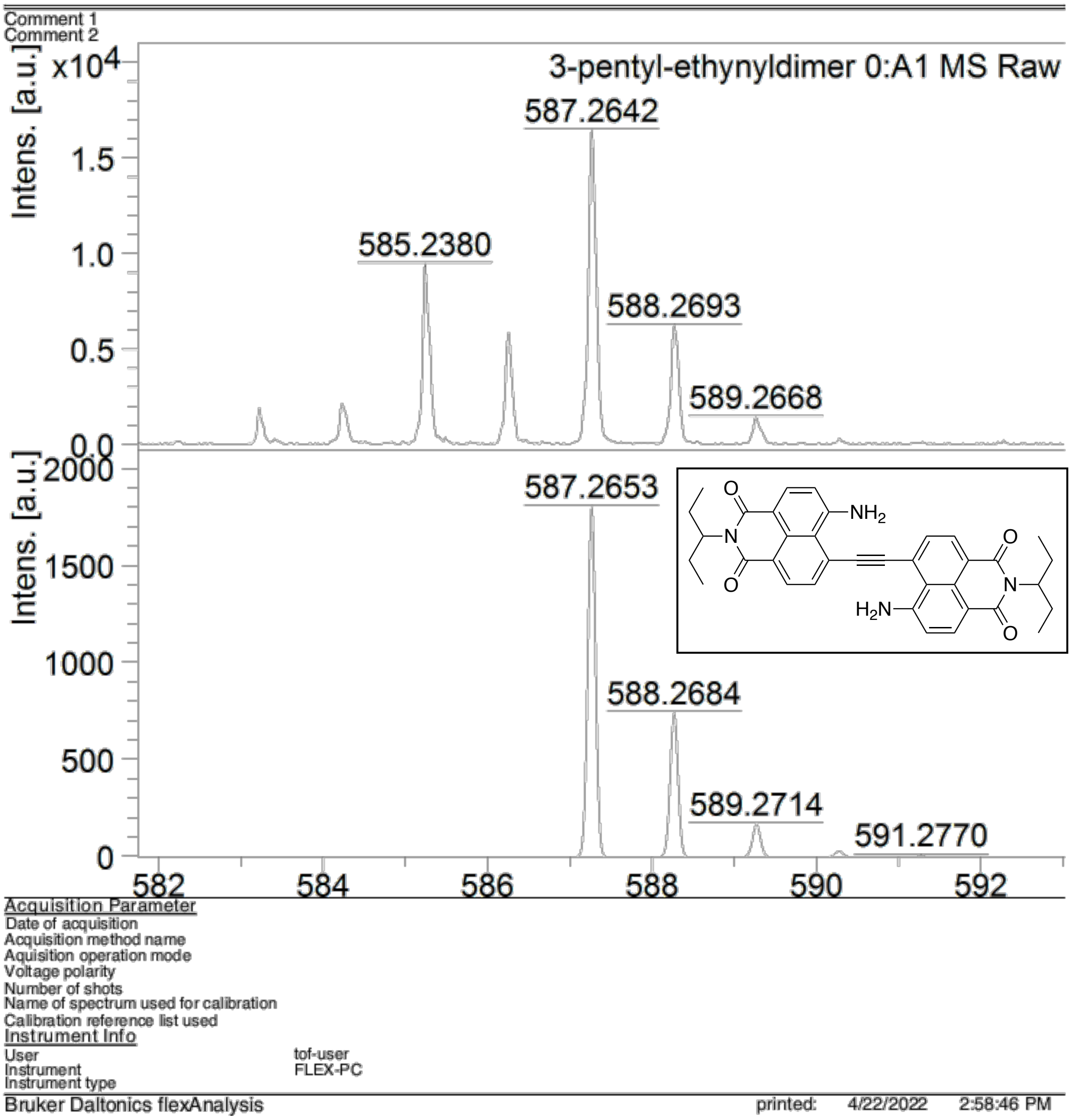

Figure S33. MALDI-TOF (positive mode) mass spectrum of **10b**.

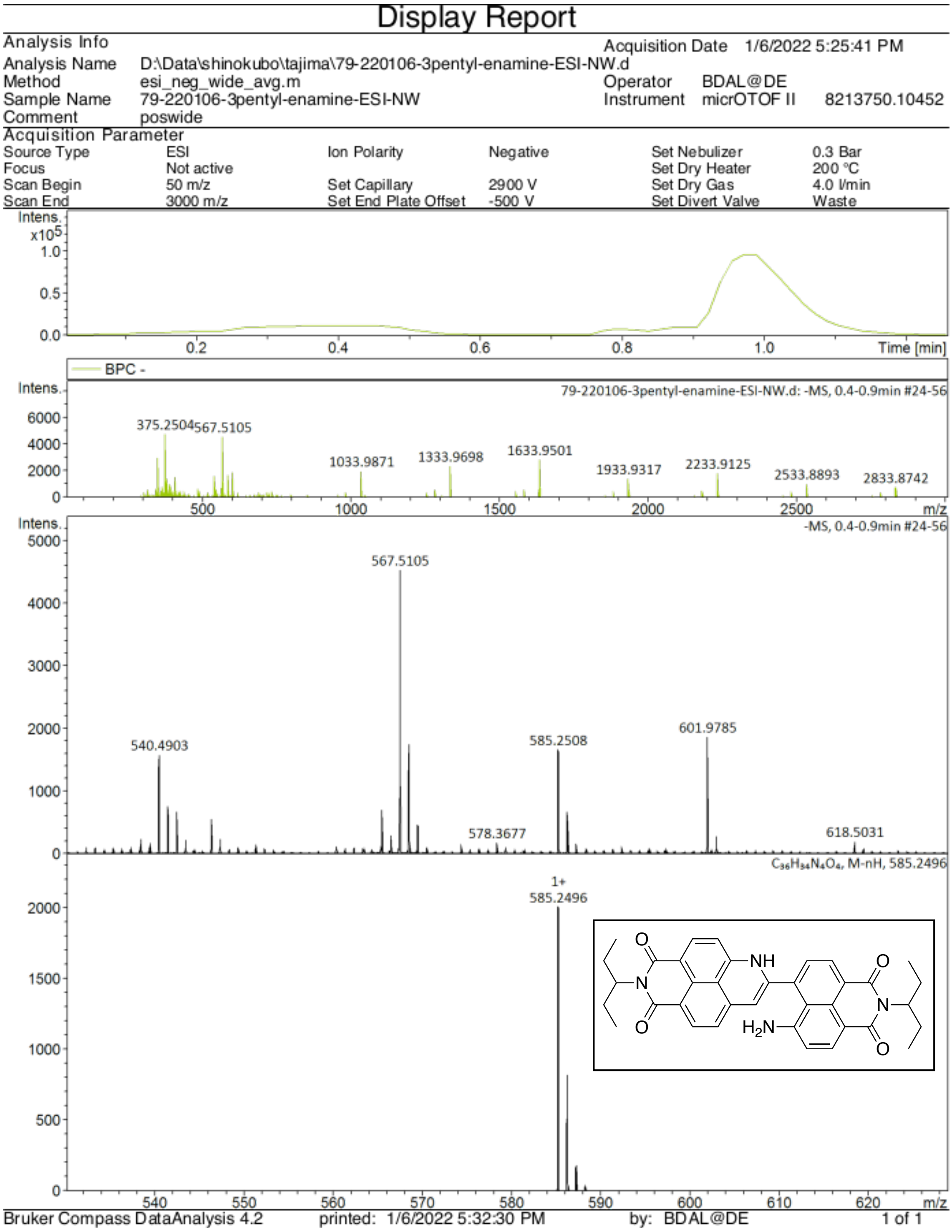

Figure S34. ESI-TOF (negative mode) mass spectrum of **11b**.

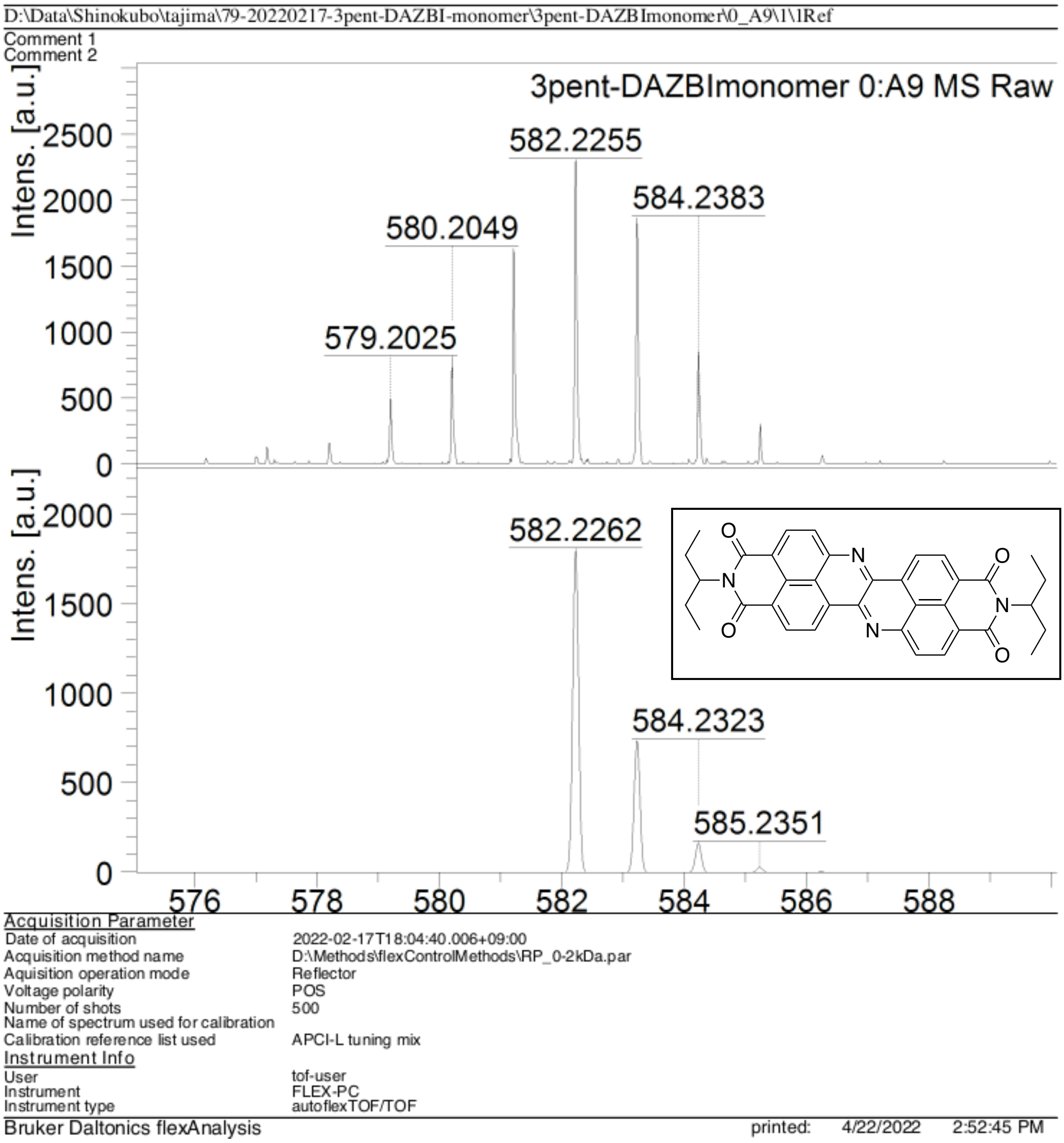

Figure S35. MALDI-TOF (positive mode) mass spectrum of **8b**.

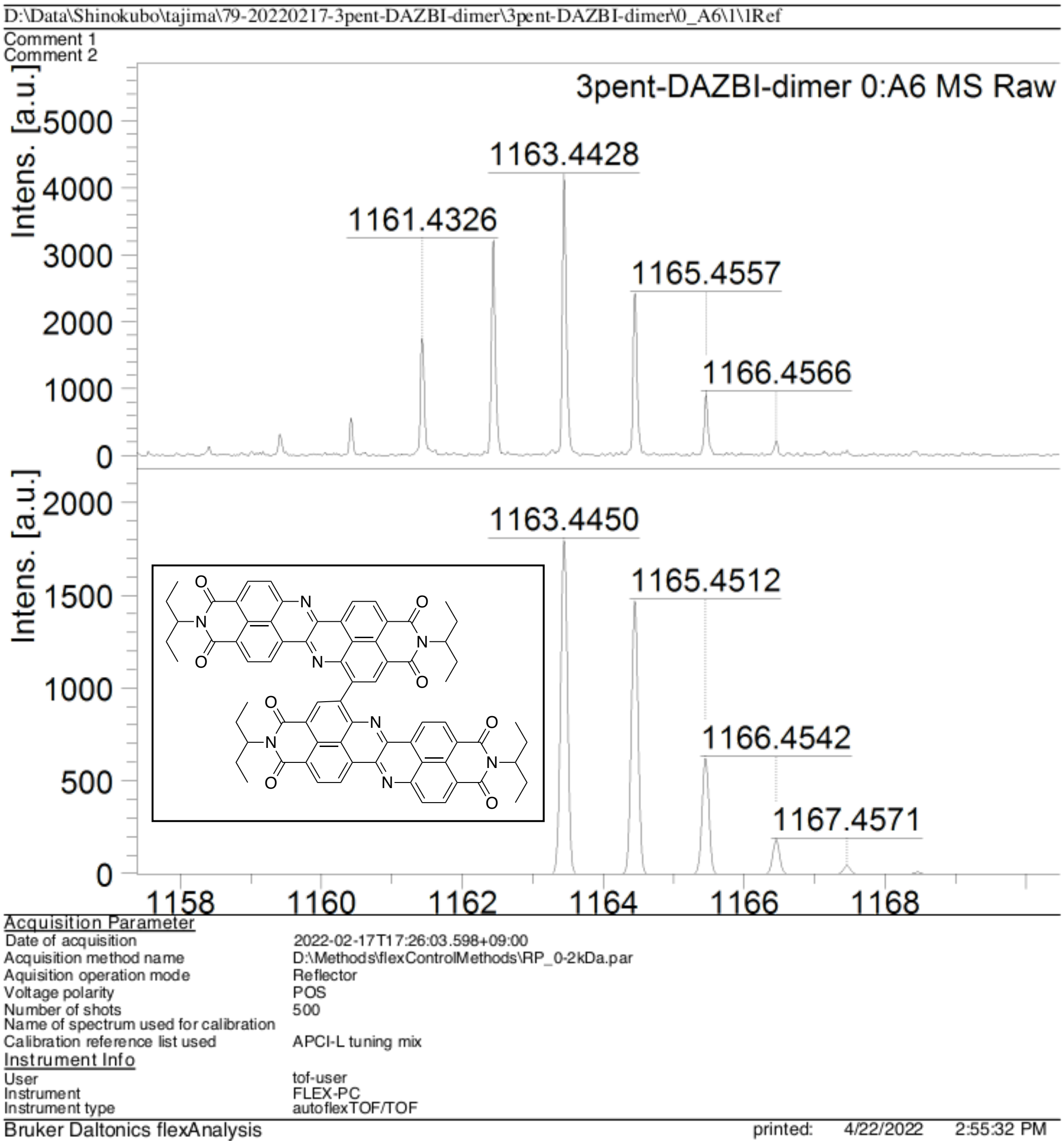

Figure S36. MALDI-TOF (positive mode) mass spectrum of **12b**.

Display Report

Analysis Info		Acquisition Date	2/16/2022 3:14:04 PM
Analysis Name	D:\Data\shinokubo\tajima\79-20220216-Mes-dnian-ESI-NL-calib.d		
Method	esi_neg_wide_avg.m	Operator	BDAL@DE
Sample Name	79-20220216-Mes-dnian-ESI-NL-calib	Instrument	micrOTOF II 8213750.10452
Comment	apci-neg-low		

Acquisition Parameter

Source Type	ESI	Ion Polarity	Negative	Set Nebulizer	0.3 Bar
Focus	Not active			Set Dry Heater	200 °C
Scan Begin	50 m/z	Set Capillary	2900 V	Set Dry Gas	4.0 l/min
Scan End	3000 m/z	Set End Plate Offset	-500 V	Set Divert Valve	Waste

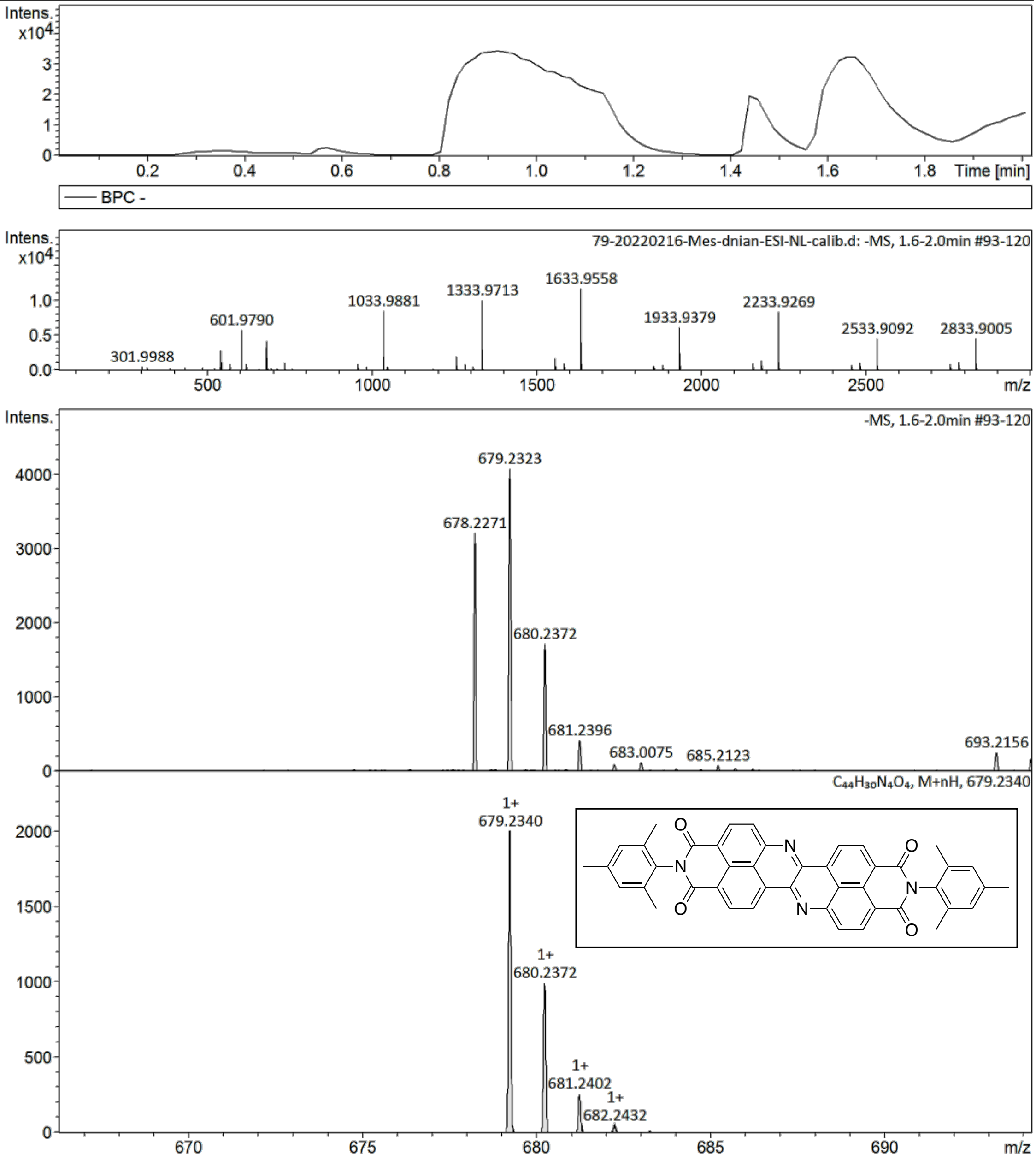

Bruker Compass DataAnalysis 4.2 printed: 2/16/2022 5:16:00 PM by: BDAL@DE 1 of 1

Figure S37. ESI-TOF (negative mode) mass spectrum of **13**.

Display Report

Analysis Info

		Acquisition Date	2/16/2022 3:30:20 PM
Analysis Name	D:\Data\shinokubo\tajima\79-20220216-Mes-dnian-ESI-PW-calib.d		
Method	esi_pos_wide_avg.m	Operator	BDAL@DE
Sample Name	79-20220216-Mes-dnian-ESI-PW-calib-2	Instrument	micrOTOF II 8213750.10452
Comment	apci-neg-low		

Acquisition Parameter

Source Type	ESI	Ion Polarity	Positive	Set Nebulizer	0.2 Bar
Focus	Not active			Set Dry Heater	200 °C
Scan Begin	55 m/z	Set Capillary	4500 V	Set Dry Gas	3.0 l/min
Scan End	3000 m/z	Set End Plate Offset	-500 V	Set Divert Valve	Waste

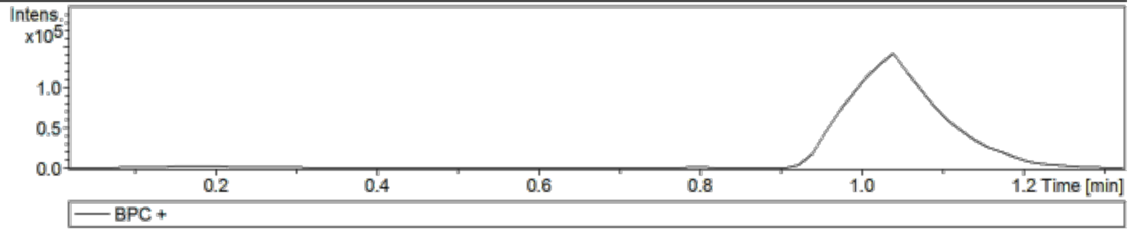

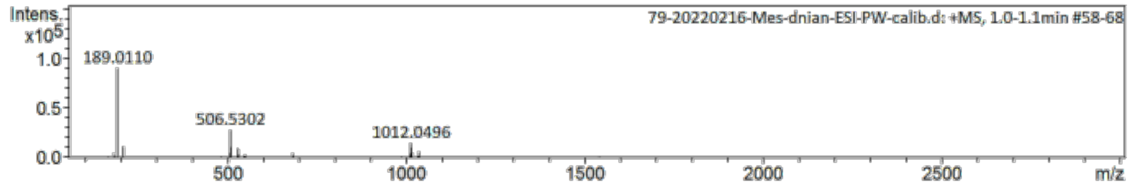

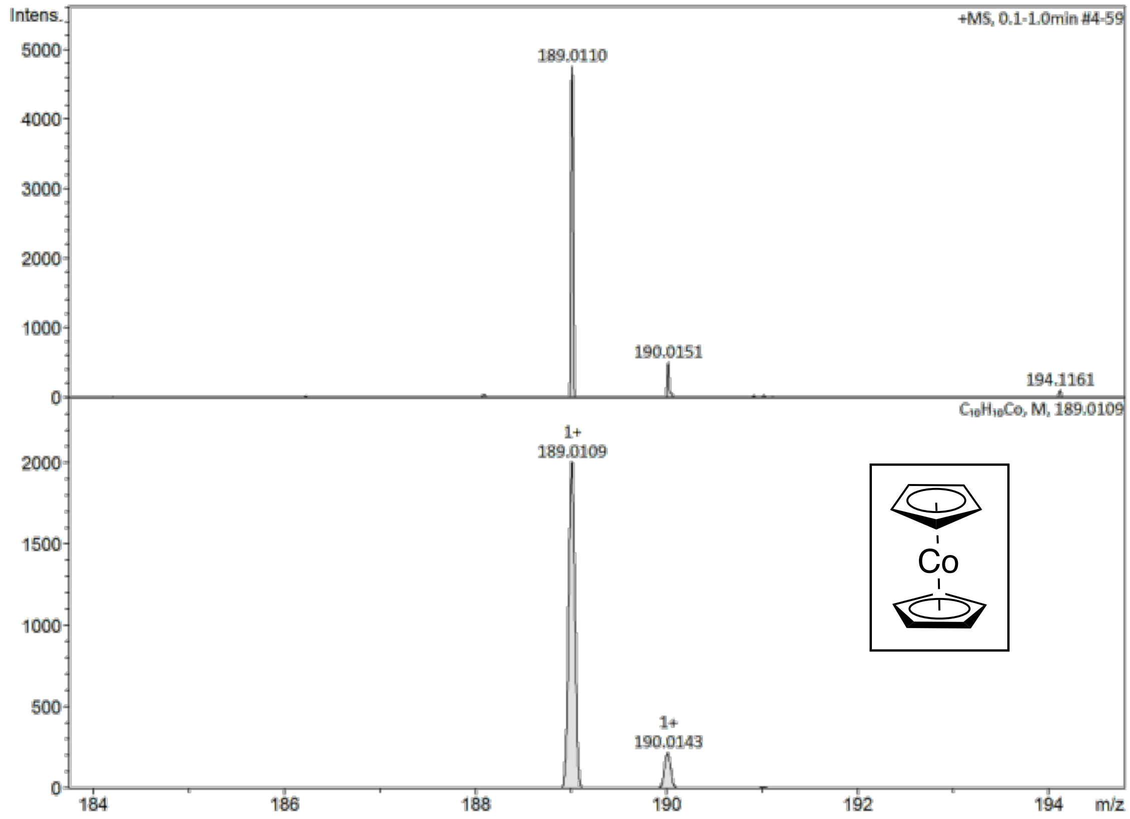

Figure S38. ESI-TOF (positive mode) mass spectrum of **13**.

D:\Data\Shinokubo\tajima\79-20220824-Mes-2H-DAZBI-2\0_A12\1\1Ref

Comment 1
Comment 2

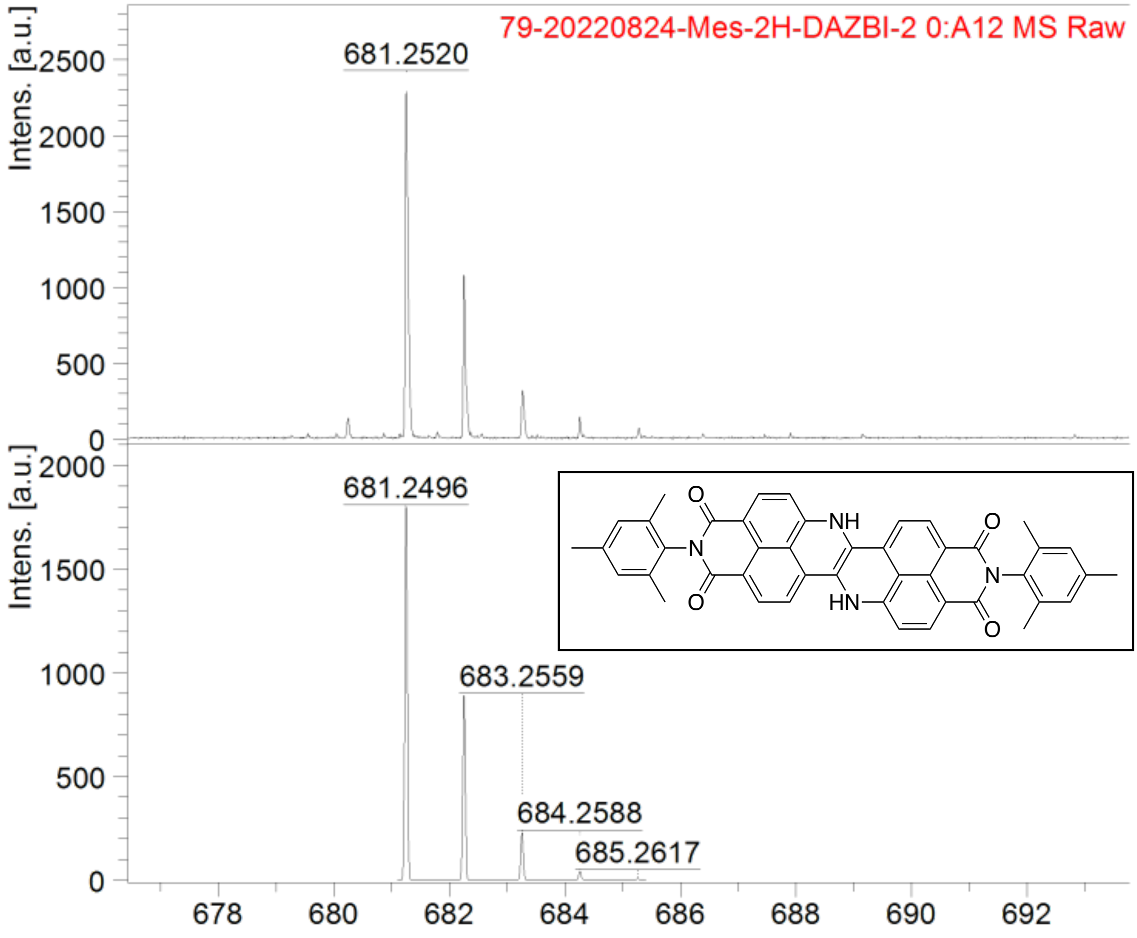

Acquisition Parameter

Date of acquisition	2022-08-24T20:28:20.004+09:00
Acquisition method name	D:\Methods\flexControlMethods\RP_0-2kDa.par
Aquisition operation mode	Reflector
Voltage polarity	POS
Number of shots	500
Name of spectrum used for calibration	
Calibration reference list used	APCI-L tuning mix

Instrument Info

User	tof-user
Instrument	FLEX-PC
Instrument type	autoflexTOF/TOF

Bruker Daltonics flexAnalysis printed: 8/24/2022 8:33:33 PM

Figure S39. MALDI-TOF (positive mode) mass spectrum of **14a**.

D:\Data\Shinokubo\tajima\79-20220905-3-pent-2H-DAZBI\0_A14\1\1Ref

Comment 1
Comment 2

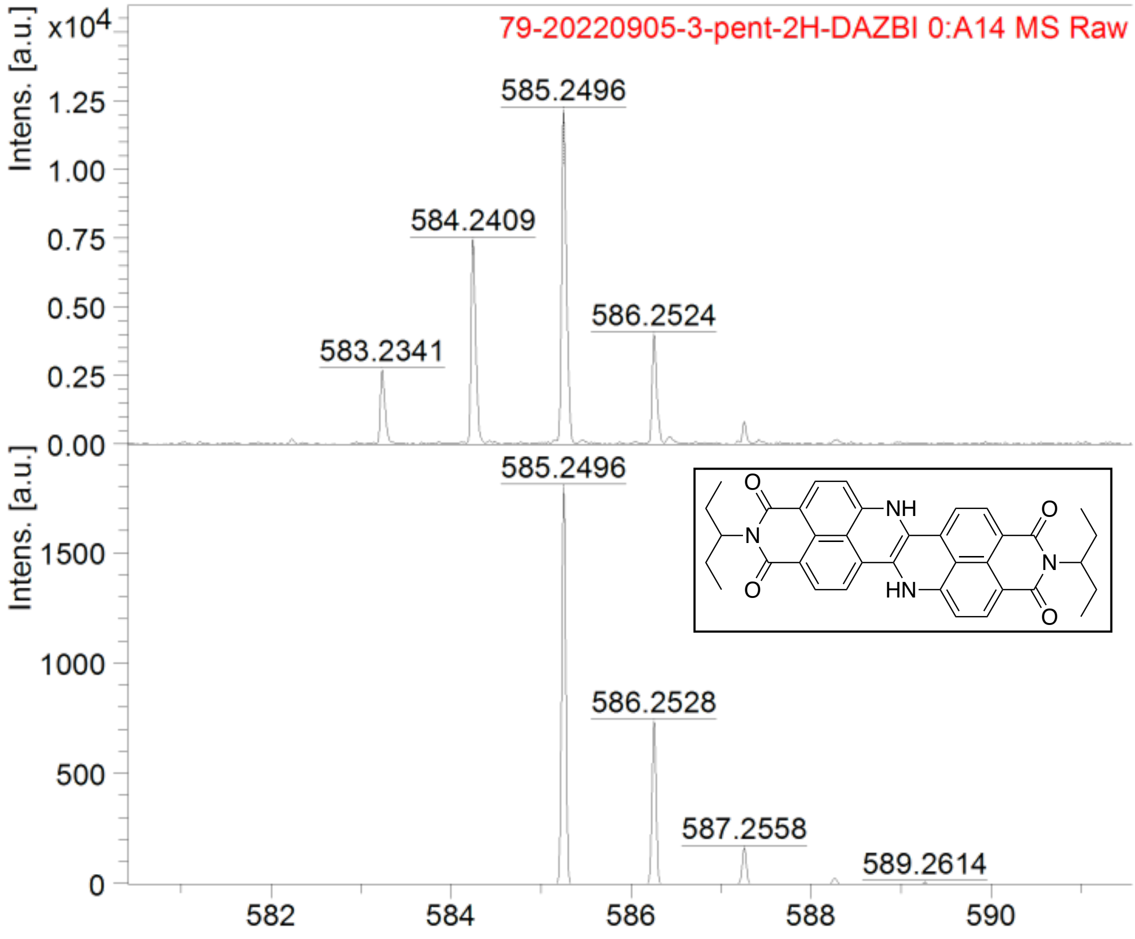

Acquisition Parameter

Date of acquisition	2022-09-05T13:24:30.677+09:00
Acquisition method name	D:\Methods\flexControlMethods\RP_0-2kDa.par
Aquisition operation mode	Reflector
Voltage polarity	POS
Number of shots	500
Name of spectrum used for calibration	
Calibration reference list used	APCI-L tuning mix

Instrument Info

User	tof-user
Instrument	FLEX-PC
Instrument type	autoflexTOF/TOF

Bruker Daltonics flexAnalysis printed: 9/5/2022 1:28:06 PM

Figure S40. MALDI-TOF (positive mode) mass spectrum of **14b**.

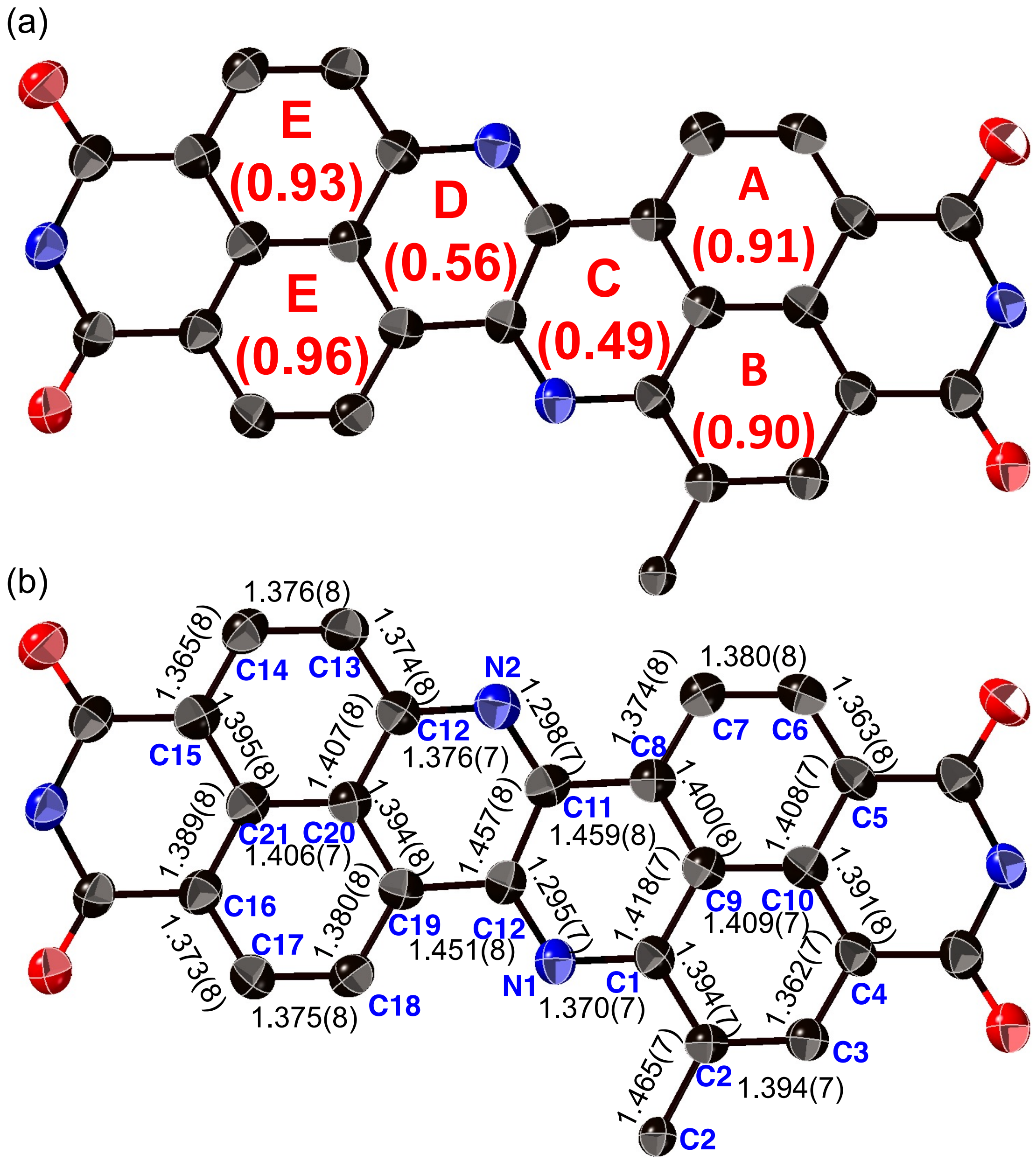

Figure S41. (a) HOMA values and (b) bond lengths of **12b** obtained by X-ray diffraction analysis. Thermal ellipsoids are shown at the 50% probability level. 3-Pentyl groups are omitted.

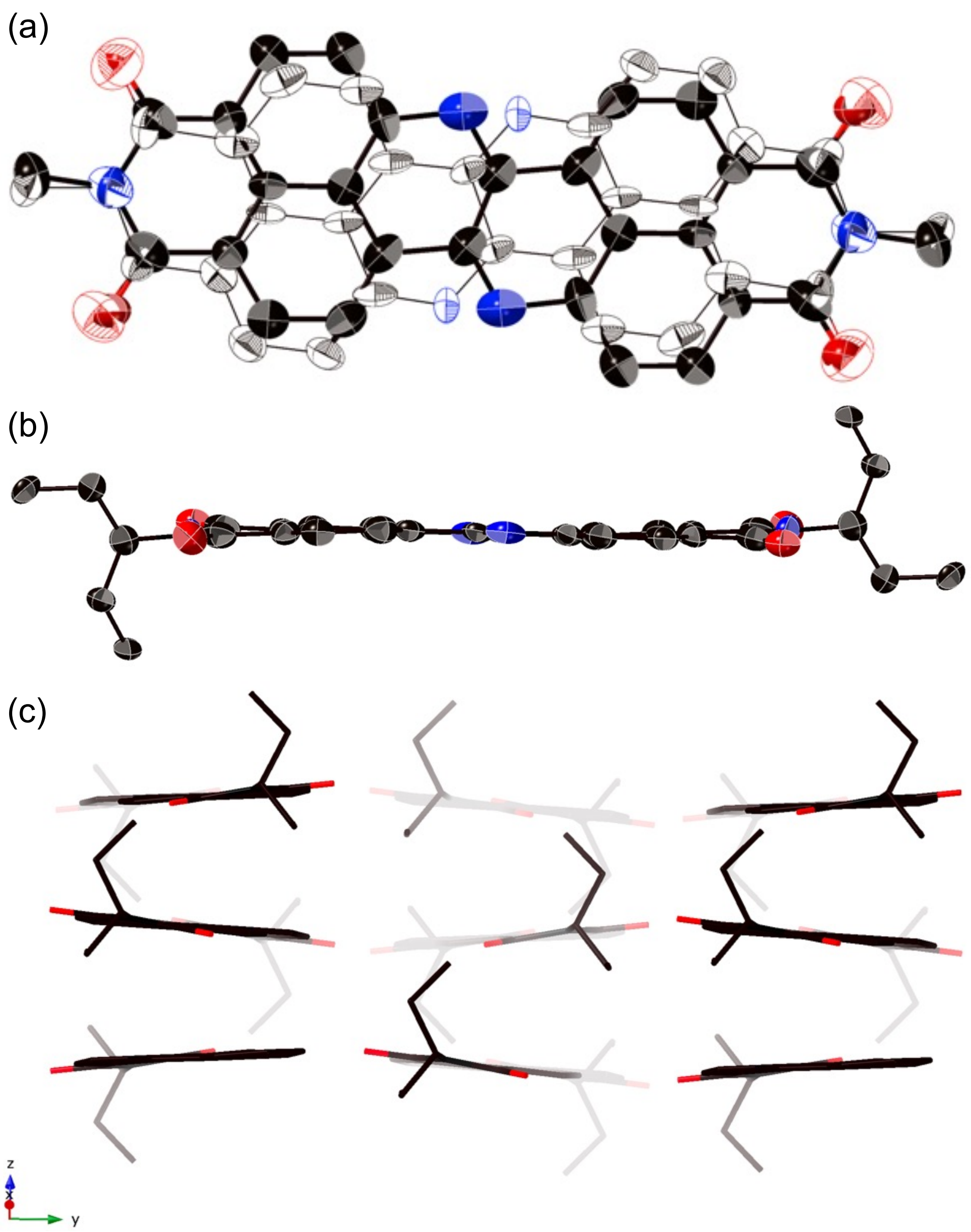

Figure S42. X-ray crystal structure of **8b**. (a) Top view, (b) side view, and (c) packing structure. Thermal ellipsoids are shown at the 50% probability level. Hollow and solid ellipsoids show the disorder over two positions (occupancy: solid spheres/hollow spheres = 0.58/0.42). All hydrogen atoms are omitted for clarity.

Table S1. Crystallographic data of **8a**, **8b**, and **12b**.

compound	**8a**	**8b**	**12b**
Formula	$C_{44}H_{30}N_4O_4$	$C_{36}H_{30}N_4O_4$	$C_{32}H_{26}N_2O_4$
Formula weight	678.75	582.64	15926.7(12)
Crystal system	triclinic	monoclinic	orthorhombic
Space group	*P*-1 (No. 2)	*C*2/*c* (No. 15)	*Fdd*2 (No. 43)
Crystal color	blue	blue	black
Crystal description	prism	prism	prism
a [Å]	8.0994(3)	20.1187(9)	34.9018(13)
b [Å]	11.7159(8)	16.7884(9)	59.974(3)
c [Å]	21.0793(12)	8.3834(3)	7.6088(3)
α [°]	102.210(5)	90	90
β [°]	97.445(4)	102.468(4)	90
γ [°]	96.128(4)	90	90
V [Å^3]	1919.86(19)	2764.8(2)	15926.7(12)
Z	2	4	2
d_{calcd} [g cm^{-3}]	1.428	1.400	1.324
R_1 ($I > 2\sigma(I)$)	0.0840	0.0541	0.0706
wR_2 (all data)	0.2513	0.1605	0.1863
Goodness-of-fit	1.035	1.052	1.015
Temperature [K]	93(2)	93(2)	93(2)
Solvent	*o*-dichlorobenzene /cyclohexane	toluene/MeOH	*o*-xylene/octane
CCDC No.	2211115	2211112	2211113

Table S2. Crystallographic data of **13**.

compound	**13**
Formula	$C_{64}H_{50}N_4O_4Co_2$
Formula weight	1056.99
Crystal system	monoclinic
Space group	$P2_1/c$ (No. 14)
Crystal color	green
Crystal description	prism
a [Å]	9.5098(2)
b [Å]	20.5556(3)
c [Å]	15.4387(2)
α [°]	90
β [°]	95.024(2)
γ [°]	90
V [Å^3]	3006.36(9)
Z	2
d_{calcd} [g cm^{-3}]	1.371
R_1 ($I > 2\sigma(I)$)	0.0685
wR_2 (all data)	0.2028
Goodness-of-fit	1.033
Temperature [K]	93(2)
Solvent	acetonitrile/toluene
CCDC No.	2211114

6. Electrochemistry

Cyclic and differential pulse voltammograms were obtained under the following conditions; solvent: CH_2Cl_2 or THF. electrolyte: 0.1 M Bu_4NPF_6, working electrode: glassy carbon, counter electrode: Pt, reference electrode: $Ag/AgNO_3$, scan rate: 0.1 V/s.

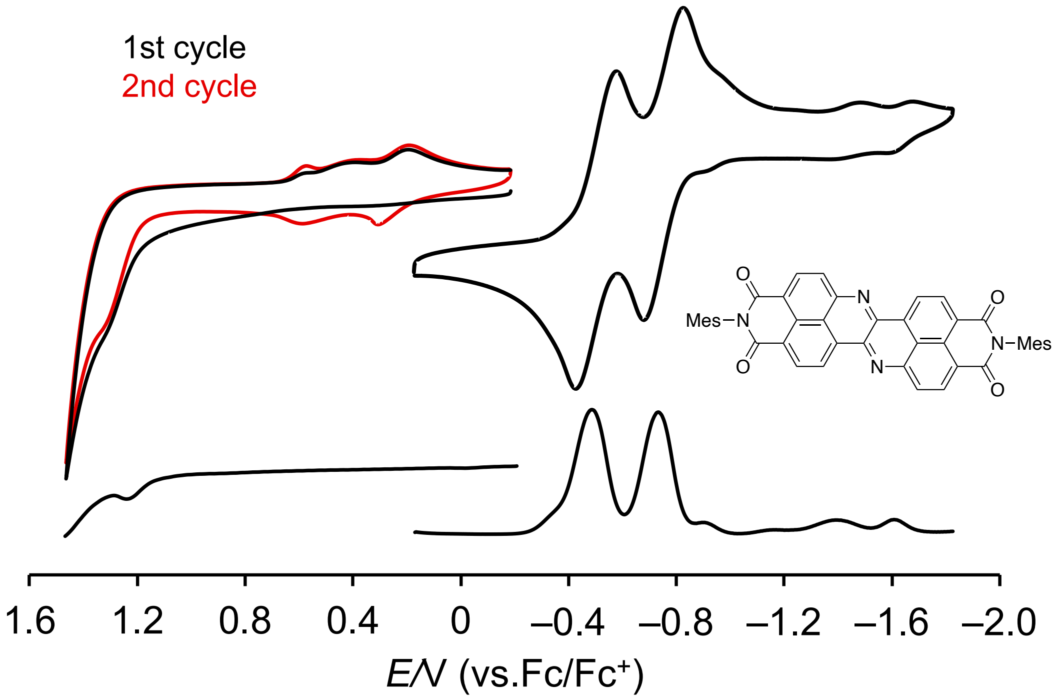

Figure S43. Cyclic and differential pulse voltammograms of **8a** in CH_2Cl_2.

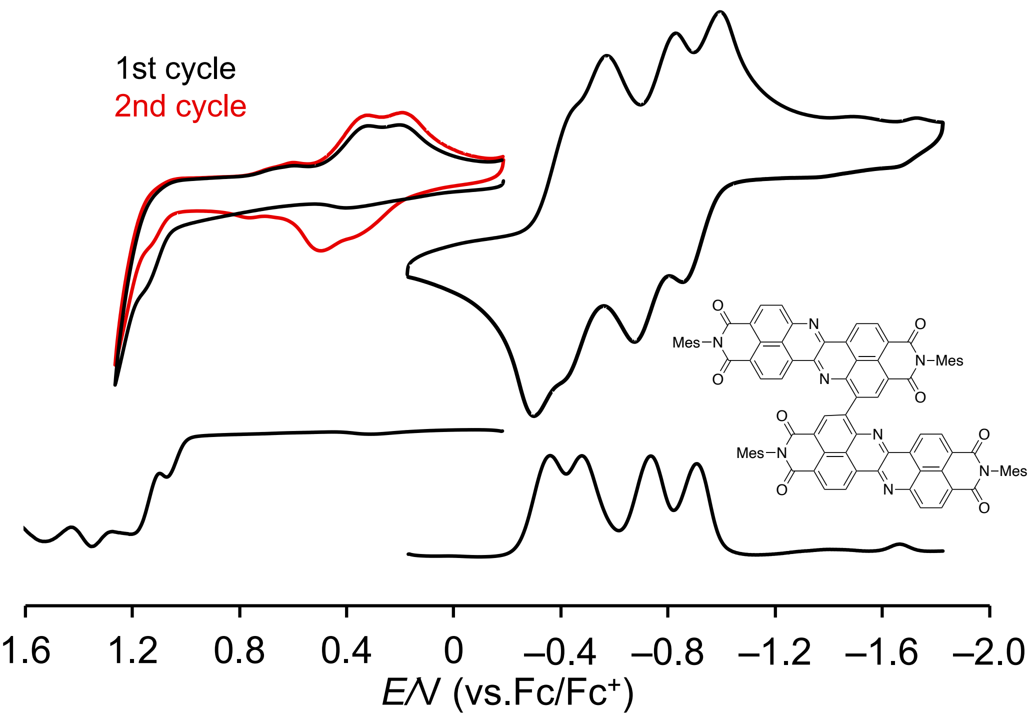

Figure S44. Cyclic and differential pulse voltammograms of **12a** in CH_2Cl_2.

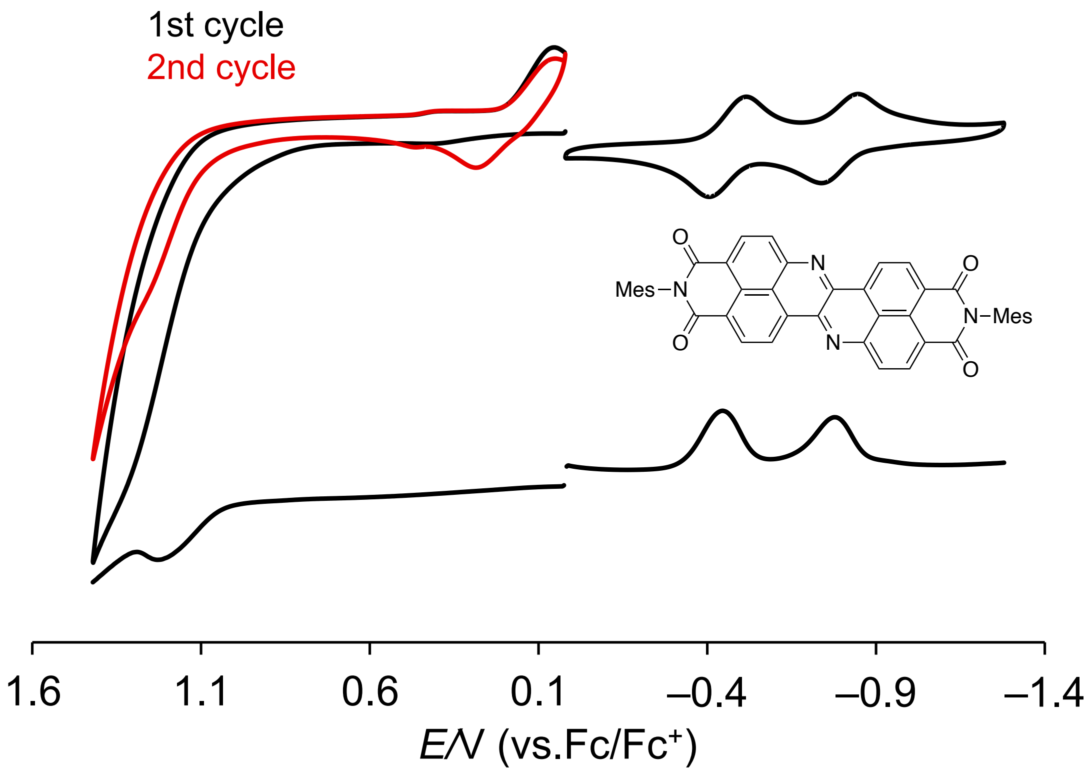

Figure S45. Cyclic and differential pulse voltammograms of **8a** in THF.

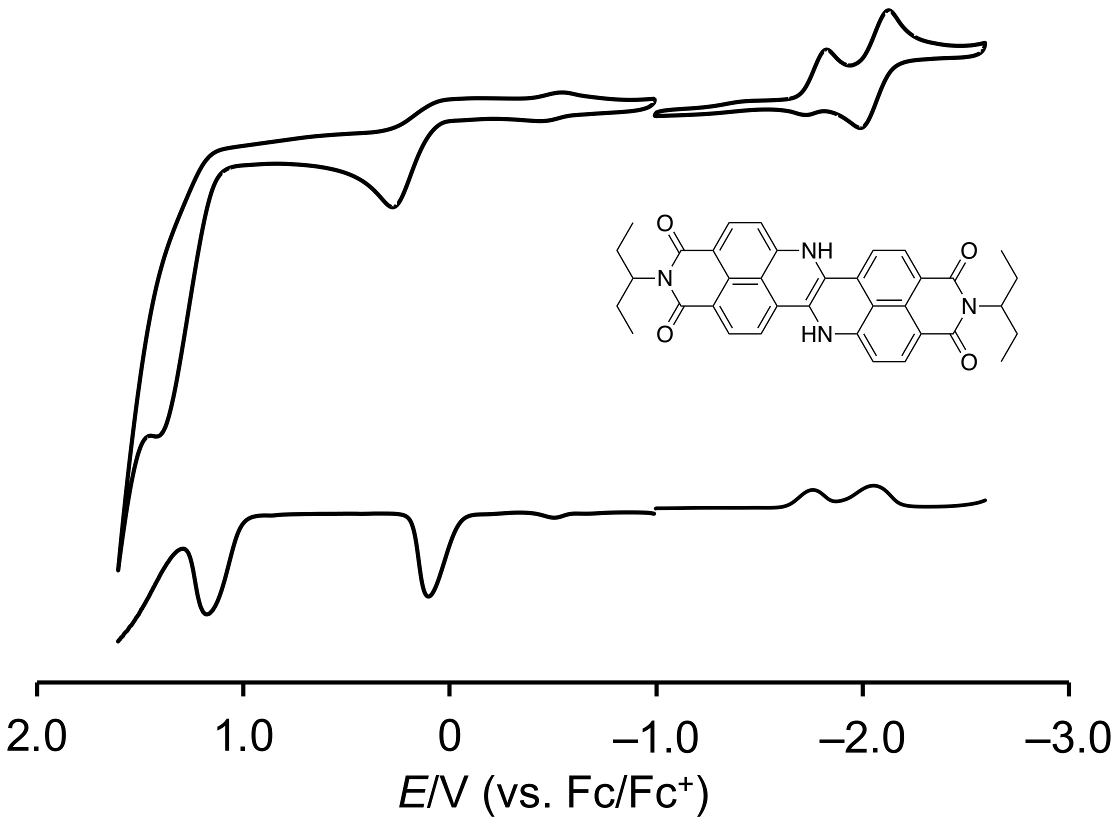

Figure S46. Cyclic and differential pulse voltammograms of **14b** in THF.

7. DFT calculations

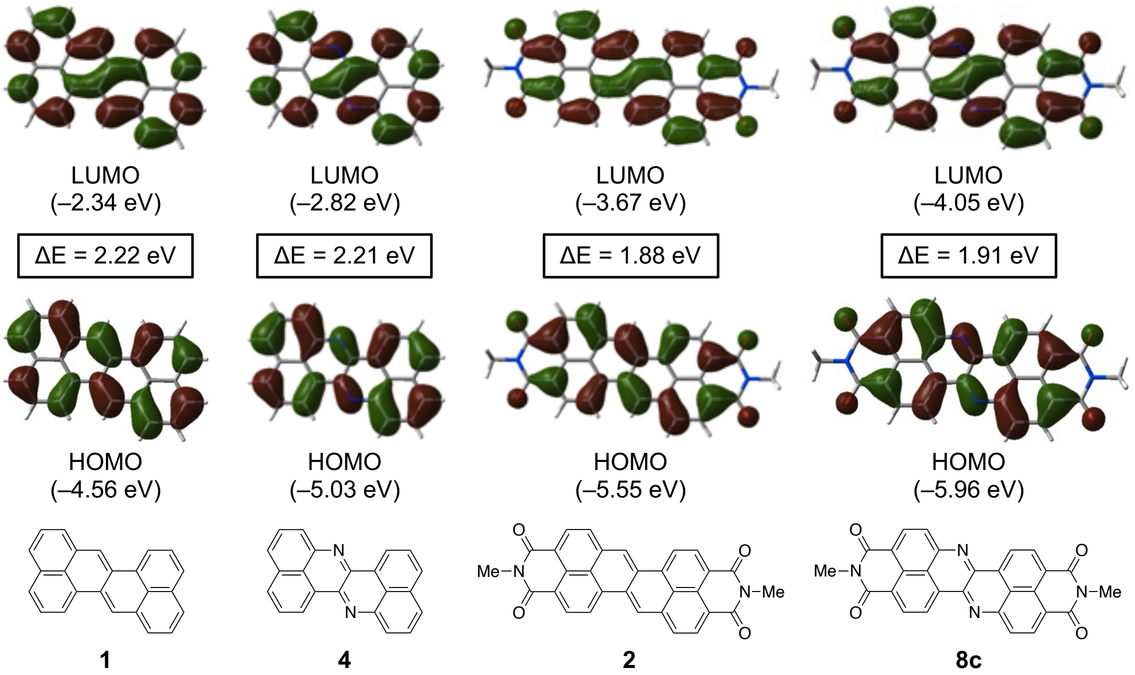

Figure S47. Calculated molecular orbitals of **1**, **4**, **2**, and **8c** (isovalue = 0.02). Calculation levels: B3LYP/6-31G(d).

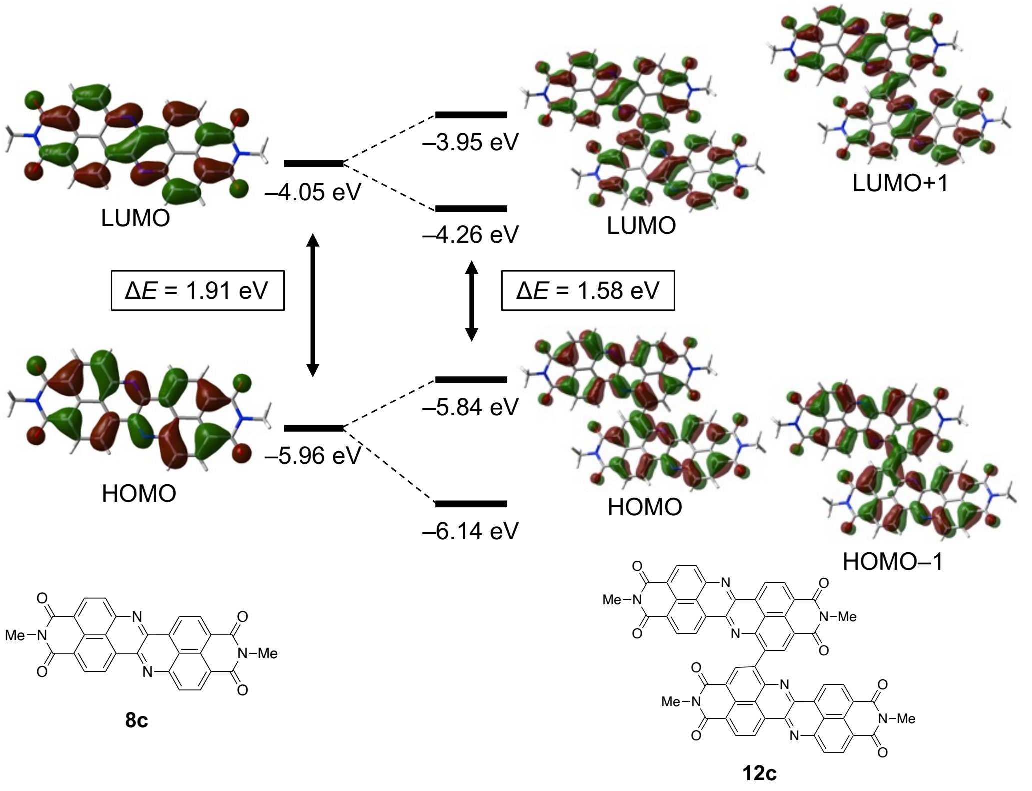

Figure S48. Calculated molecular orbitals of **8c** and **12c** (isovalue = 0.02). Calculation levels:

B3LYP/6-31G(d).

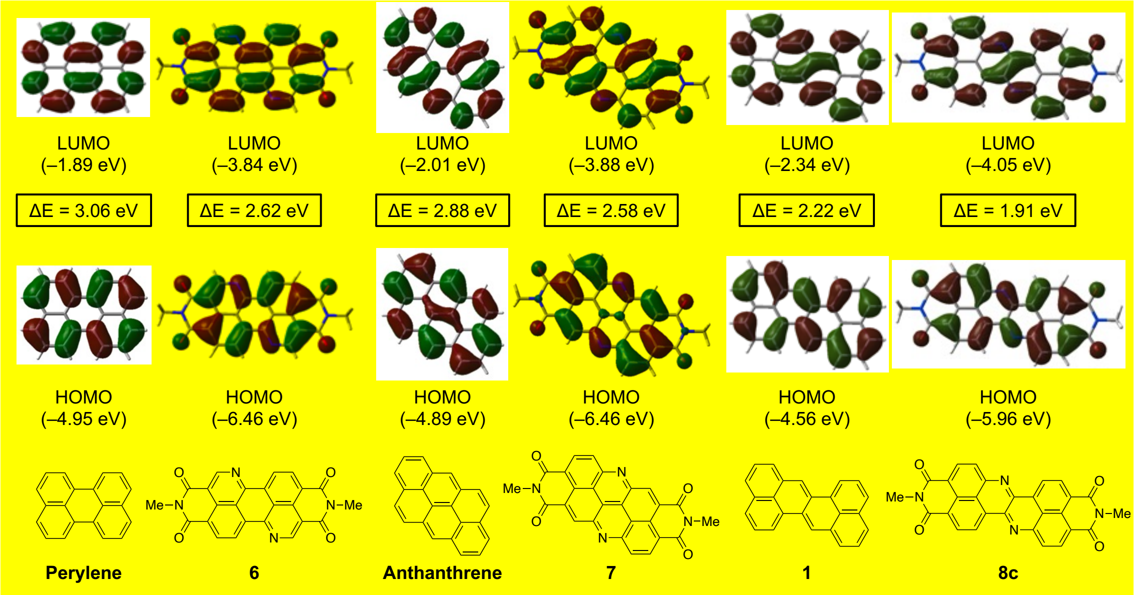

Figure S49. Calculated molecular orbitals of perylene, **6**, anthanthrene, **7**, **1**, and **8c** (isovalue = 0.02). Calculation levels: B3LYP/6-31G(d).

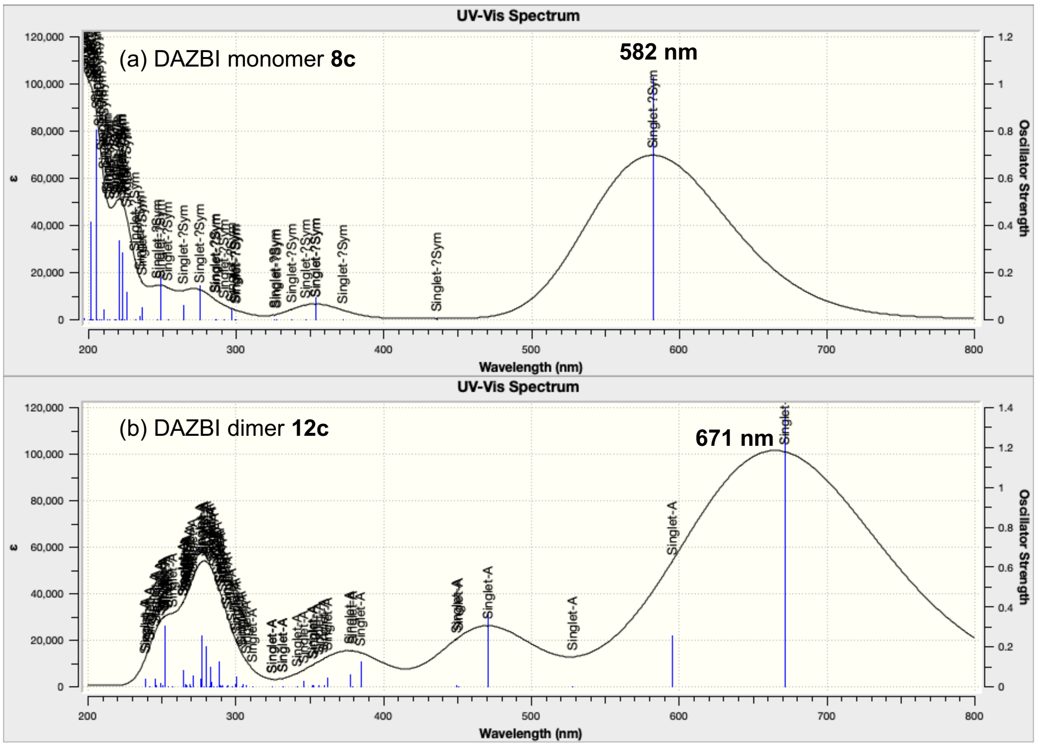

Figure S50. Simulated absorption spectra of (a) **8c** and (b) **12c**. Calculation levels: TD-cam-B3LYP/6-31+G(d,p).

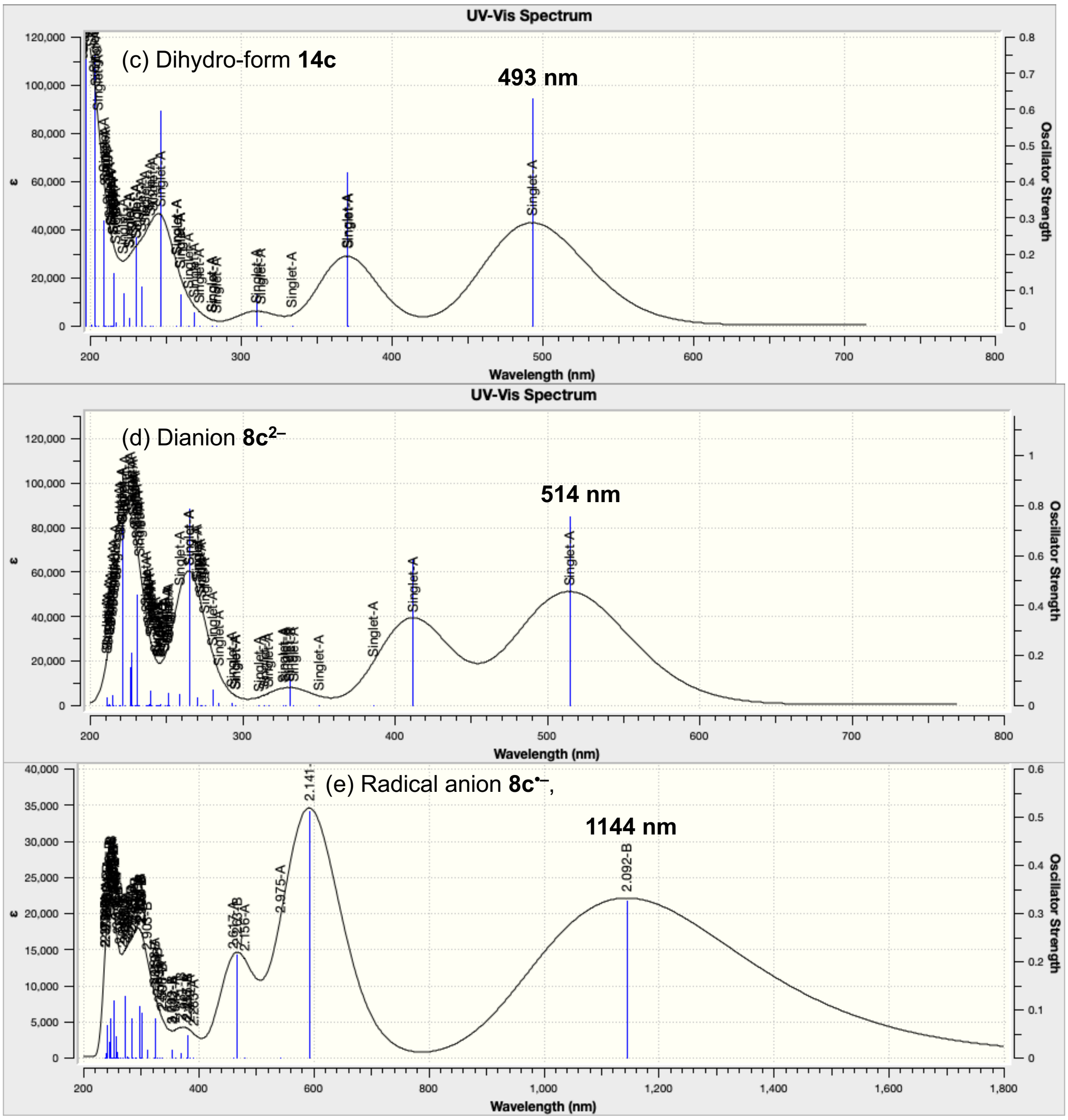

Figure S51. Simulated absorption spectra of (a) **8c** and (b) **12c**. Calculation levels: TD-cam-B3LYP/6-31+G(d,p) for **14c** and **8c**$^{2-}$ and TD-ucam-B3LYP/6-31+G(d,p) for **8c**$^{\bullet-}$.

8. IR spectra

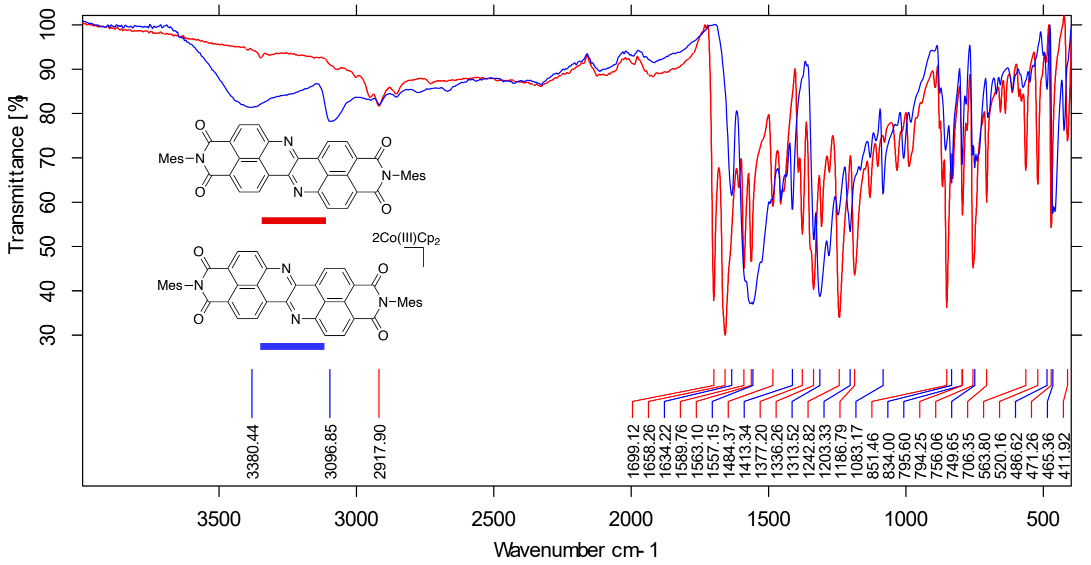

Figure S52. FT-IR spectra of **8a** (red line) and **13** (blue line).

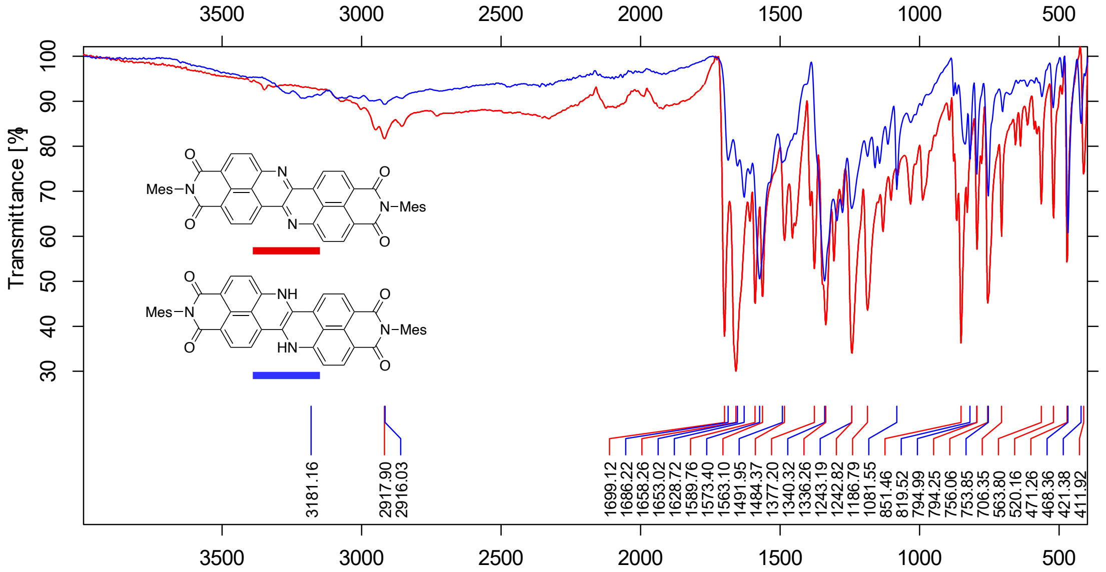

Figure S53. FT-IR spectra of **8a** (red line) and **14a** (blue line).

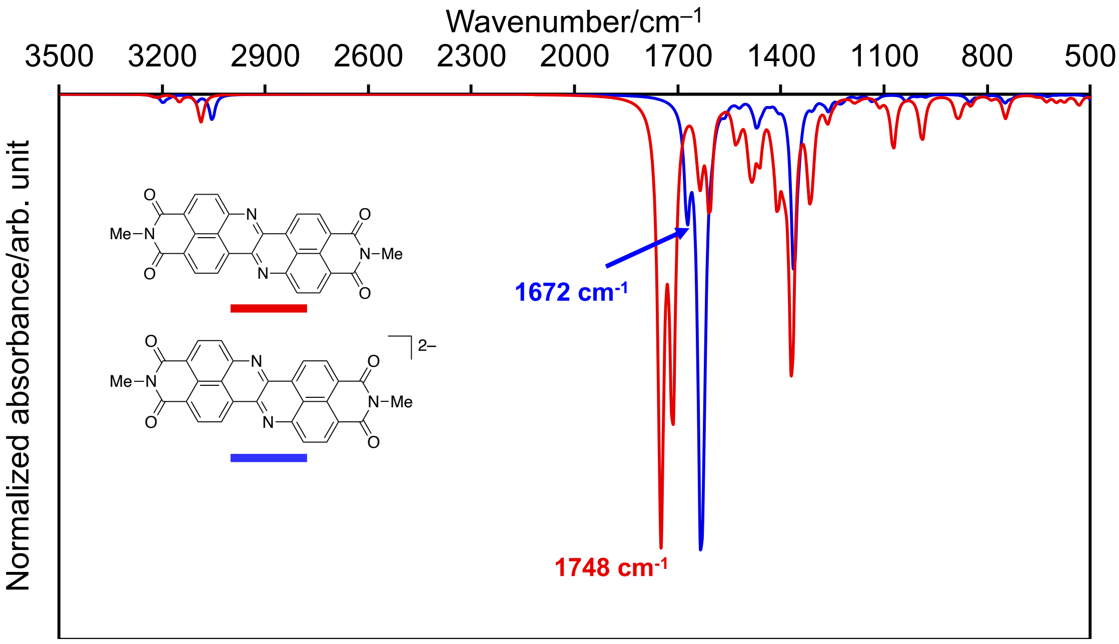

Figure S54. Simulated IR spectra of **8c** (red line) and its dianion (blue line). Calculation levels: B3LYP/6-31+G(d)//B3LYP/6-31+G(d). Simulated spectra are output with 10 cm1 of half-width at half height.

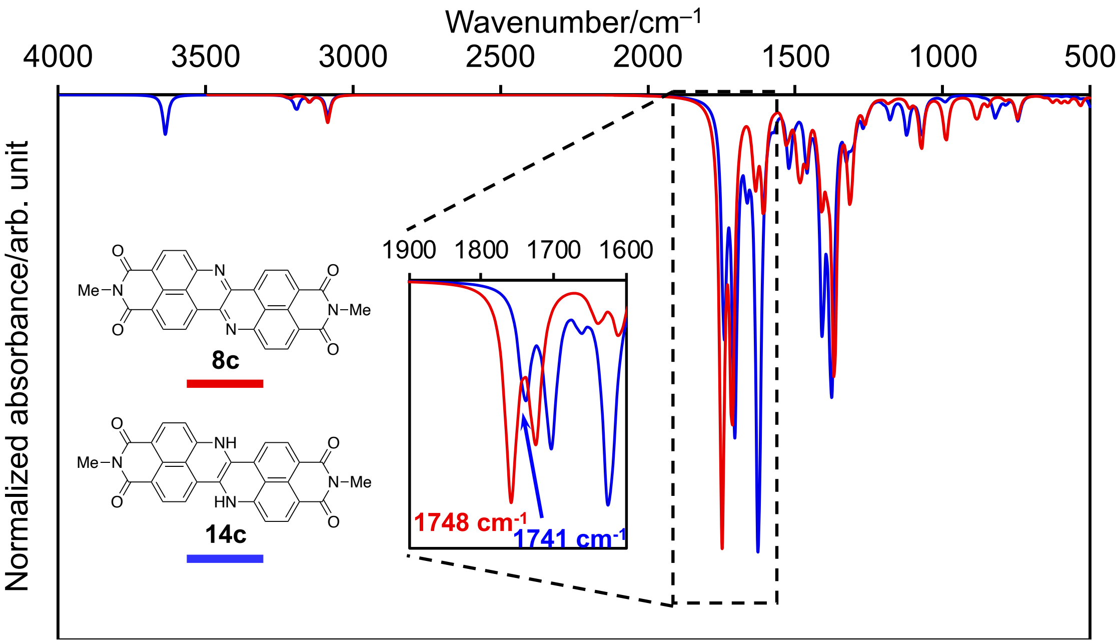

Figure S55. Simulated IR spectra of **8c** (red line) and **14c** (blue line). Calculation levels: B3LYP/6-31+G(d)//B3LYP/6-31+G(d). Simulated spectra are output with 10 cm^{-1} of half-width at half height.

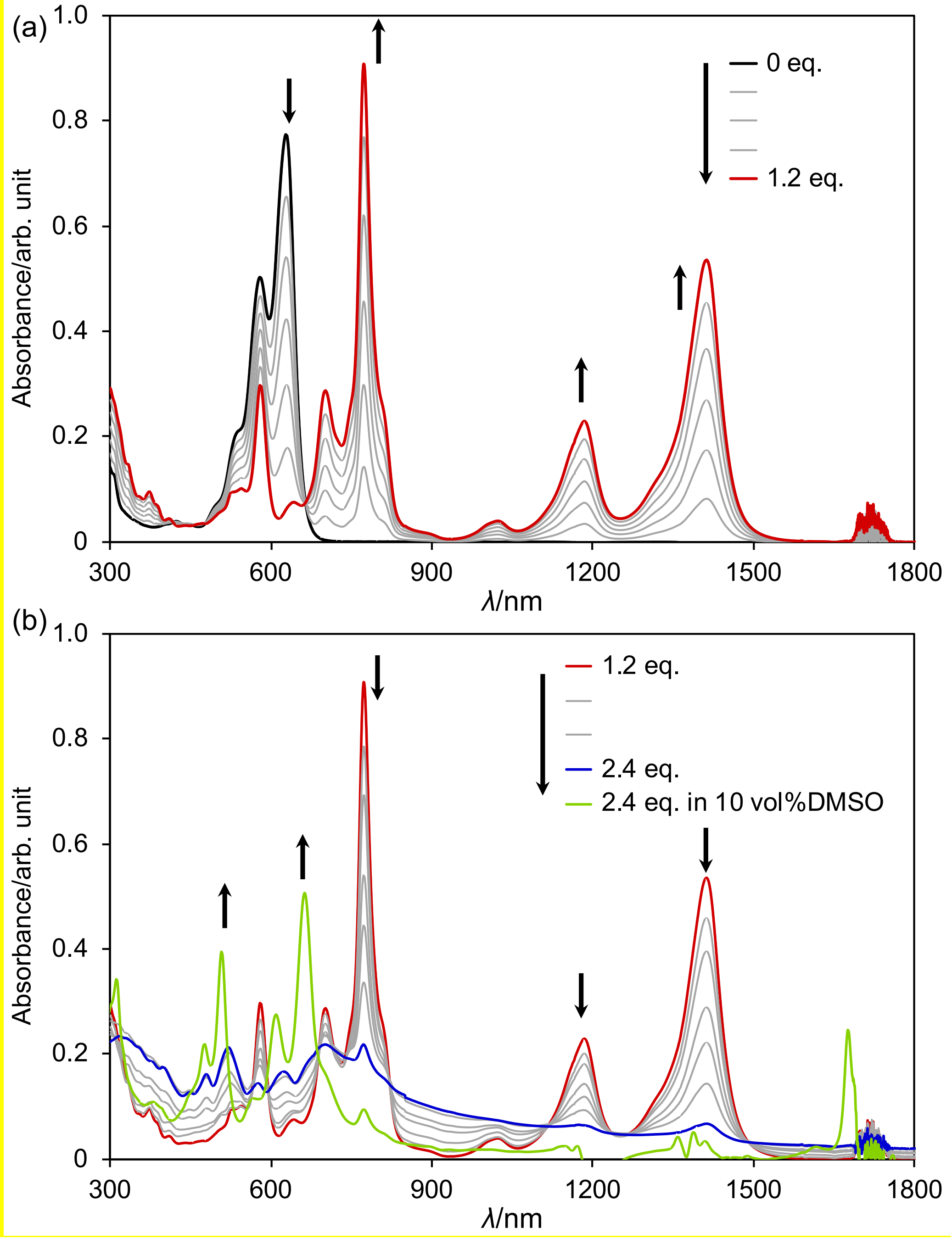

Figure S56. Change in the absorption spectrum of **8a** in THF (9.9×10^{-6} M) upon addition of cobaltocene. (a) from 0 eq. to 1.2 eq. and (b) from 1.2 eq. to 2.4 eq. The spectrum after the addition of 2.4 eq. of cobaltocene (blue line) is broad due to the precipitation of dianion **13**. Dianion **13** dissolved by the following addition of 10 vol% DMSO (green line); *λ*: wavelength.

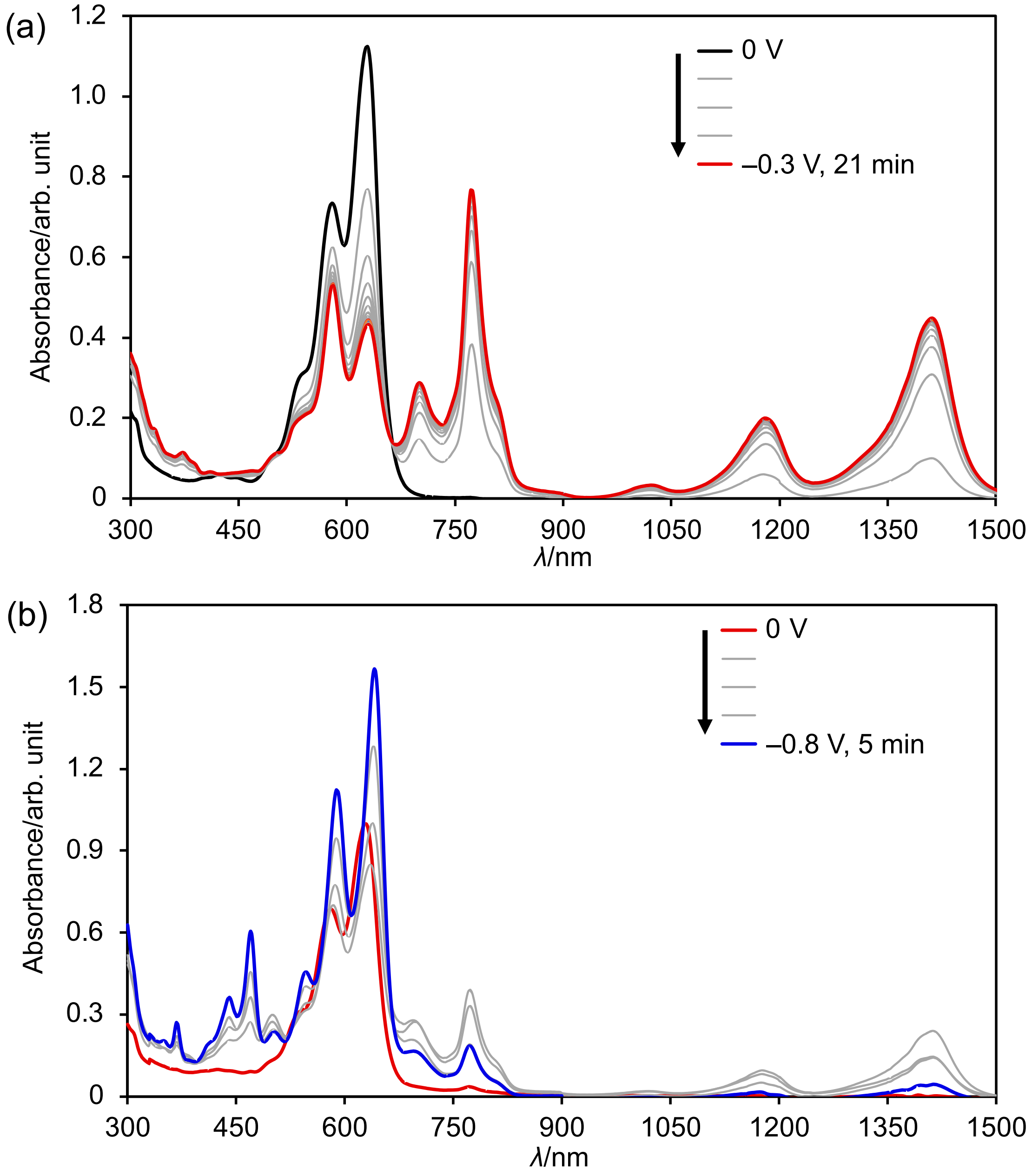

Figure S57. Absorption spectra of **8a** upon application of a voltage in THF in the presence of *n*Bu_4NPF_6 as electrolyte (a) from 0 V to –0.3 V and (b) from 0 V to –0.8 V (vs. Ag/Ag^+).

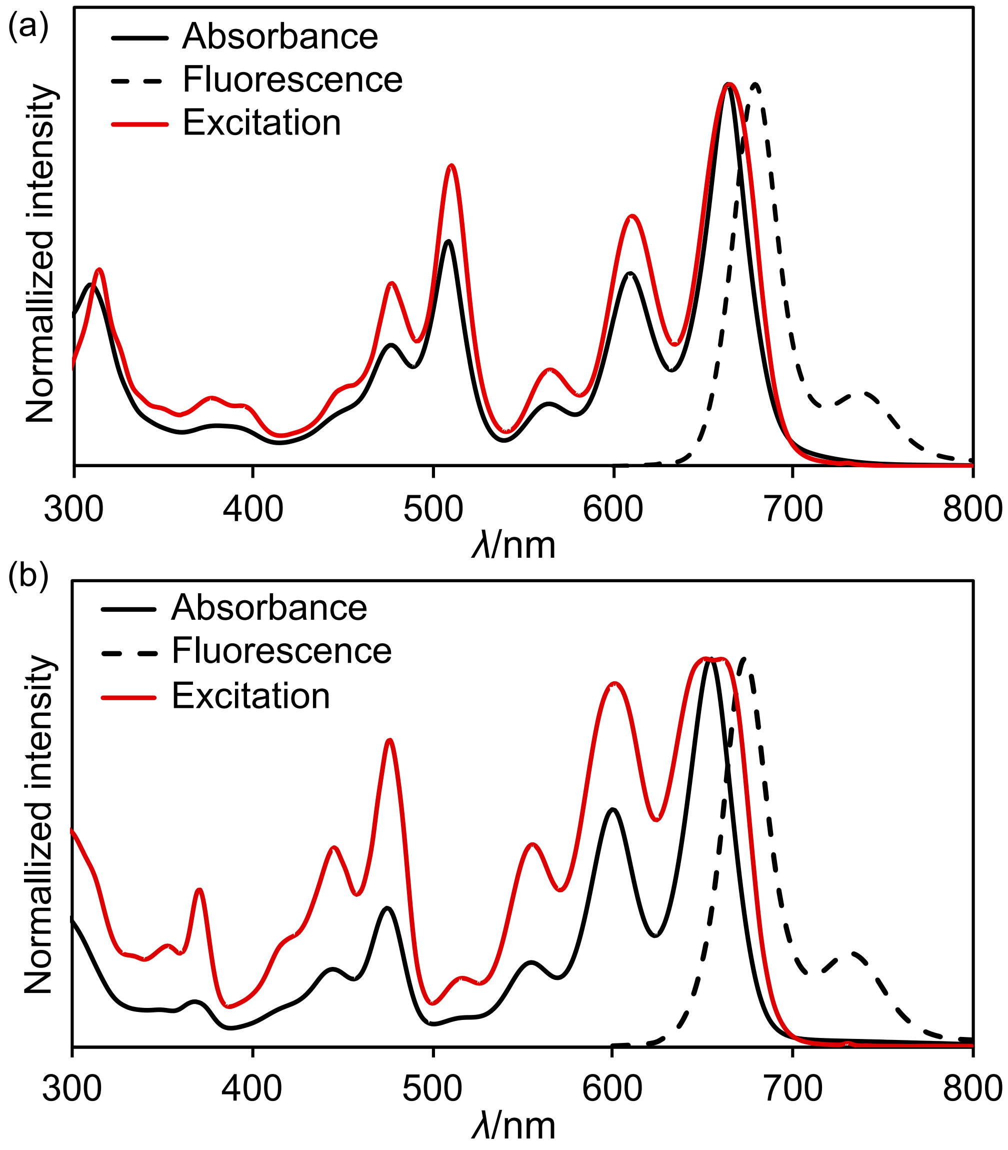

Figure S58. Absorption, fluorescence (λ_{ex} = 630 nm), and excitation spectra (λ_{em} = 730 nm) of (a) dianion **13** in DMSO and (b) dihydro-form **14a** in DMSO with one drop of TFA; λ = wavelength.

Table S3. Photophysical properties of **8a**, **13**, and **14a**.

	solvent	λ_{Abs}/nm	λ_{FL}/nm	Stokes shift/cm^{-1}	Φ_{FL}	τ/ns	$k_r/10^7$ s^{-1}	$k_{nr}+k_{ISC}/10^8$ s^{-1}
**8a**	CH_2Cl_2	633	664	738	0.13	2.0	6.5	5.0
**13**	DMSO	664	679	333	0.53	6.3	12	0.39
**14a**	DMSO[a]	655	673	408	0.40	4.6	8.7	1.3

[a]: with one drop of TFA.

10. OFET devices

For fabricating thin-film OFETs, the substrates modified with self-assembled monolayers (SAMs) of 12-cyclohexyldodecylphosphonic acid (CDPA) were used. SAM-modified substrates were prepared as described in literature[S6]. Heavily n-doped Si wafers with a 300 nm-thick thermally grown SiO_2 layer were used as substrates. The Si/SiO_2 substrates were cleaned with deionized water, acetone, and 2-propanol for 10 min in an ultrasonic bath. Substrates were dried with a flow of N_2 gas and then treated by UV–O_3 cleaner (Filgen UV253V8) for 45 min. A solution of $Al(NO_3)_3{\cdot}9H_2O$ in ethanol (0.1 M) was spin-coated (5000 rpm, 40 s) on cleaned substrates in an N_2 glovebox, and the substrates were annealed at 300 °C for 30 min in the air to form Al_2O_3 layer. The Al_2O_3-coated substrates were treated by UV–O_3 cleaner for 45 min and then soaked in a solution of CDPA in 2-propanol (1.5 mM) at room temperature for 12 h. Finally, substrates were washed with 2-propanol and deionized water, and then dried with a flow of N_2 gas to afford SAM-modified substrates. The average capacitance per unit area of the dielectric layer (C_i) of the insulating layer prepared by this method was measured to be 10 nF cm^{-2} [S2]. The active layer was prepared with the vacuum-deposition method using a solid of **8b** for 500 Å at 0.3–0.8 Å/s under the pressure of ~5×10^{-4} Pa. The top contact source and drain electrodes of gold films (thickness = 300 Å) were vacuum deposited under the pressure of ~5×10^{-4} Pa through a shadow mask on the active layer. The drain–source channel length (L) and width (W) were 50 μm and 1000 μm, respectively. The output and transfer characteristics of the OFETs were measured using a vacuum prober system (Thermal Block Company, SB-MCPS-NAT) and a Keithley 2400 semiconductor characterization system under vacuum at the pressure of ~3×10^{-1} Pa and atmospheric conditions. The field-effect mobilities (μ) of the OFETs were determined from the forward transfer curve in the saturation regime ($V_{DS} = 60$ V) and linear regime ($V_{DS} = 5$ V) using the following each equation,

$I_{DS} = \mu W C_i (V_G - V_{th})^2/2L$ (saturation regime)

$I_{DS} = \mu W C_i V_{DS}\{2(V_G - V_{th}) - V_{DS}\}/2L$ (linear regime)

Where I_{DS} is the drain–source current and V_{DS}, V_G, and V_{th} are the drain–source voltage, gate voltage, and threshold voltage, respectively. The on/off ratios (I_{on}/I_{off}) were determined from the I_{DS} at $V_G = 0$ V (I_{off}) and $V_G = 60$ V (I_{on}). The averaged μ values ($\mu_{average}$) of the thin-film OFETs were calculated from six devices for **8b**. The polarized optical microscope images were obtained using Zeiss Axio Scope.A1 microscope. The atomic force microscope images were obtained

using Shimadzu SPM-9700 in the tapping mode. Out-of-plane XRD measurement of the vacuum deposited film was performed using a Rigaku SmartLab X-ray diffractometer with a Cu-Kα source (λ = 1.5418 Å) in the 2θ scan mode at a fixed incidence angle of 0.58°. Photoelectron yield spectroscopy measurement was performed using a Riken Keiki AC-3 photoelectron spectrometer.

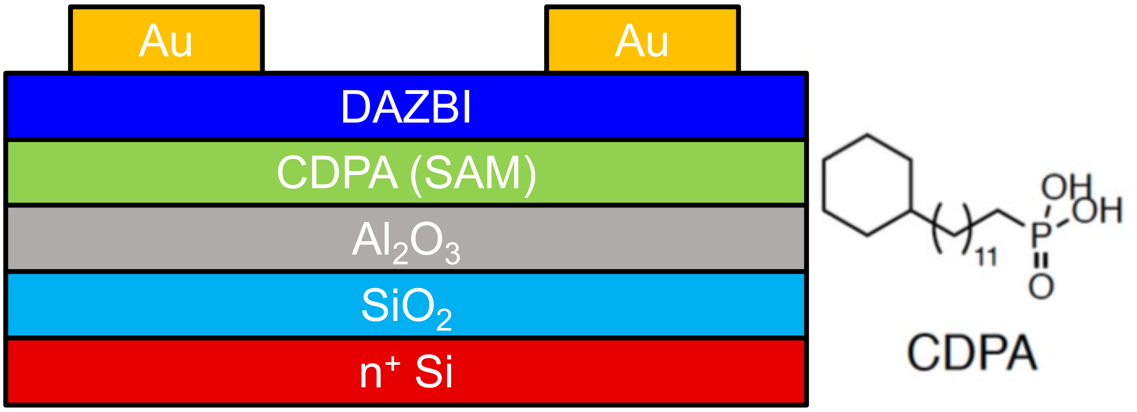

Figure S59. Schematic description of the thin-film OFET device composition.

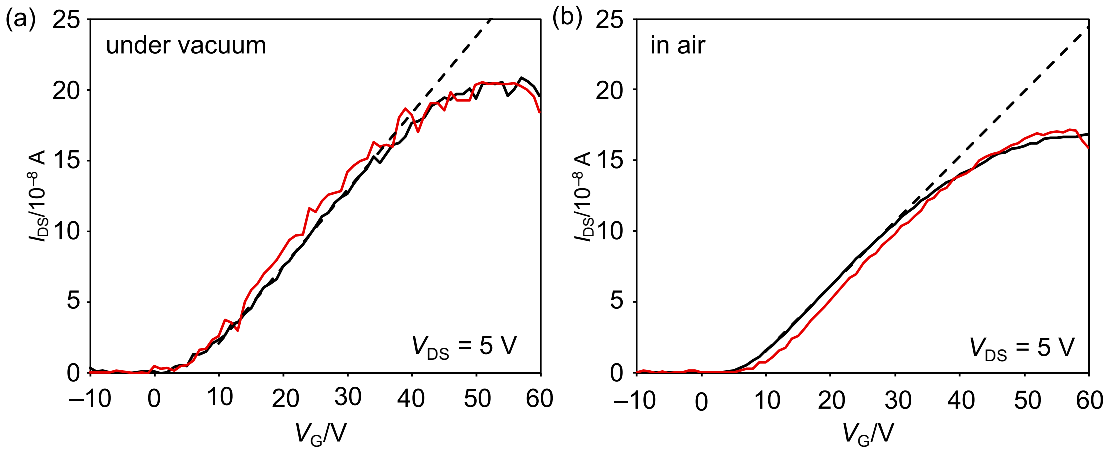

Figure S60. Transfer characteristics in the linear regime of thin-film OFET properties of **8b** under (a) vacuum and (b) ambient conditions. Black line: forward sweeping. Red line: backward sweeping.

Table S4. Properties of thin-film OFET devices of **8b**.

	Saturation regime		Linear regime	
	in vacuum	in air	in vacuum	in air
μ_{max} [$cm^2 V^{-1} s^{-1}$]	6.7×10^{-3}	5.4×10^{-3}	5.4×10^{-3}	4.8×10^{-3}
$\mu_{average}$ [$cm^2 V^{-1} s^{-1}$]	$(6.1\pm0.5)\times10^{-3}$	$(4.6\pm0.7)\times10^{-3}$	$(4.9\pm0.3)\times10^{-3}$	$(4.1\pm0.6)\times10^{-3}$
V_{th} [V]	2.5±0.5	3.8±1.8	6.2±1.5	7.5±1.9
I_{on}/I_{off} [-]	$(3.4\pm1.8)\times10^3$	$(5.2\pm9.7)\times10^3$	$(3.0\pm3.6)\times10^3$	$(3.3\pm5.3)\times10^3$

All potentials are standardized by ferrocene. [a]: in CH_2Cl_2 solution. [b]: in THF solution.

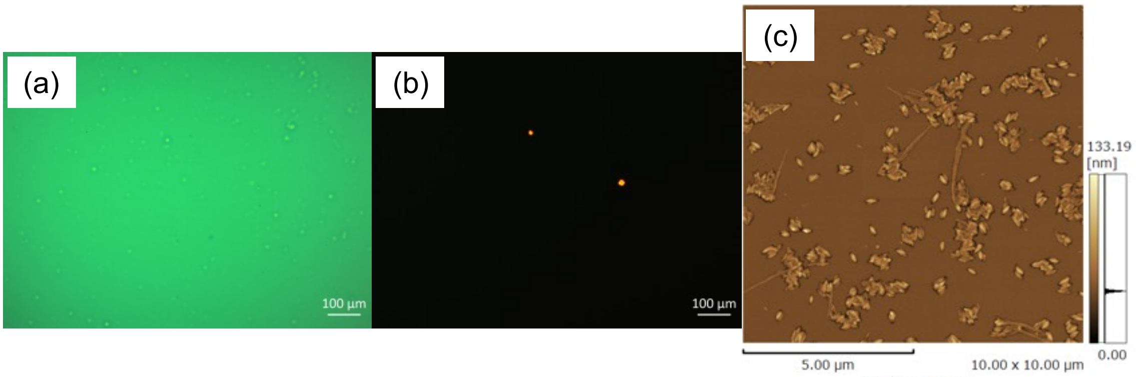

Figure S61. (a,b) Polarized optical micrographs and (c) AFM image of a vacuum-deposited film of **8b**. (RMS roughness: 8.5 nm)

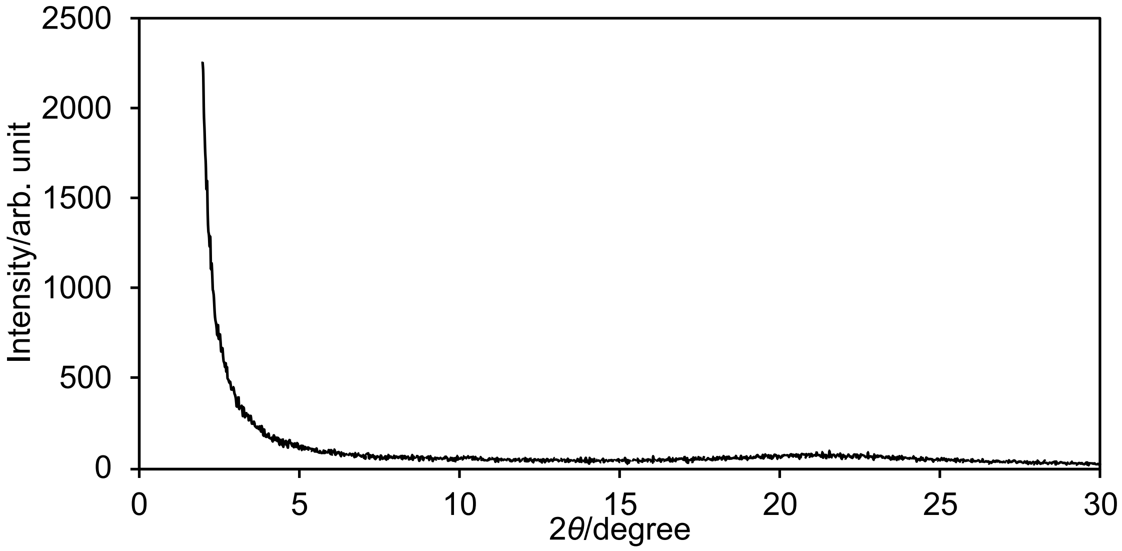

Figure S62. Thin film out-of-plane X-ray diffraction patterns of a vacuum-deposited film of **8b**.

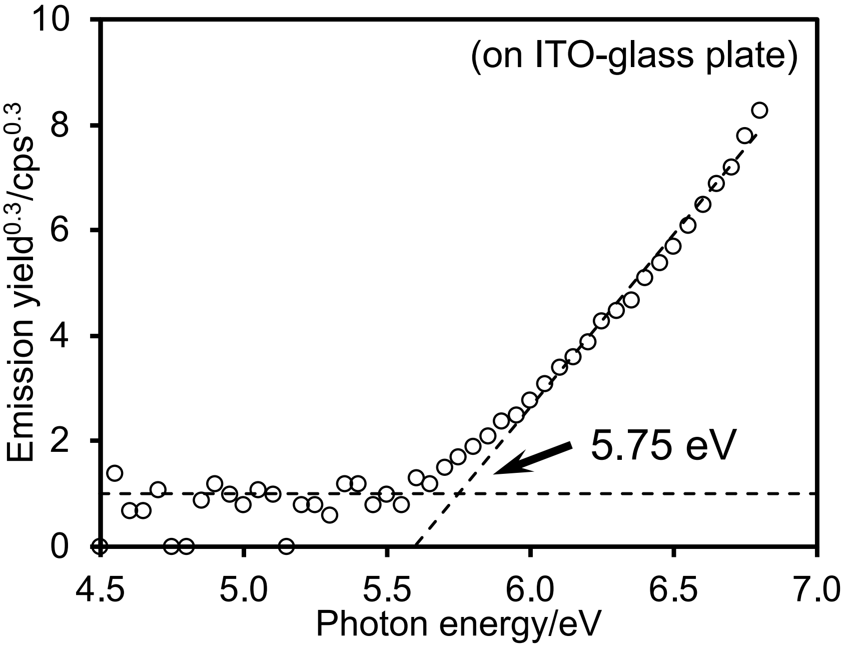

Figure S63. Photoelectron spectroscopy measurement of **8b** in a vacuum-deposited thin film.

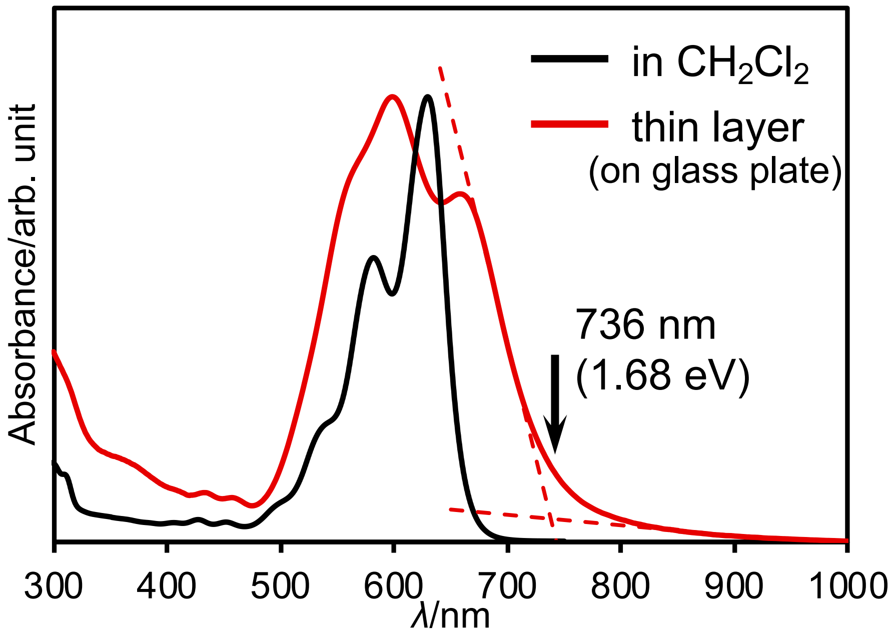

Figure S64. UV/vis absorption spectra of a vacuum-deposited film of **8b** and a CH_2Cl_2 solution of **8b**. λ = wavelength.

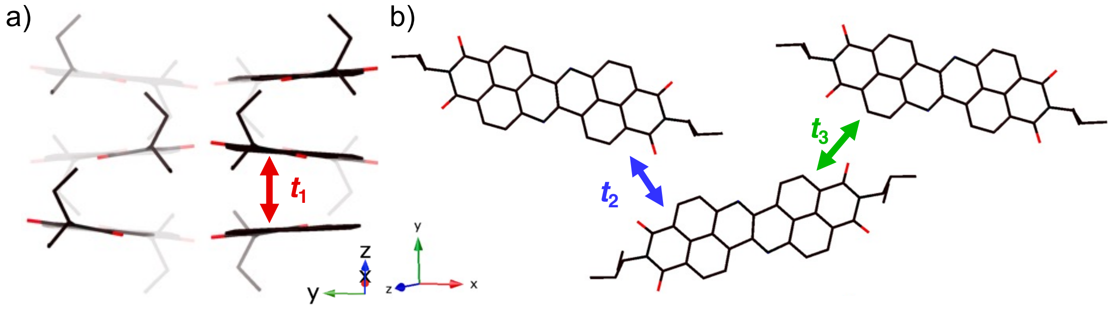

Figure S65. Packing pattern of **8b**. Arrows indicate the direction of transfer integrals. (a) Top view and (b) side view.

Table S5. Transfer integrals of the **8b** calculated at the PBEPBE/6-31G(d) level of theory.

	direction	**8b–A** and **8b–A**[a] [meV]	**8b–A** and **8b–B**[a] [meV]	**8b–B** and **8b–B**[a] [meV]
LUMO	t_1	+89.1	+7.62	+37.2
	t_2	+1.63	–0.38	+0.88
	t_3	+2.54	+5.31	+0.89
HOMO	t_1	+65.0	–6.56	+15.3
	t_2	+1.68	–0.92	–0.16
	t_3	+0.08	–0.07	–0.16

[a]: **8b–A** is one of the disordered structures with an occupancy of 0.58, and **8b–B** is another one with an occupancy of 0.42.

11. Other properties

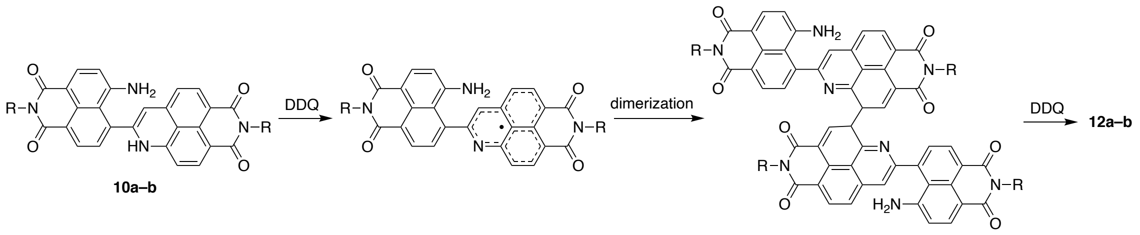

Scheme S1. Possible reaction mechanism of generation of **12a** and **12b**.

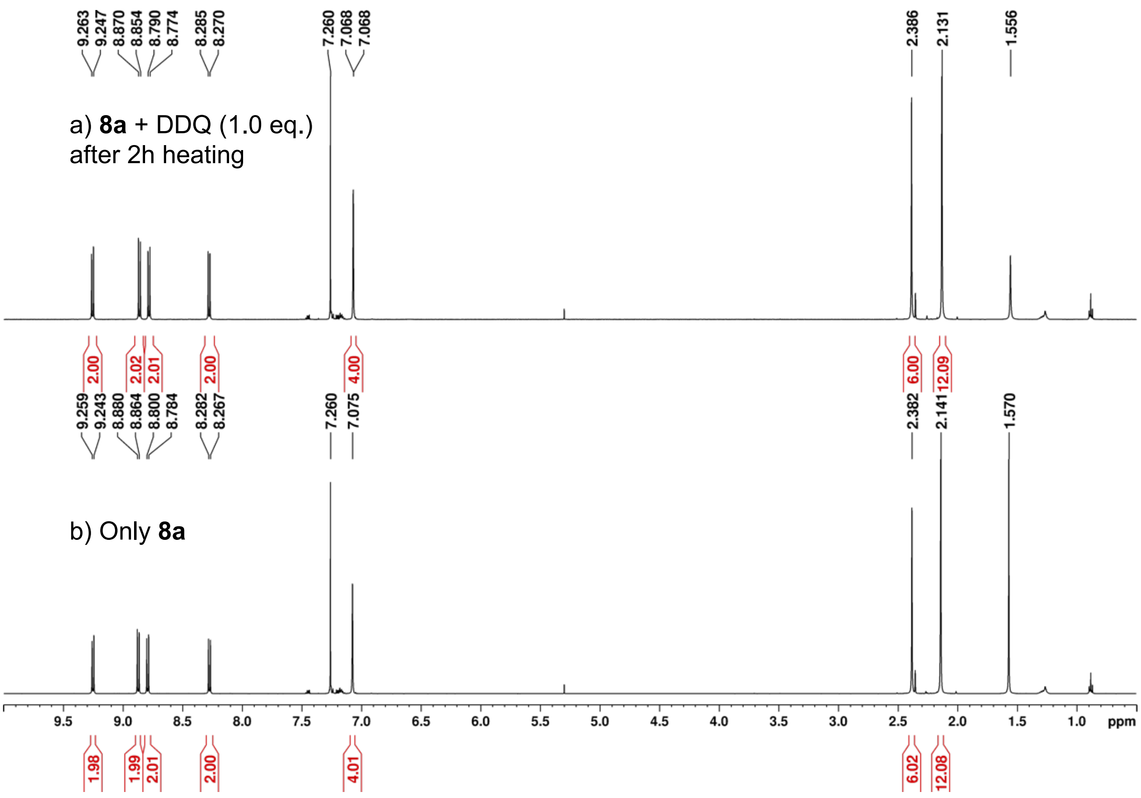

Figure S66. ^{1}H NMR spectra of **8a** in $CDCl_3$. (a) The spectrum after treatment with 1.0 equiv of DDQ at 60 °C. (b) The spectrum of the as-prepared sample.

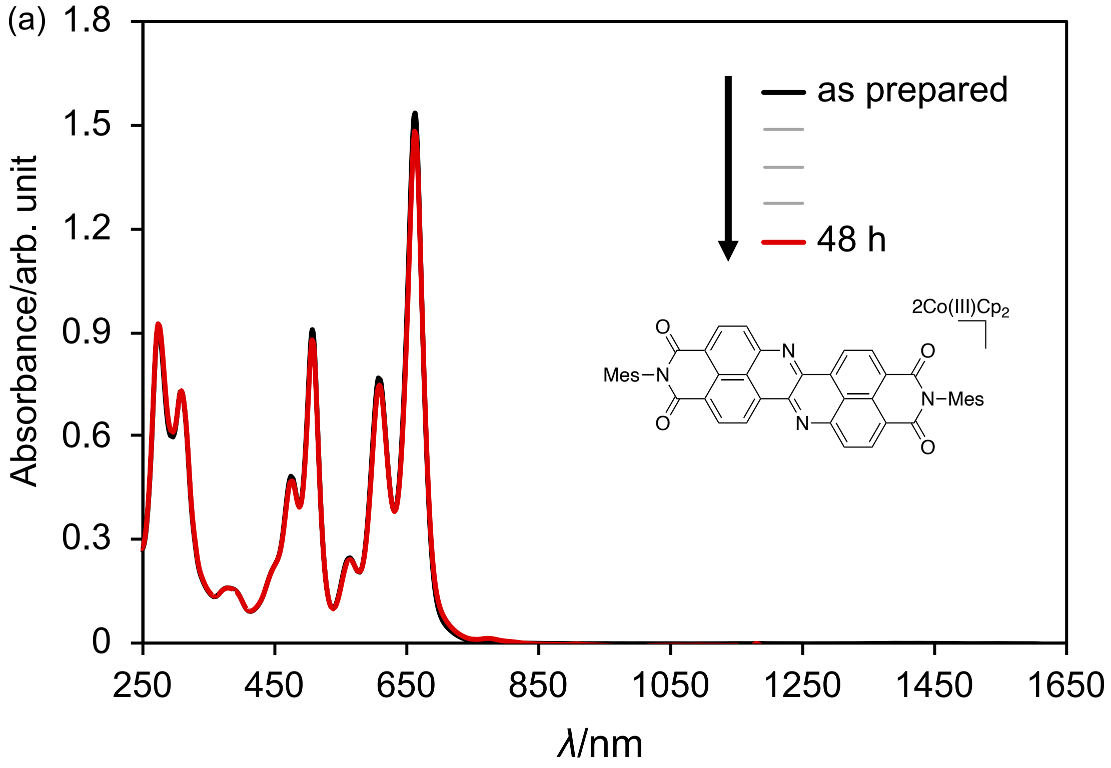

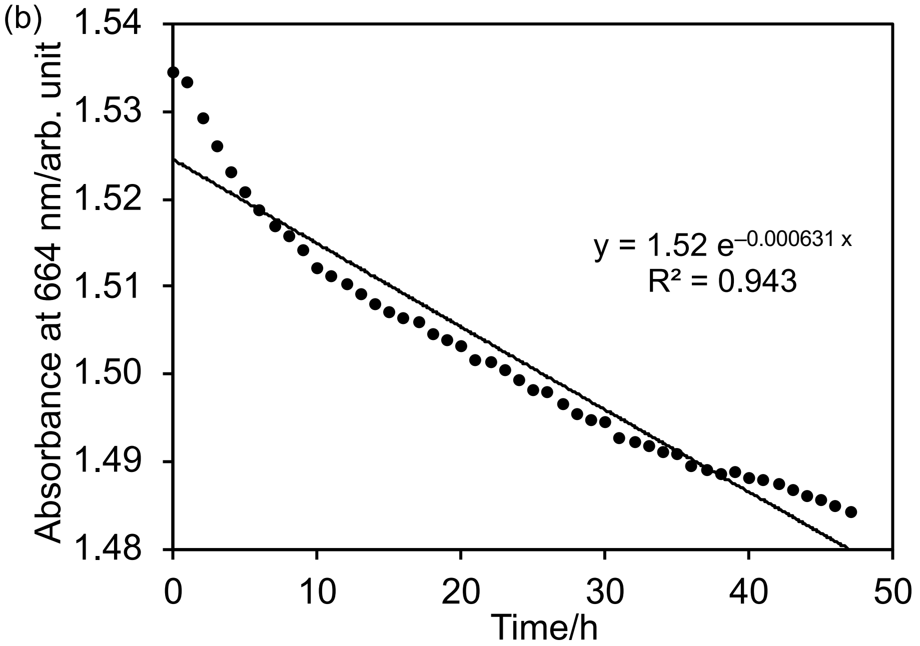

Figure S67. (a) Change in absorption spectra of **13** in DMSO under ambient conditions. (b) The plot of change in absorbance at 602 nm of **13** in DMSO under ambient conditions. Curve fitting was conducted by exponential approximation; λ = wavelength.

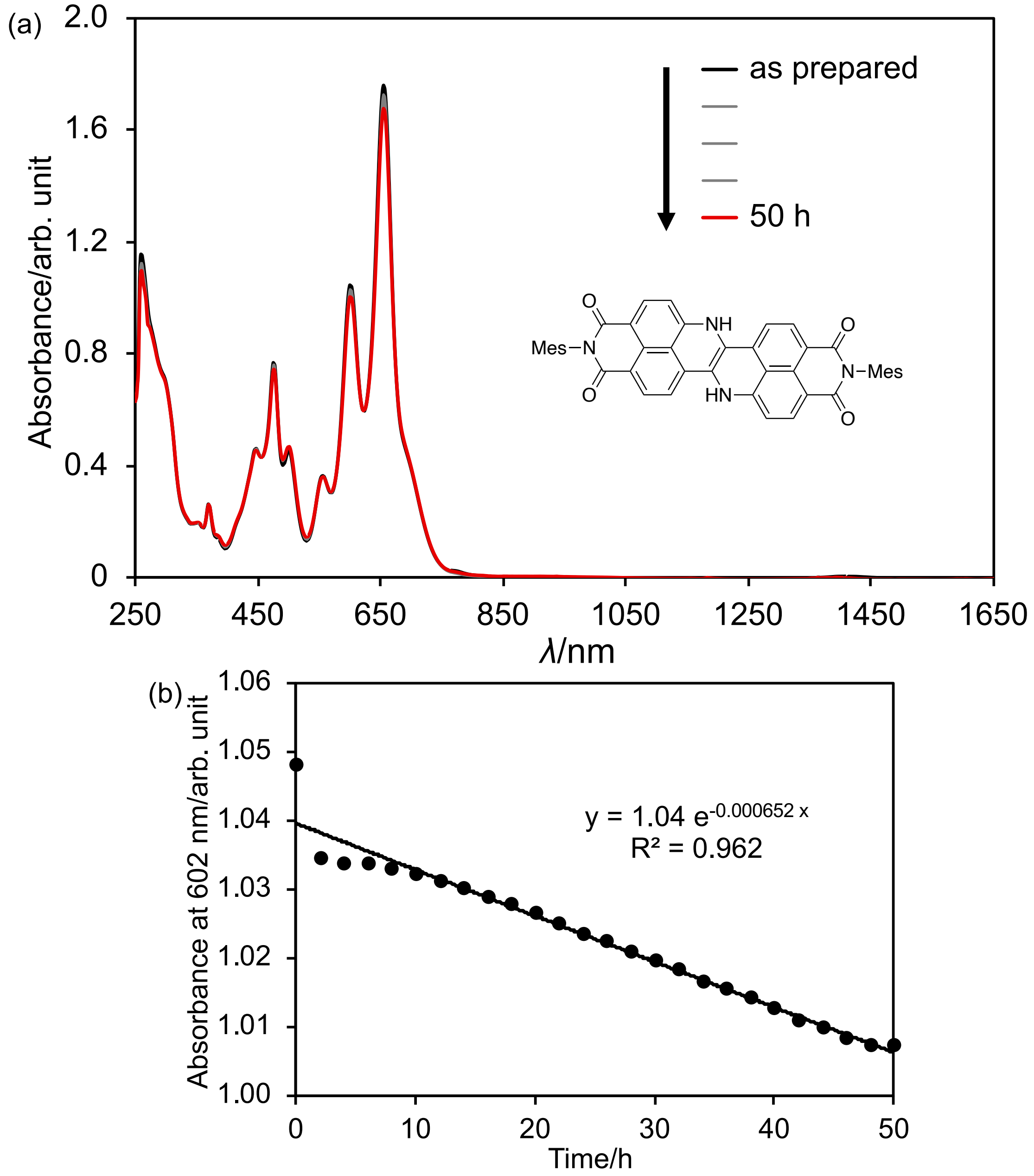

Figure S68. (a) Change in absorption spectra of **14a** in DMSO under ambient conditions. (b) The plot of change in absorbance at 602 nm of **14a** in DMSO under ambient conditions. Curve fitting was conducted by exponential approximation; λ = wavelength.

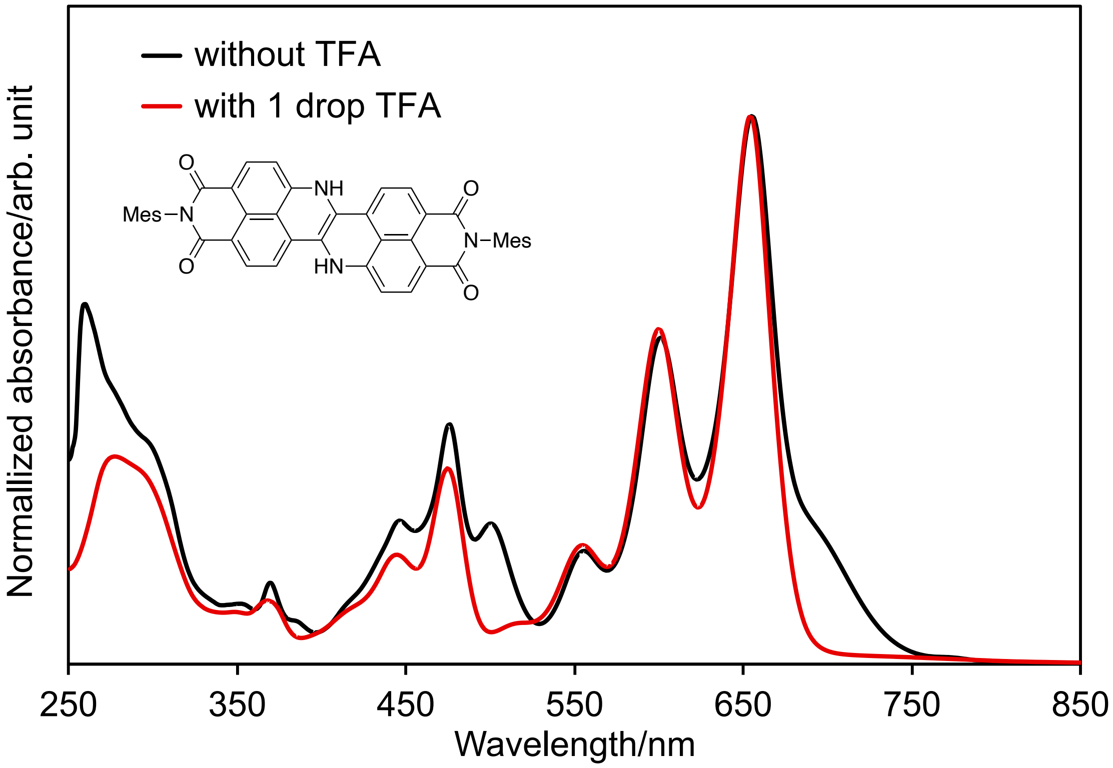

Figure S69. Absorption spectra of **13** in DMSO without TFA (black line) and with one drop of TFA (red line).

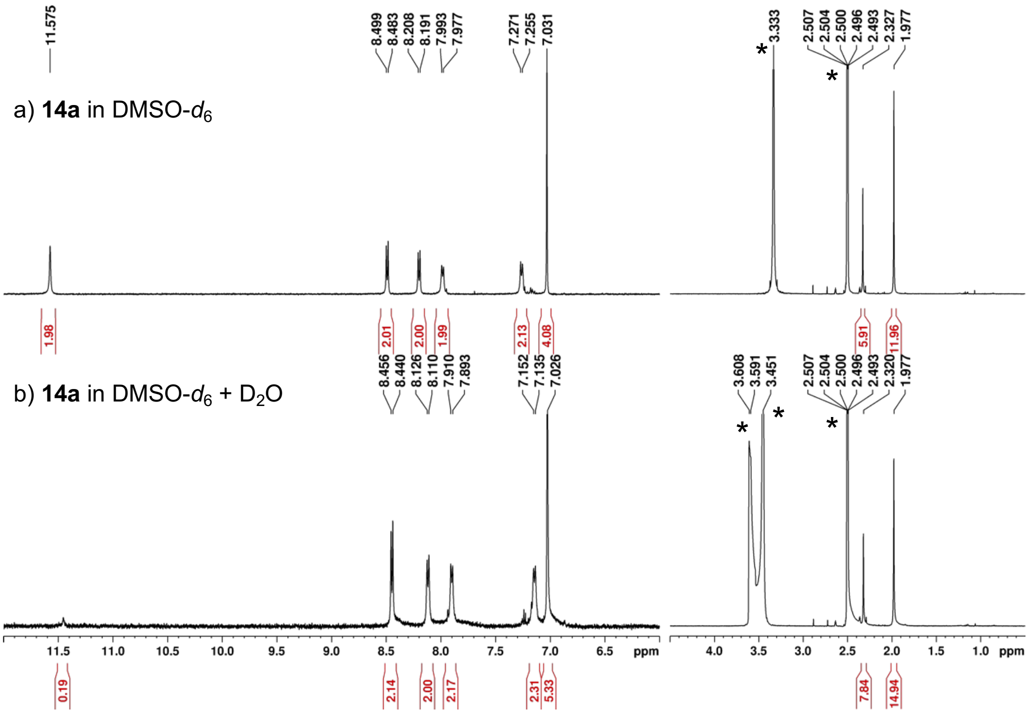

Figure S70. ^{1}H NMR spectra of **14a** in DMSO-d_6. (a) The spectrum of the as-prepared sample. (b) The spectrum after treatment with one drop of D_2O. *: residual solvent.

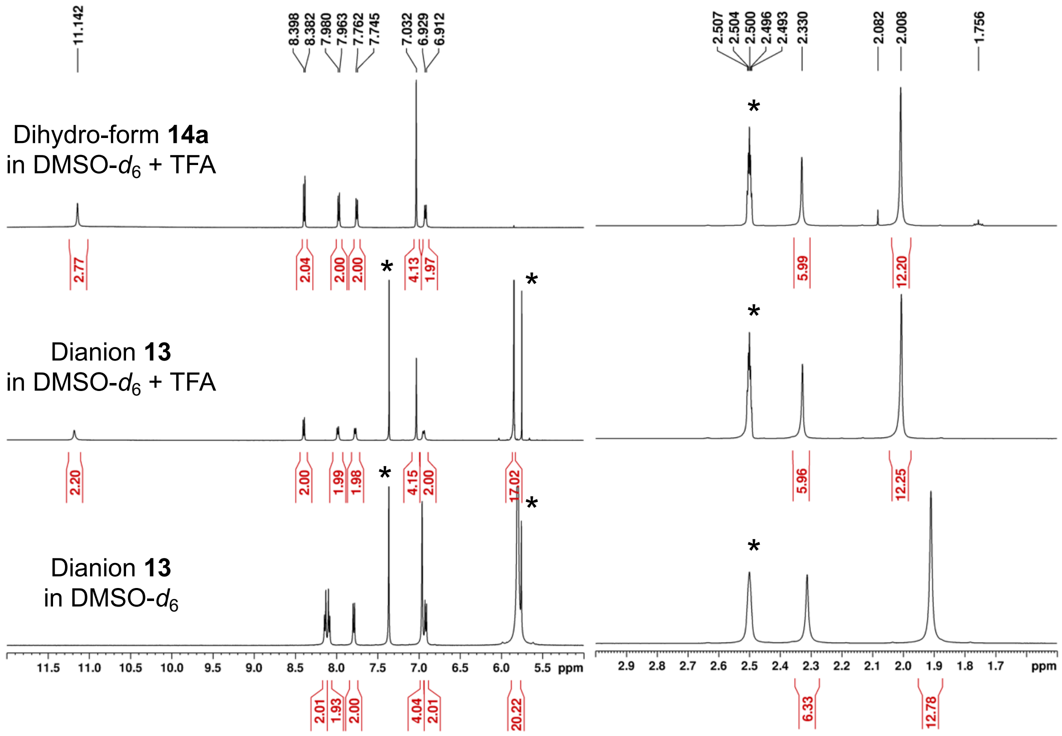

Figure S71. ^{1}H NMR spectra of **14a** and **13** in DMSO-d_6. (a) The spectrum of **14a** after treatment with one drop of TFA. (b) The spectrum of **13** after treatment with one drop of TFA. (c) The spectrum of the as-prepared sample of **13**. *: residual solvent.

Table S6. Cartesian coordinate of **8c**.

C	4.7443540	–1.2473402	–0.0001261
C	4.1669403	–2.5108199	–0.0002068
H	4.8199207	–3.3772714	–0.0003673
C	2.7733510	–2.6599701	–0.0000398
H	2.3168958	–3.6443297	0.0000572
C	1.9318663	–1.5421599	–0.0000463
C	1.6802127	0.9130251	–0.0001598
C	2.2662098	2.1824432	–0.0003814
H	1.6211490	3.0540233	–0.0005251
C	3.6582312	2.3221865	–0.0004786
H	4.1192765	3.3041924	–0.0007773
C	4.4856334	1.2027984	–0.0001763
C	3.9210171	–0.0926287	–0.0000792
C	2.5084021	–0.2363645	–0.0000133
C	6.2182647	–1.1045836	–0.0003046
C	5.9609268	1.3780233	–0.0000384
C	0.2323215	0.7021612	–0.0000587
C	–4.7443524	1.2473600	0.0000283
C	–4.1669324	2.5108339	0.0000382
H	–4.8199099	3.3772875	0.0001852
C	–2.7733423	2.6599703	–0.0001819
H	–2.3168747	3.6443245	–0.0003346
C	–1.9318645	1.5421533	–0.0001403
C	–1.6802120	–0.9130292	0.0000244
C	–2.2662115	–2.1824437	0.0002539
H	–1.6211584	–3.0540296	0.0003686
C	–3.6582360	–2.3221737	0.0004103
H	–4.1192815	–3.3041790	0.0007358
C	–4.4856396	–1.2027851	0.0001426
C	–3.9210211	0.0926386	–0.0000057
C	–2.5084036	0.2363608	–0.0001260
C	–6.2182540	1.1045741	0.0003214
C	–5.9609329	–1.3780354	0.0001332
C	–0.2323186	–0.7021681	–0.0000669
N	0.5675451	–1.7384932	–0.0000651
N	–0.5675439	1.7384854	–0.0001246
N	6.7301306	0.2029317	0.0008658
N	–6.7301267	–0.2029438	–0.0005302
O	6.9789402	–2.0629547	–0.0010832
O	6.4834274	2.4841218	–0.0003946
O	–6.9789487	2.0629424	0.0010960
O	–6.4834331	–2.4841227	0.0004921
C	8.1929368	0.3237131	0.0015732
H	8.6036640	–0.1719344	0.8843396
H	8.6041980	–0.1610106	–0.8870875
H	8.4378229	1.3832001	0.0078748
C	–8.1929332	–0.3237153	–0.0008938
H	–8.6039581	0.1609372	0.8879243
H	–8.6038786	0.1720828	–0.8834681
H	–8.4378675	–1.3831943	–0.0072381

No negative frequency

Sum of electronic and thermal free energies = –1595.457896 Hartree.

Table S7. Cartesian coordinate of **12c**.

C	-0.090742	0.060005	-0.039438
C	-0.065483	0.091163	3.673408
C	0.560501	0.075315	1.221026
C	-0.044203	-0.560139	2.412981
C	1.825846	0.702523	1.321825
C	-0.604512	-1.856637	2.311502
C	2.439618	1.301126	0.237557
C	-1.173902	-2.498454	3.394799
C	2.424681	1.884657	-2.152913
C	-1.775895	-2.50992	5.780576
C	1.787163	1.866745	-3.39079
C	-1.799881	-1.867805	7.016317
C	0.53414	1.266182	-3.532723
C	-1.264504	-0.585766	7.159249
C	-0.098229	0.667422	-2.437169
C	-0.689346	0.073303	6.066923
C	0.54026	0.669919	-1.170291
C	-0.650443	-0.567446	4.801972
C	1.808208	1.290502	-1.032204
C	-1.205112	-1.865602	4.663113
C	-1.40989	0.031982	-2.54028
C	-0.119455	1.414665	6.171672
C	-3.27185	-0.573038	-3.784448
C	0.383807	3.306746	7.415848
C	-3.929438	-0.574251	-5.020509
C	0.338156	3.967978	8.649212
C	-5.186666	-1.174633	-5.165752
C	0.881255	5.250703	8.797762
C	-5.809918	-1.786204	-4.084316
C	1.481172	5.894508	7.722251
C	-5.781443	-2.423582	-1.705089
C	2.156786	5.886602	5.353434
C	-5.135198	-2.436944	-0.472721
C	2.216647	5.235445	4.124681
C	-3.883132	-1.833168	-0.315741
C	1.668018	3.958795	3.96368
C	-3.257846	-1.204722	-1.398183
C	1.048762	3.311828	5.038536
C	-3.899135	-1.192236	-2.661927
C	0.990825	3.956366	6.299207
C	-5.171236	-1.803172	-2.818202
C	1.545028	5.25378	6.458979
C	-1.951068	-0.556704	-1.302132
C	0.45689	1.978903	4.938206
C	3.75748	1.958281	0.41479
C	-1.757717	-3.849712	3.225738
C	3.754603	2.527226	-2.013976
C	-2.351189	-3.872868	5.648594
C	-7.138711	-2.420096	-4.261195
C	2.057141	7.248153	7.895036
C	-7.11152	-3.065831	-1.850945
C	2.743387	7.244382	5.495828
C	5.644236	3.172981	-0.619065

C	-2.866132	-5.795747	4.187035
C	-9.015281	-3.665886	-3.245339
C	3.219385	9.174973	6.958914
N	-1.331231	-0.520993	-0.148205
N	0.457461	1.357095	3.784827
N	-2.034663	0.024115	-3.692058
N	-0.154923	2.043175	7.320867
N	-7.70173	-3.022578	-3.121115
N	2.652533	7.834614	6.766991
N	4.335182	2.518975	-0.737406
N	-2.298209	-4.454387	4.37013
O	4.31546	2.022484	1.502532
O	-1.788135	-4.431103	2.148766
O	-7.722145	-2.424382	-5.336411
O	2.029577	7.850431	8.959693
O	4.33706	3.049844	-2.953939
O	-2.847694	-4.462692	6.597841
O	-7.68573	-3.613409	-0.919819
O	3.277496	7.821947	4.559255
H	-1.28386	-0.079084	8.117592
H	-2.243858	-2.385455	7.859923
H	-0.583111	-2.37986	1.364916
H	2.346865	0.711767	2.26962
H	1.726057	3.45139	3.007711
H	2.700889	5.740583	3.295895
H	0.84691	5.763535	9.753215
H	-0.127135	3.457802	9.486201
H	-5.624692	-2.928273	0.36142
H	-3.378059	-1.853197	0.643036
H	-5.69581	-1.175759	-6.123668
H	-3.436956	-0.098785	-5.862372
H	0.029601	1.25253	-4.492296
H	2.284894	2.330036	-4.236149
H	3.646708	9.492745	6.010787
H	2.435314	9.865648	7.27827
H	3.986596	9.142877	7.736012
H	-2.097582	-6.471981	3.805758
H	-3.231562	-6.135321	5.153279
H	-3.681152	-5.756932	3.460296
H	-9.336851	-3.565112	-4.279221
H	-8.937192	-4.719309	-2.966581
H	-9.728292	-3.183944	-2.57204
H	5.561351	4.222259	-0.912305
H	5.963896	3.089424	0.416929
H	6.360586	2.685678	-1.284536

No negative frequency

Sum of electronic and thermal free energies = –3189.719267 Hartree.

12. References

S1. K. Tajima, K. Matsuo, H. Yamada, S. Seki, N. Fukui, H. Shinokubo, *Angew. Chem, Int. Ed.* **2021**, *60*, 14060–14067.

S2. S. Hayakawa, K. Matsuo, H. Yamada, N. Fukui, H. Shinokubo, *J. Am. Chem. Soc.* **2020**, *142*, 11663–11668.
